# Noncanonical contribution of microglial transcription factor NR4A1 to post-stroke recovery through TNF mRNA destabilization

**DOI:** 10.1371/journal.pbio.3002199

**Published:** 2023-07-24

**Authors:** Pinyi Liu, Yan Chen, Zhi Zhang, Zengqiang Yuan, Jian-Guang Sun, Shengnan Xia, Xiang Cao, Jian Chen, Cun-Jin Zhang, Yanting Chen, Hui Zhan, Yuexinzi Jin, Xinyu Bao, Yue Gu, Meijuan Zhang, Yun Xu

**Affiliations:** 1 Department of Neurology, Nanjing Drum Tower Hospital, Affiliated Hospital of Medical School and the State Key Laboratory of Pharmaceutical Biotechnology, Nanjing University, Nanjing, China; 2 The Brain Science Center, Beijing Institute of Basic Medical Sciences, Beijing, China; 3 Center of Alzheimer’s Disease, Beijing Institute for Brain Disorders, Beijing, China; 4 Jiangsu Province Stroke Center for Diagnosis and Therapy, Nanjing, China; 5 Nanjing Neurology Clinic Medical Center, Nanjing, China; 6 Institute of Brain Sciences, Nanjing University, Nanjing, China; 7 Jiangsu Key Laboratory for Molecular Medicine, Medical School of Nanjing University, Nanjing, China; UCSD, UNITED STATES

## Abstract

Microglia-mediated neuroinflammation is involved in various neurological diseases, including ischemic stroke, but the endogenous mechanisms preventing unstrained inflammation is still unclear. The anti-inflammatory role of transcription factor nuclear receptor subfamily 4 group A member 1 (NR4A1) in macrophages and microglia has previously been identified. However, the endogenous mechanisms that how NR4A1 restricts unstrained inflammation remain elusive. Here, we observed that NR4A1 is up-regulated in the cytoplasm of activated microglia and localizes to processing bodies (P-bodies). In addition, we found that cytoplasmic NR4A1 functions as an RNA-binding protein (RBP) that directly binds and destabilizes *Tnf* mRNA in an N6-methyladenosine (m^6^A)-dependent manner. Remarkably, conditional microglial deletion of *Nr4a1* elevates *Tnf* expression and worsens outcomes in a mouse model of ischemic stroke, in which case NR4A1 expression is significantly induced in the cytoplasm of microglia. Thus, our study illustrates a novel mechanism that NR4A1 posttranscriptionally regulates *Tnf* expression in microglia and determines stroke outcomes.

## Abstract 2

Microglia-mediated neuroinflammation is involved in neurological diseases such as ischemic stroke, but the mechanisms that prevent unstrained inflammation are unclear. This study reveals that the canonical transcription factor NR4A1 has a novel role in microglia as an RNA-binding protein that localizes to processing bodies to destabilize pro-inflammatory transcript and alleviate ischemic brain injury.

## Introduction

Microglia are the resident macrophages in the brain, which exert critical physiological functions during development and homeostasis, such as scanning of the brain microenvironment, remodeling of synapses, and support for the development of oligodendrocytes [1,2]. In most neurological diseases, however, microglia respond rapidly to brain injuries and undergo distinct morphological and transcriptional changes [3,4]. In ischemic stroke, microglia are first activated after ischemia, releasing proinflammatory cytokines and chemokines to initiate early proinflammatory response and trigger the subsequent infiltration of peripheral immune cells to damage the brain [5–7]. Thus, a stronger mechanistic understanding of the regulation of neuroinflammation by microglia during ischemic stroke could pave the way for a novel therapy that would reduce ischemic brain injury.

NR4A1 (also referred to as Nur77, TR3 and NGFI-B and encoded by the gene *Nr4a1*) is a member of the nuclear hormone receptor superfamily, and the expression of NR4A1, as an immediate early gene, is rapidly induced in response to various stimuli [8]. Similar to other classical nuclear hormone receptors, NR4A1 contains a transactivation domain, DNA-binding domain (DBD), and ligand-binding domain (LBD). In peripheral tissues, NR4A1 acts as an anti-inflammatory molecule in macrophages, and *Nr4a1*-deficiency enhances the polarization of macrophages into a proinflammatory phenotype and exacerbates atherosclerosis [9,10]. It was recently found that NR4A1 is also key for the down-regulation of isocitrate dehydrogenase and the prevention of succinate accumulation, which induces metabolic reprogramming and attenuates chronic inflammation [11]. In the mouse brain, NR4A1 protects dopaminergic neurons in a rodent model of Parkinson’s disease and delays the onset of clinical symptoms of experimental autoimmune encephalomyelitis (EAE) by inhibiting microglial activation and proinflammatory gene expression [12,13]. It has also been reported that NR4A1 in myeloid cells could recruit the corepressor CoREST to the Th (tyrosine hydroxylase) promoter, repressing the production of norepinephrine, which limited the progression of EAE [14].

Given that NR4A1 suppresses proinflammatory response in macrophages and microglia, we investigated its underlying regulatory mechanisms in microglia. Intriguingly, we found that the transcription factor NR4A1 exerted a novel function as an RNA-binding protein (RBP) in microglia. That is, cytoplasmic NR4A1 was up-regulated and localized to processing bodies (P-bodies) after microglial activation, directly binding and promoting the destabilization of *Tnf* mRNA in an N6-methyladenosine (m^6^A)-dependent manner. Moreover, trichostatin A (TSA), a pan-histone deacetylase (HDAC) inhibitor, increased NR4A1 expression and accelerated *Tnf* mRNA decay in microglia. Similar to in vitro findings, the expression of NR4A1 was remarkably induced, mainly in the cytoplasm of microglia, in a middle cerebral artery occlusion (MCAO) mouse model and stroke patients. Global and conditional microglial knockout of *Nr4a1*-enhanced *Tnf* expression and remarkably exacerbated ischemic brain injury. This study identified a key role of microglial NR4A1 as an RBP that posttranscriptionally regulates *Tnf* expression, providing a promising target for stroke treatment.

## Results

### NR4A1 localizes to P-bodies in activated microglia

**Fig 1:**
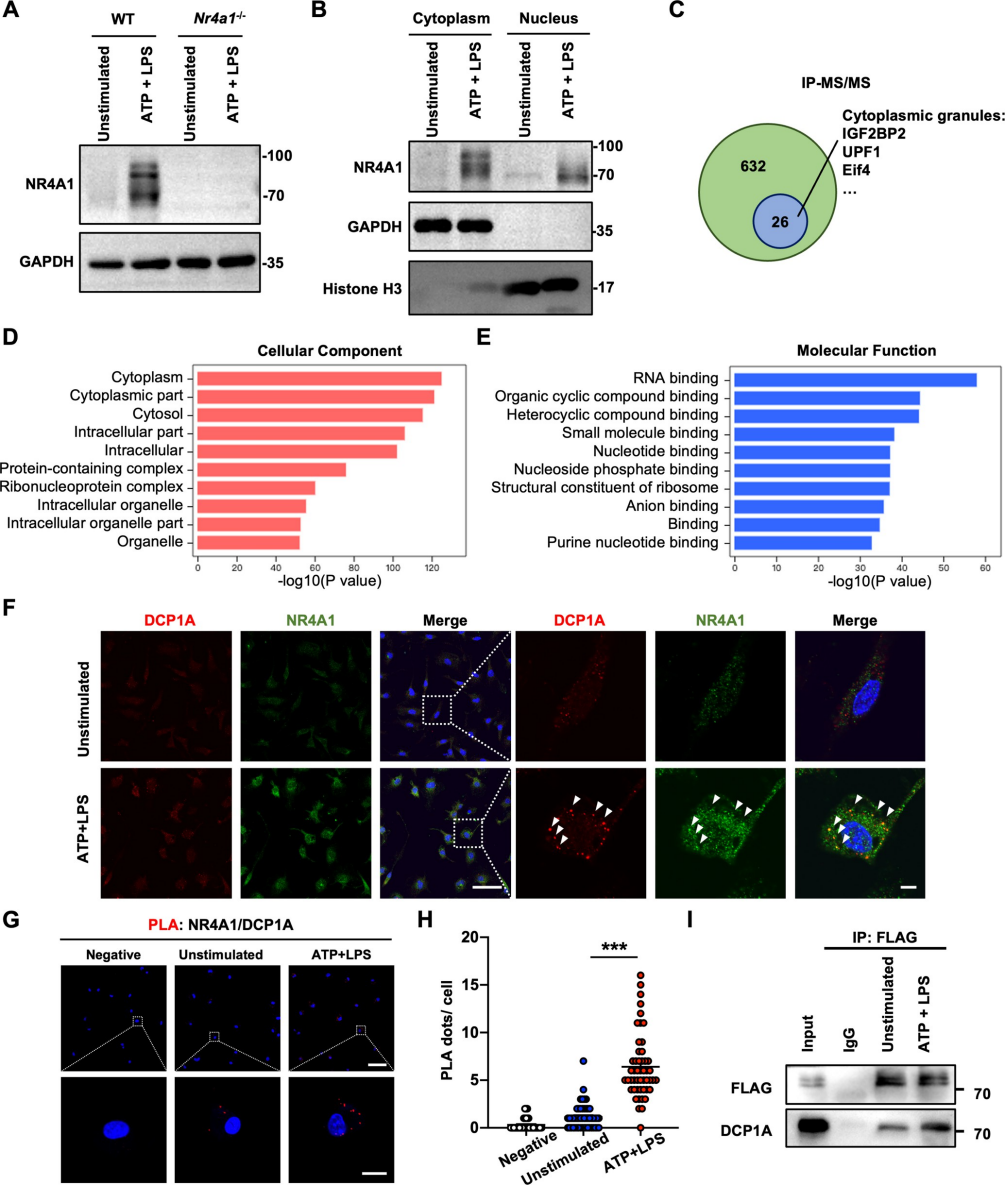
NR4A1 localizes to P-bodies in microglia. **(A)** Immunoblot analysis of NR4A1 in WT and *Nr4a1*^-/-^ primary microglia treated with or without ATP (1 mM) + LPS (100 ng/ml). **(B)** Immunoblot analysis of NR4A1 expression in the cytoplasmic and nuclear fractions of untreated and ATP+LPS-treated primary microglia. **(C)** Co-IP-MS/MS analysis of Flag-NR4A1-interacting proteins. **(D, E)** Representative GO terms of cellular components and molecular functions enriched in Flag-NR4A1-interacting proteins. **(F)** Representative images of untreated and ATP+LPS-treated primary microglia showing colocalization of NR4A1 with DCP1A. Scale bar, 50 and 5 μm. **(G, H)** In situ proximity ligation assay assessing the interactions between NR4A1 and DCP1A (red) in negative control, unstimulated and ATP+LPS-treated primary microglia. Scale bar, 50 and 10 μm (*n* = 50 cells counted in each group). **(I)** Immunoblot analysis of DCP1A in BV2 cell (expressing FLAG-NR4A1) lysates immunoprecipitated with an anti-FLAG antibody. Data are presented as mean ± SEM. In (**H**), one-way ANOVA with post hoc Dunnett’s test. ***P < 0.001. The underlying data for this figure can be found in S1 Data. The original blot for this figure can be found in S1 Raw Images. Co-IP, coimmunoprecipitation; WT, wild-type.

NR4A1 is expressed in microglia and exhibits anti-inflammatory effect under inflammatory stimuli [12]. However, the underlying mechanism is still unclear. Injured neurons release various damage-associated molecular patterns (DAMPs), which activates microglia through Toll-like receptors and purinergic receptors [15,16], we therefore used ATP and LPS to activate primary microglia and confirmed that NR4A1 was significantly up-regulated after ATP treatment and ATP+LPS co-stimulation (**Figs 1A** and **S1A**). Notably, we found that ATP+LPS stimulation induced an upper band of NR4A1 as compared to ATP alone (**S1A Fig**). Since NR4A1 is a canonical transcription factor with its nuclear localization, and previous studies also have demonstrated that NR4A1 is present in the cytoplasm of neurons and PC12 cells in its phosphorylated form [17,18], we further examined the subcellular localization of NR4A1 in microglia after ATP+LPS treatment. Western blot analysis of compartmentalized proteins showed that ATP+LPS stimulation resulted in an up-regulation of an upper band of NR4A1 in the cytoplasm but not the nucleus (**Fig 1B**).

To investigate how NR4A1 functions in activated microglia, we screened for proteins that interact with NR4A1 in ATP+LPS-treated BV2 cells overexpressing FLAG-NR4A1 by MS/MS. Intriguingly, 26 out of 632 identified proteins were components of cytoplasmic granules, including P-bodies (**Fig 1C** and **S1 Table**). In addition, GO analysis suggested that NR4A1-interacting proteins are predominantly enriched in the cytoplasm and possess RNA-binding capacity, which also indicated microglial NR4A1 might function in cytoplasmic RNA granules (**Fig 1D** and **1E**). Furthermore, Immunofluorescence staining revealed that NR4A1 colocalized with DCP1A, a key component of P-bodies (**Fig 1F**). To confirm the direct interaction between NR4A1 and P-bodies, we performed in situ proximity ligation assays and obtained consistent results (**Fig 1G** and **1H**). Additionally, we confirmed that ATP+LPS enhanced the interactions between FLAG-NR4A1 and the P-body marker DCP1A by coimmunoprecipitation (Co-IP) (**Fig 1I**), suggesting that NR4A1 in activated microglia might function in P-bodies.

### NR4A1 functions as an RNA-binding protein that promotes *Tnf* mRNA degradation

**Fig 2:**
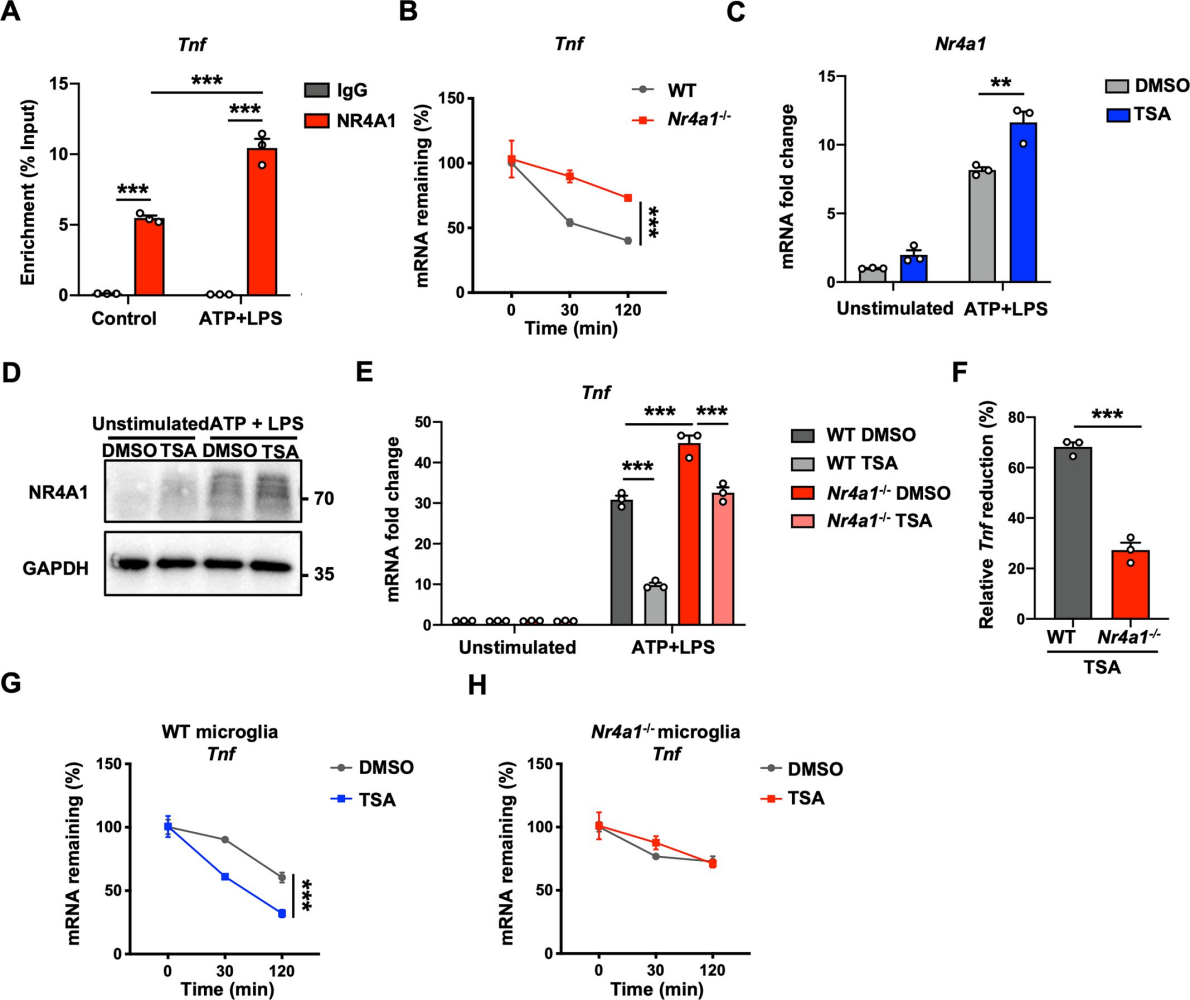
NR4A1 functions as an RBP that promotes *Tnf* mRNA degradation. **(A)** RIP analysis of NR4A1-bound *Tnf* mRNA in untreated and ATP+LPS-treated BV2 cells (*n* = 3 biological repeats in each group). **(B)** RNA stability assay of *Tnf* mRNA in ATP+LPS-activated WT and *Nr4a1*^-/-^ primary microglia at the indicated time points after actinomycin D treatment (*n* = 4 biological repeats in each group). **(C, D)** mRNA (**C**) (*n* = 3 biological repeats in each group) and protein (**D**) levels of *Nr4a1* in unstimulated and ATP+LPS-activated primary microglia treated with DMSO or TSA (50 nM). **(E)** *Tnf* mRNA levels in unstimulated and ATP+LPS-treated primary microglia treated with DMSO or TSA (50 nM) (*n* = 3 biological repeats in each group). **(F)** Relative reduction in *Tnf* expression induced by TSA treatment in ATP+LPS-treated WT and *Nr4a1*^-/-^ primary microglia (calculated from **E**) (*n* = 3 biological repeats in each group). **(G, H)** RNA stability assay of *Tnf* mRNA in ATP+LPS-treated WT and *Nr4a1*^-/-^ primary microglia treated with TSA at the indicated time points after actinomycin D treatment (*n* = 3 biological repeats in each group). Data are presented as mean ± SEM. In (**A**), (**C**), (**E**), two-way ANOVA with post hoc Bonferroni’s test. In (**B**), (**G**), (**H**), two-way ANOVA. In (**F**), two-tailed unpaired Student’s *t* test. *P < 0.05; **P < 0.01; ***P < 0.001. The underlying data for this figure can be found in S1 Data. The original blot for this figure can be found in S1 Raw Images. RBP, RNA-binding protein; TSA, trichostatin A; WT, wild-type.

Since P-bodies are cytoplasmic granules that mediate RNA decay, and because NR4A1 has 2 highly conserved Cys_4_ zinc fingers in its DBD, which might have RNA-binding capacity, we hypothesized that NR4A1 might function as an RBP that regulates the stability of mRNA [19,20]. We performed RBP immunoprecipitation sequencing (RIP-seq) to determine NR4A1-RNA associations. The RIP-seq data showed that NR4A1 bound to several transcripts that are related to inflammatory response (**S2 Table**). Additionally, since NR4A1 is reported to be capable of suppressing proinflammatory response in microglia, it was reasonable to assume that NR4A1 might regulate the mRNA stability of proinflammatory factors. Accordingly, RIP-qPCR analysis confirmed that NR4A1 directly bound *Tnf*, *Il1b*, *Il6*, and *Il10* mRNA, and these interactions substantially increased after ATP+LPS treatment (**Figs 2A** and **S2A–S2C**). We next found that *Nr4a1* knockout led to significantly greater stabilization of *Tnf* mRNA in activated microglia treated with actinomycin D, a commonly used transcription inhibitor, than in wild-type (WT) microglia, but did not affect *Il1b*, *Il*6, or *Il10* (**Figs 2B** and **S2D–S2F**).

We also used TSA, a pan-histone deacetylase (HDAC) inhibitor that has been reported to induce NR4A1 expression, to pharmacologically up-regulate NR4A1 in microglia (**Fig 2C** and **2D**) [21]. We found that TSA treatment significantly suppressed *Tnf* mRNA expression in primary microglia (**Fig 2E**). However, *Nr4a1* knockout significantly attenuated the reduction of *Tnf* mediated by TSA (**Fig 2E** and **2F**). Furthermore, we investigated whether TSA suppresses *Tnf* expression via the mechanism described above. We examined mRNA stability after TSA treatment and found that TSA remarkably promoted the mRNA degradation of *Tnf*, but this effect was not observed in *Nr4a1*-knockout microglia (**Fig 2G** and **2H**).

However, we also found that NR4A1-overexpression could suppress the activity of *Tnf* promoter by luciferase assay (**S2G Fig**), which was consistent with the previous study [22], indicating that NR4A1 functions as a transcription factor to regulate *Tnf* expression. Thus, to investigate the function of cytoplasmic NR4A1, we overexpressed wildtype-NR4A1 and NR4A1 with 3XNES (nuclear export sequence) in BV2 cells (**S2H–S2J Fig**). We found that wildtype-NR4A1 down-regulated *Tnf*, *Il1b*, and *Il6* after ATP+LPS stimulation, whereas cytoplasmic NR4A1 (NR4A1-NES) specifically regulated the expression level of *Tnf* mRNA and promoted its degradation (**S2K–S2M Fig**). Collectively, these data demonstrate that cytoplasmic NR4A1 specifically promotes *Tnf* mRNA degradation through directly binding to *Tnf* mRNA in microglia.

### NR4A1 promotes *Tnf* mRNA degradation in an m^6^A-dependent manner

**Fig 3:**
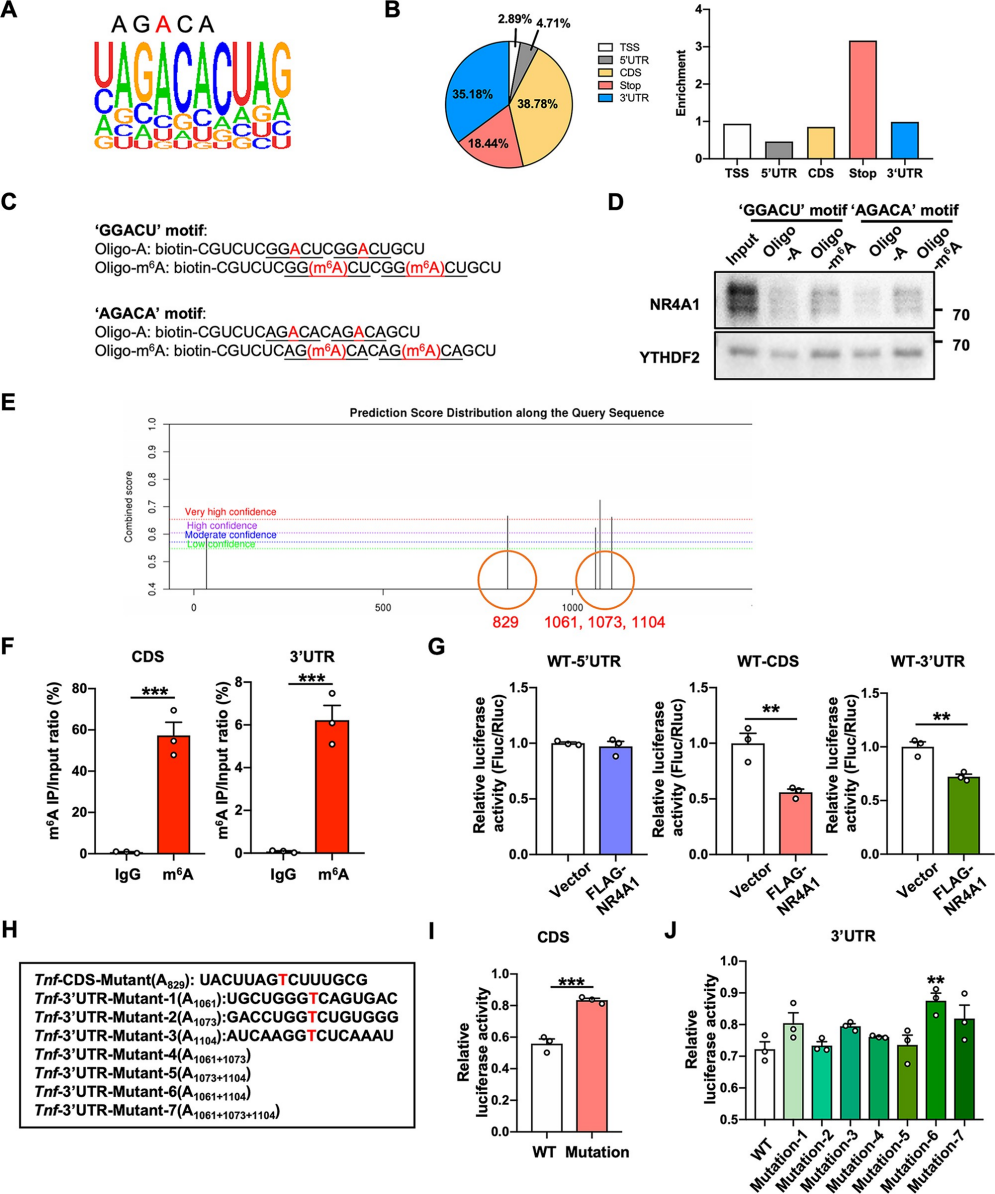
NR4A1 promotes *Tnf* mRNA degradation in an m^6^A-dependent manner. **(A)** Binding motif identified by HOMER with NR4A1-binding peaks. **(B)** The distribution (left) and enrichment (right) of NR4A1-binding peaks identified by RIP-seq. **(C, D)** Immunoblot analysis of NR4A1 in the cytoplasmic fraction of ATP+LPS-treated primary microglia pulled down by m^6^A-containing or unmethylated oligonucleotides. **(E)** SRAMP software prediction of the m^6^A sites in *Tnf* mRNA. **(F)** m^6^A-RIP-qPCR analysis of m^6^A enrichment in the CDS and 3′ UTR of *Tnf* mRNA in BV2 cells (*n* = 3 biological repeats in each group). **(G)** Luciferase activities of the 5′ UTR, CDS, and 3′ UTR of *Tnf* in HEK293T cells overexpressing NR4A1 or empty vector (*n* = 3 biological repeats in each group). **(H)** m^6^A site mutations in the CDS and 3′ UTR of *Tnf* mRNA. **(I, J)** Relative luciferase activities of WT or mutant CDS of *Tnf* (**I**) and WT or mutant 3′ UTR of *Tnf* (**J**) in HEK293T cells overexpressing NR4A1 (normalized to Fluc/Rluc in HEK293T cells with empty vector) (*n* = 3 biological repeats in each group). Data are presented as mean ± SEM. In (**F**), (**G**), (**I**), two-tailed unpaired Student’s *t* test. In (**J**), one-way ANOVA with post hoc Dunnett’s test. *P < 0.05; **P < 0.01; ***P < 0.001. The underlying data for this figure can be found in S1 Data. The original blot for this figure can be found in S1 Raw Images. RIP-seq, RNA-binding protein immunoprecipitation sequencing; WT, wild-type.

m^6^A modification, one of the key mechanisms through which mRNAs are posttranscriptionally regulated, impacts fundamental aspects of RNA and plays critical roles in various biological processes, including inflammation [23–25]. In addition, several m^6^A readers (YTHDF2, IGF2BPs, etc.) were previously reported to be localized in cytoplasmic granules and regulate mRNA stability [26,27]. Here, we found that an “AGACA” sequence, which is consistent with the “DRACH” m^6^A core motif (D = G/A/U, R = G/A, H = A/U/C), was enriched in NR4A1-binding peaks (**Fig 3A**) [28]. Moreover, NR4A1-binding sites were highly enriched around stop codons and in 3′ UTR, coinciding with the distribution of m^6^A sites (**Fig 3B**) [28]. Thus, we hypothesized that NR4A1 can promote *Tnf* mRNA degradation in an m^6^A-dependent manner. To verify our hypothesis, we used a methylated RNA probe and an unmethylated RNA probe (“GGACU” and “AGACA” motif) for RNA pull-down and found that endogenous NR4A1 showed higher binding affinity for the methylated probe than for the unmethylated probe (**S3A, S3C and S3D Fig**). Moreover, RNA electrophoretic mobility shift assay (REMSA) also validated that recombinant NR4A1 preferentially bound to the methylated probe (**S3B Fig**), suggesting that NR4A1 is a potential m^6^A-binding protein.

To determine whether *Tnf* mRNA bears m^6^A sites, we used SRAMP software to predict m^6^A sites in *Tnf* mRNA [29]. We identified 4 very high- or high-confidence m^6^A sites in the CDS region and 3′ UTR of *Tnf* mRNA (**Fig 3E**), and m^6^A-RIP-qPCR analysis confirmed that m^6^A modification indeed occurs in the CDS and 3′ UTR of *Tnf* mRNA (**Fig 3F**). To further confirm whether NR4A1 directly bind the m^6^A-modified *Tnf* mRNA, we performed PAR-CLIP and found that NR4A1 could bind to the m^6^A-modified regions in the CDS and 3′ UTR of *Tnf* mRNA (**S3C Fig**). We next inserted the WT 5′ UTR, CDS, or 3′ UTR of *Tnf* mRNA into a firefly luciferase reporter. As expected, NR4A1 overexpression significantly reduced the luciferase activity of the WT CDS and 3′ UTR but did not affect that of the 5′ UTR (**Fig 3G**). Furthermore, mutations in the m^6^A sites of the CDS and 3′ UTR (A829, A1061+A1104) partly reversed the effect of NR4A1 on the luciferase activity of the WT CDS and 3′ UTR (**Fig 3H–3J**). In addition, we also overexpressed NR4A1-NES in *Mettl3*-knockout BV2 cells (**S3D Fig**). The results showed that cytoplasmic NR4A1 had no effect on the expression level of *Tnf* in m^6^A-deficient BV2 cells (**S3E Fig**). Taken together, our data indicate that m^6^A modification of *Tnf* mRNA is the potential mechanism accounting for cytoplasmic NR4A1-mediated *Tnf* mRNA degradation.

### Microglial NR4A1 is up-regulated in both mice and patients with ischemic stroke

**Fig 4:**
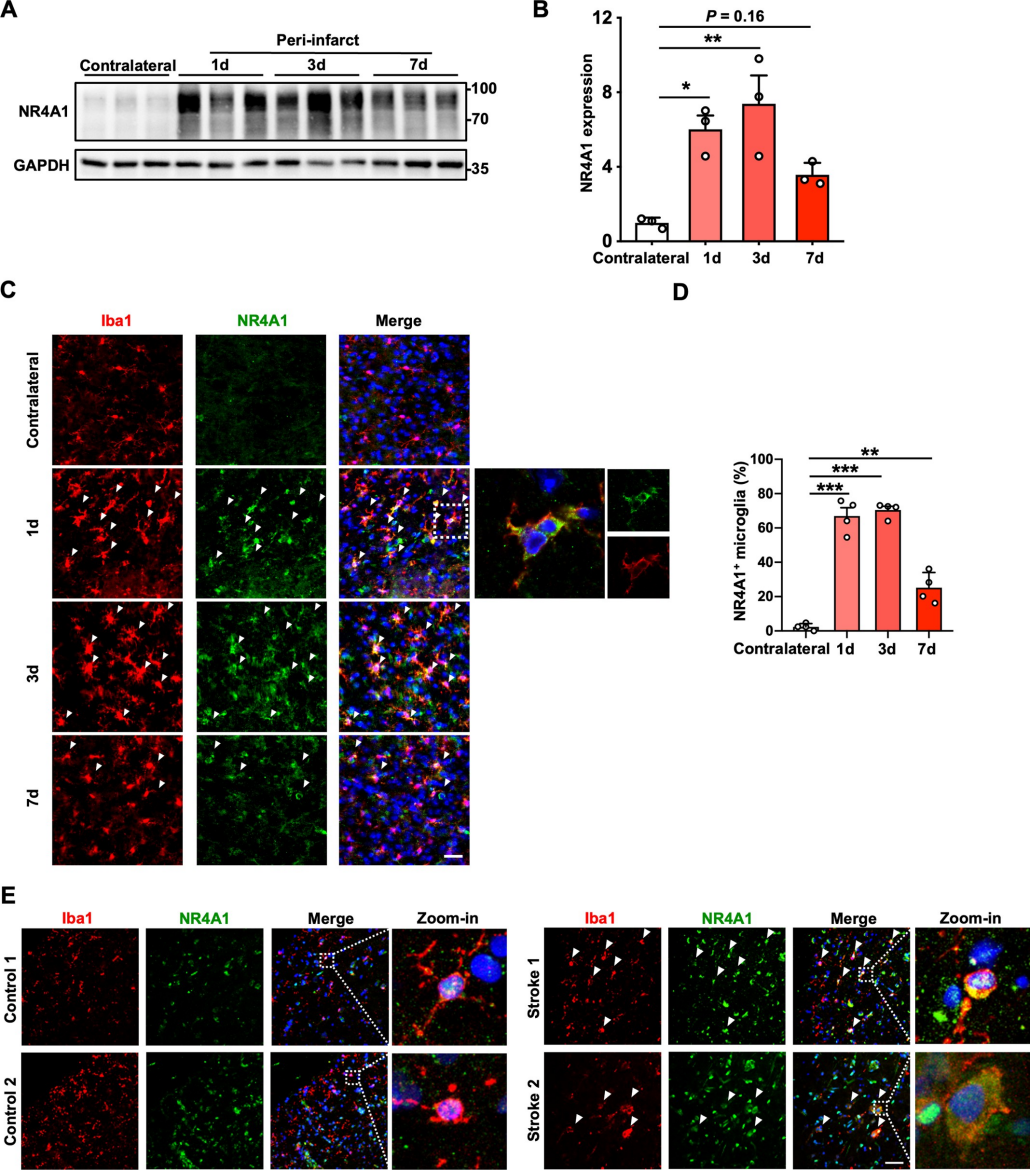
Microglial NR4A1 is up-regulated in both mice and patients with ischemic stroke. **(A)** Immunoblot analysis of NR4A1 in contralateral and peri-infarct tissue 1 day, 3 days, and 7 days after MCAO. **(B)** Quantification of immunoblot analysis of NR4A1 expression in contralateral and peri-infarct tissue 1 day, 3 days, and 7 days after MCAO showing that brain ischemia induced NR4A1 expression in peri-infarct tissue (*n* = 3 mice in each group). **(C)** Representative images of the contralateral and peri-infarct cortices of mice 1 day, 3 days, and 7 days after MCAO showing localization of NR4A1 (green) in Iba1^+^ (red) microglia. The inset shows a digitally magnified field of a single cell from the image. Scale bar, 30 μm. **(D)** Quantification of the percentage of NR4A1^+^ microglia showing that MCAO-induced NR4A1 expression in microglia in the peri-infarct cortex (*n* = 4 mice in each group). **(E)** Images of human brain samples from 2 non-stroke patients and 2 stroke patients. The inset shows a digitally magnified field of a single cell from the image. Stroke-induced NR4A1 expression in human microglia. Scale bar, 50 μm. Data are presented as mean ± SEM. In (**B**) and (**D**), one-way ANOVA with post hoc Dunnett’s test. *P < 0.05; **P < 0.01; ***P < 0.001. The underlying data for this figure can be found in S1 Data. The original blot for this figure can be found in S1 Raw Images. MCAO, middle cerebral artery occlusion.

Microglia in the ischemic penumbra region release proinflammatory cytokines to initiate early inflammatory response [30,31]. To characterize the expression pattern of NR4A1 in ischemic stroke, we used MCAO as a mouse model to mimic ischemic stroke in human and found that NR4A1 expression was significantly elevated in the penumbra at day 1 and day 3 post-stroke (**Fig 4A** and **4B**). Moreover, the number of NR4A1^+^ microglia surrounding the infarct area peaked 1 to 3 days after MCAO (**Fig 4C** and **4D**). Specifically, NR4A1 was predominantly located in the cytoplasm of microglia, but not in the nucleus (**Fig 4C**). To assess whether this expression pattern can also be observed in stroke patients, we analyzed NR4A1 expression in postmortem brain sections obtained from patients after ischemic stroke by immunofluorescence staining. Our data showed that in the peri-infarct tissue, more NR4A1 was localized in microglia in stroke patients than in non-stroke controls (**Fig 4E**). Thus, we found that microglial NR4A1 was significantly up-regulated in the cytoplasm at the acute phase after ischemic stroke, suggesting cytoplasmic NR4A1 might play an important role in the pathophysiology of ischemic stroke.

### *Nr4a1*-knockout enhances *Tnf* expression and exacerbates brain injury in experimental ischemic stroke

**Fig 5:**
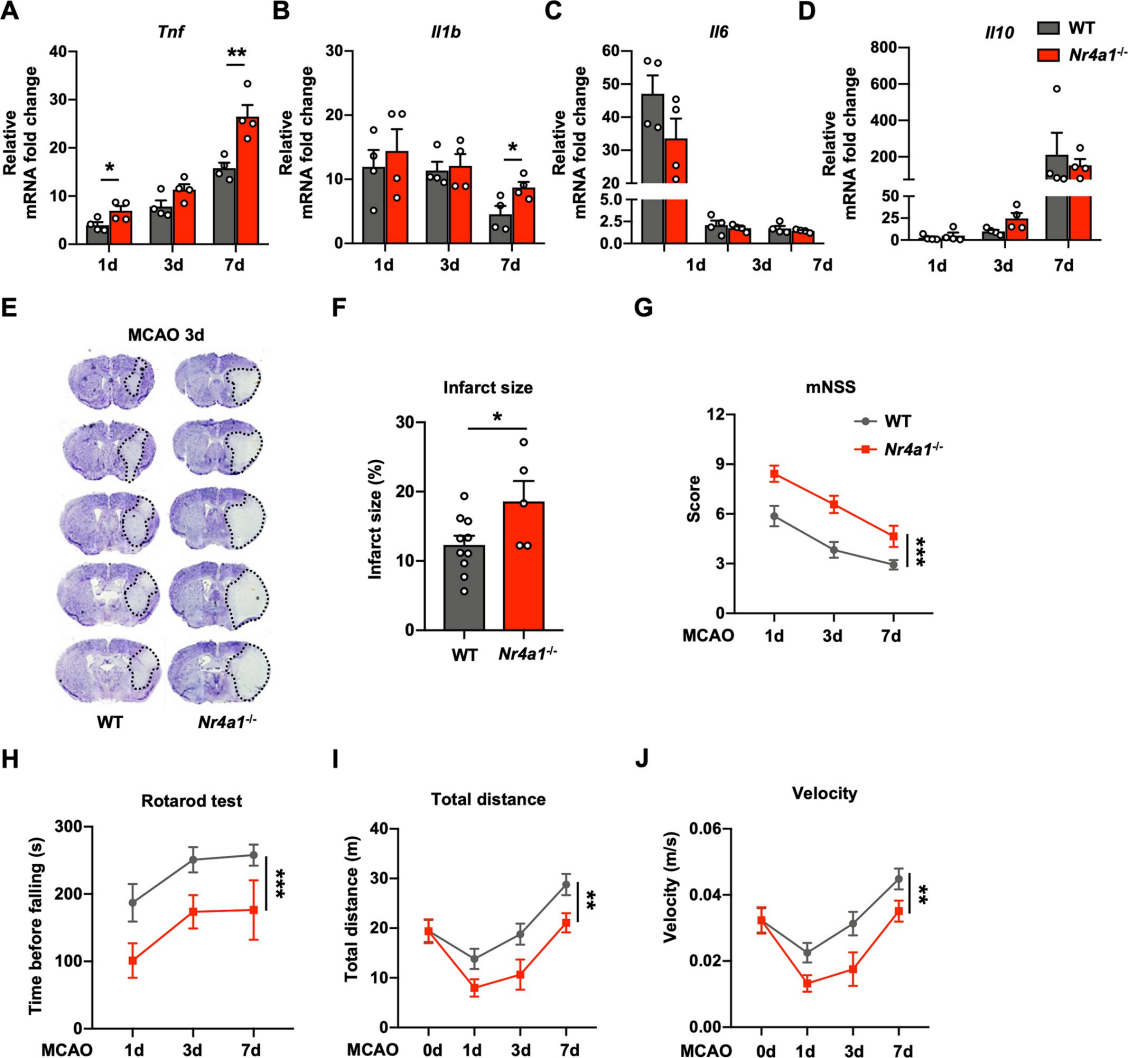
*Nr4a1*-knockout enhances *Tnf* expression and exacerbates brain injury in experimental ischemic stroke. **(A–D)** Relative mRNA levels of several inflammatory factors in the ipsilateral hemispheres of WT and *Nr4a1*^-/-^ mice 1 day, 3 days, and 7 days after MCAO compared with those in the contralateral hemispheres (*n* = 4 mice in each group). **(E)** Representative images of Nissl staining of tissues from WT and *Nr4a1*^-/-^ mice 3 days after MCAO. **(F)** Quantification of the infarct size, as measured by Nissl staining (*n* = 10 for WT mice, *n* = 5 for *Nr4a1*^-/-^ mice). **(G–J)** Sensorimotor deficits were aggravated in *Nr4a1*^-/-^ mice compared to WT mice 1 day, 3 days, and 7 days after MCAO, as determined by the mNSS test (**G**) (*n* = 15–38 for WT mice, *n* = 14–28 for *Nr4a1*^-/-^ mice), rotarod test (**H**) (*n* = 14–18 for WT mice, *n* = 7–16 for *Nr4a1*^-/-^ mice), total distance traveled (**I**) (*n* = 12–30 for WT mice, *n* = 9–21 for *Nr4a1*^-/-^ mice), and mean velocity (**J**) (*n* = 12–40 for WT mice, *n* = 9–28 for *Nr4a1*^-/-^ mice). Data are presented as mean ± SEM. In (**A–D**) and (**F**), two-tailed unpaired Student’s *t* test. In (**G–J**), two-way ANOVA. *P < 0.05; **P < 0.01; ***P < 0.001. The underlying data for this figure can be found in S1 Data. MCAO, middle cerebral artery occlusion; WT, wild-type.

To investigate whether NR4A1 impacts disease progression, experimental ischemic stroke was induced in WT and *Nr4a1*^-/-^ mice by MCAO. We first explored the putative effect of NR4A1 on the expression profiles of inflammatory factors after ischemia. Compared to those from WT mice, brain tissues from *Nr4a1*^*-/-*^ mice exhibited higher mRNA expression of *Tnf* (1 day: *p* < 0.05, 7 days: *p* < 0.01) (**Fig 5A**). Although *Il1b* expression also elevated at day 7 post-stroke (**Fig 5B**), there was no significant difference in the mRNA levels of *Il6* and *Il10* between *Nr4a1*^*-/-*^ mice and WT mice (**Fig 5C** and **5D**).

Next, the infarct size was evaluated by Nissl staining of brain slices 3 days after stroke. We found that the infarct size was significantly larger in *Nr4a1*^-/-^ mice than in WT mice (**Fig 5E** and **5F**). In addition, several behavioral tests were conducted to explore the effects of NR4A1 on neurological deficits after stroke. Notably, *Nr4a1*^-/-^ mice had more severe neurological deficits than WT controls, as assessed by the modified neurological severity score (mNSS) test, the rotarod test, total distance traveled, and mean velocity 1, 3, and 7 days after stroke (**Fig 5G–5J**). Taken together, these data suggest that *Nr4a1-*knockout significantly promoted *Tnf* expression and exacerbated brain injury in experimental ischemic stroke.

### Microglial *Nr4a1* knockout promotes *Tnf* expression and exacerbates brain injury in experimental ischemic stroke

**Fig 6:**
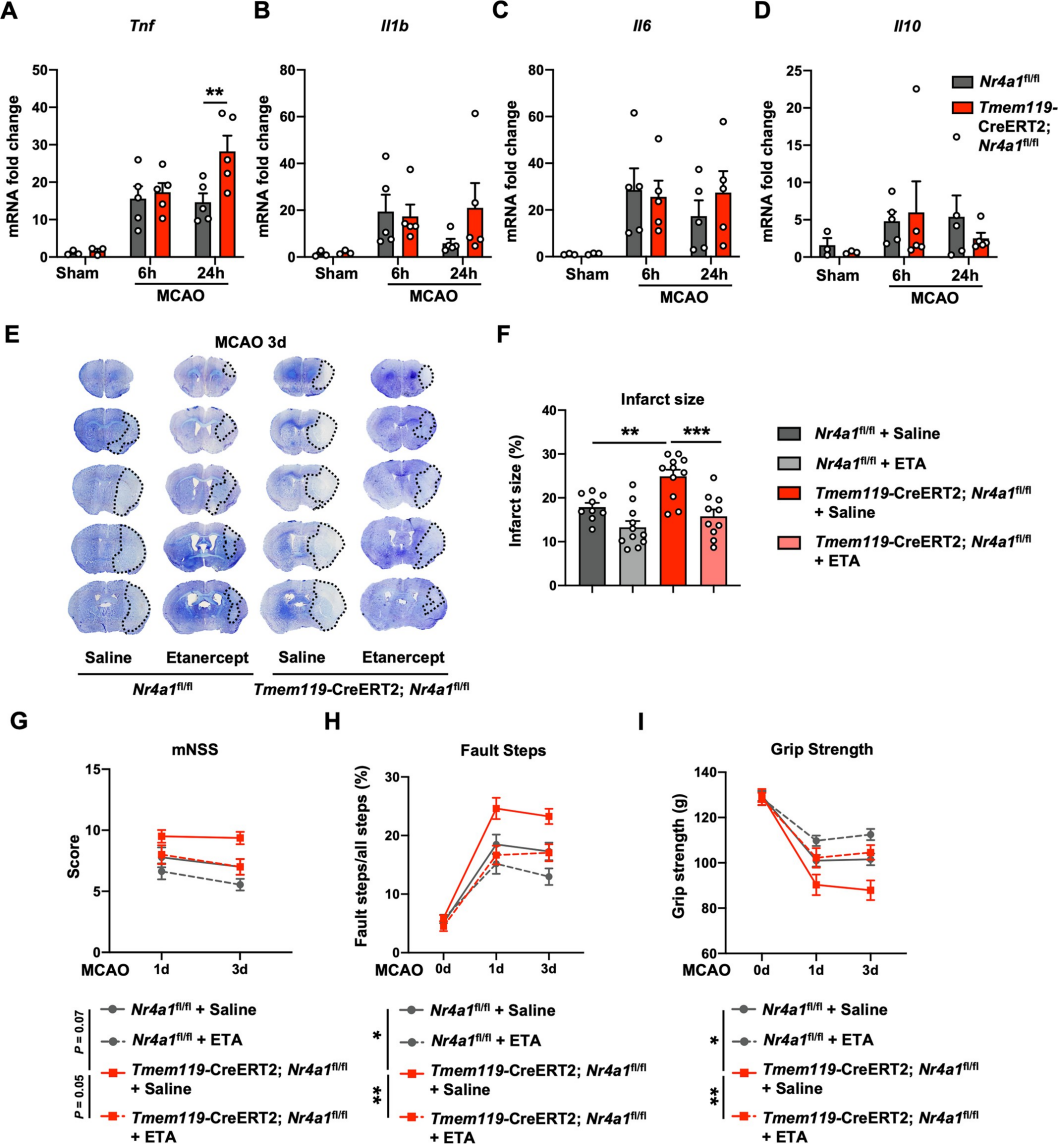
Microglial *Nr4a1* knockout promotes *Tnf* expression and exacerbates brain injury in experimental ischemic stroke. **(A–D)** mRNA levels of several inflammatory factors in ischemic hemispheres from *Tmem119-*CreERT2; *Nr4a1*^fl/fl^ mice and *Nr4a1*^fl/fl^ mice 6 h and 24 h after MCAO (*n* = 3 for sham mice, *n* = 5 for MCAO mice per group). **(E)** Representative images of Nissl-stained tissues from *Tmem119-*CreERT2; *Nr4a1*^fl/fl^ mice and *Nr4a1*^fl/fl^ mice 3 days after MCAO. **(F)** Quantification of the infarct size, as measured by Nissl staining (*n* = 9 for *Nr4a1*^fl/fl^ + Saline mice, *n* = 11 for *Nr4a1*^fl/fl^ + ETA mice, *n* = 11 for *Tmem119-*CreERT2; *Nr4a1*^fl/fl^ + Saline mice, *n* = 10 for *Tmem119-*CreERT2; *Nr4a1*^fl/fl^ + ETA mice). **(G–I)** Neurological deficits were aggravated in *Tmem119-*CreERT2; *Nr4a1*^fl/fl^ mice compared to *Nr4a1*^fl/fl^ mice 1 day and 3 days after MCAO and rescued by ETA, as determined by the mNSS test (**G**) (*n* = 9 for *Nr4a1*^fl/fl^ + Saline mice, *n* = 11 for *Nr4a1*^fl/fl^ + ETA mice, *n* = 11–12 for *Tmem119-*CreERT2; *Nr4a1*^fl/fl^ + Saline mice, *n* = 10 for *Tmem119-*CreERT2; *Nr4a1*^fl/fl^ + ETA mice), foot fault test (**H**) (*n* = 9 for *Nr4a1*^fl/fl^ + Saline mice, *n* = 11 for *Nr4a1*^fl/fl^ + ETA mice, *n* = 10–12 for *Tmem119-*CreERT2; *Nr4a1*^fl/fl^ + Saline mice, *n* = 9–10 for *Tmem119-*CreERT2; *Nr4a1*^fl/fl^ + ETA mice) and grip strength test (**I**) (*n* = 9 for *Nr4a1*^fl/fl^ + Saline mice, *n* = 11 for *Nr4a1*^fl/fl^ + ETA mice, *n* = 11–12 for *Tmem119-*CreERT2; *Nr4a1*^fl/fl^ + Saline mice, *n* = 10 for *Tmem119-*CreERT2; *Nr4a1*^fl/fl^ + ETA mice). Data are presented as mean ± SEM. In (**A–D**), two-way ANOVA with post hoc Bonferroni’s test. In (**F**), one-way ANOVA with post hoc Bonferroni’s test. In (**G–I**), two-way ANOVA with post hoc Bonferroni’s test (day 3 after MCAO) *P < 0.05; **P < 0.01; ***P < 0.001. The underlying data for this figure can be found in S1 Data. MCAO, middle cerebral artery occlusion.

Since we mainly focused on the role of NR4A1 in microglia, conditional microglial *Nr4a1*-knockout mice line (*Tmem119*-CreERT2; *Nr4a1*^*fl/fl*^) was generated and subjected to MCAO (**S4A** and **S4B Fig**). We found that the ischemic hemispheres of *Tmem119-CreERT2; Nr4a1*^*fl/fl*^ mice at 24 h after stroke expressed more *Tnf* mRNA than those from *Nr4a1*^*fl/fl*^ mice, whereas the mRNA expression of *Il1b*, *Il6*, *Il10*, and *Tgfb1* did not show any significant changes (**Figs 6A–6D** and **S5A**). Consistently, the protein level of TNF-α was also up-regulated in *Tmem119*-CreERT2; *Nr4a1*^*fl/fl*^ mice at 24 h after stroke (**S5B–S5F Fig**). Moreover, *Tmem119*-CreERT2; *Nr4a1*^*fl/fl*^ mice phenocopied *Nr4a1*^*-/-*^ mice and showed a larger infarct size and worse neurological deficits than those in *Nr4a1*^*fl/fl*^ littermates after stroke, while Etanercept (ETA, a dimeric Fc-fusion protein that specifically target TNF-α) treatment significantly reduced the infarct size and attenuated neurological deficits in *Tmem119-CreERT2; Nr4a1*^*fl/fl*^ mice, suggesting that increased TNF-α was the major contributing factor that mediate pro-damaging effect of *Nr4a1*-knockout in microglia (**Fig 6E–6I**).

Furthermore, to mimic microglial activation in ischemic stroke in vitro, we induced neuronal injury by incubating neurons in oxygen-glucose deprivation (OGD) conditions and then treated WT and *Nr4a1*^-/-^ primary microglia with neuron-conditioned media (NCM) from control or OGD-exposed neurons (**S6A Fig**). Our results revealed that the expression of *Tnf*, *Il1b*, and *Il6* mRNA was robustly up-regulated and that the levels of anti-inflammatory factors, such as *Arg1* and *Mrc1*, were markedly decreased in *Nr4a1*^-/-^ microglia compared to WT microglia (**S6B Fig**). Consistently, the concentrations of TNF-α, IL-6, and IL-1β were significantly higher in the supernatants of OGD-NCM-treated *Nr4a1*^-/-^ microglia than in the supernatants of OGD-NCM-treated WT microglia (**S6C Fig**). In addition, we also found that culture medium from OGD-NCM-treated *Nr4a1*^-/-^ microglia exacerbated neuronal death compared to that from OGD-NCM-treated WT microglia, as demonstrated by the decrease in the ratio of Calcein-AM^+^/propidium iodide (PI)^+^ cells (**S7A–S7C Fig**). The CCK-8 assay further confirmed that OGD-NCM-treated *Nr4a1*^-/-^ microglia-conditioned medium reduced the viability of neurons (**S7D Fig**). Collectively, these results indicate that microglial NR4A1 suppresses *Tnf* expression and alleviates microglia-potentiated neuronal damage after stroke.

## Discussion

In the present study, we found that NR4A1 was significantly up-regulated in the cytoplasm of activated microglia, which was inconsistent with the role of NR4A1 as a transcription factor, indicating microglial NR4A1 might also function as a non-transcriptional regulator. We then discovered that microglial NR4A1 localized to P-bodies, where it destabilized *Tnf* mRNA in an m^6^A-dependent manner. Global and conditional microglial knockout of *Nr4a1* up-regulated *Tnf* expression and worsened stroke outcomes. Therefore, we uncovered a previously unidentified role of NR4A1 as an RBP, highlighting that microglial NR4A1 is a posttranscriptional brake that suppresses *Tnf* expression.

NR4A1 is widely expressed in the CNS, including the cortex, hippocampus, striatum, and other brain regions [32]. Additionally, it was reported that NR4A1 expression in activated microglia is markedly lower in an MPTP-PD mouse model than that in WT mice, and NR4A1 suppresses deleterious inflammatory responses and protects dopaminergic cells from inflammation-induced death [13]. Rapid up-regulation of NR4A1 in microglia upon exposure to the neuronal-derived stress signal ATP is critical for the maintenance of a resting and noninflammatory phenotype of microglia, which was observed in the EAE model [12].

One outstanding question is the mechanisms underlying NR4A1-mediated anti-inflammatory effect in microglia. It was reported that NR4A1 inhibits the expression of *Tnf* at the transcriptional level after LPS stimulation by suppressing the activation of the NF-κB signaling pathway [33]. Here, we discovered that NR4A1 was significantly up-regulated in the cytoplasm and located in P-bodies in activated microglia, then directly binding *Tnf* mRNA and promoting its destabilization. P-bodies belong to a group of nonmembrane-bound organelles and are assembled by RNAs and RBPs [34]. Posttranscriptional modulation of mRNAs in P-bodies are common mechanisms of mRNA degradation and stabilization [34,35]. DNA-binding proteins (DBPs) and RBPs were considered as 2 distinct categories and studied separately. However, this standpoint has been outdated. Recent studies have found that some DBDs are evolutionarily conserved, which enables them to bind and regulate RNA metabolism [36]. Similar to NR4A1, the glucocorticoid receptor (GCR, also referred to as NR3C1), a classic ligand-activated transcription factor that also has 2 highly conserved Cys_4_ zinc-fingers, was found to destabilize *Ccl2* mRNA through direct RNA binding and the recruitment of UPF1 [37,38]. In addition, P-bodies have also been associated with microRNA (miRNA)-mediated gene silencing, and *Tnf* have been reported to be regulated by multiple miRNAs [39–41]. Therefore, the effect of NR4A1 on miRNA-mediated *Tnf* decay was not fully excluded and needs further investigation.

Notably, TNF-α is a well-characterized proinflammatory cytokine, and increased TNF-α expression is observed in the early phase of stroke in rodent models and postmortem human brain tissues [42–44]. Microglia have been reported to be the dominant source of TNF-α in the brain [45]. TNF-α secreted by microglia can directly act on TNFR1 on neurons and strongly trigger neuronal death by activating caspase-8 signaling after stroke [46,47]. In addition, we previously discovered that TNF-α mediates the interaction between microglia and double-negative T cells after ischemia, leading to aggravated brain injury [48]. Previous studies have identified several RBPs (e.g., TTP, HuR) that target *Tnf* mRNA 3′ UTR to regulate its stability, whose disruption would cause dysregulated TNF-α production and inflammatory diseases [49,50]. Nevertheless, our studies indicated that *Tnf* was significantly affected by NR4A1-deletion in rodent stroke model, while NR4A1 regulates multiple proinflammatory cytokines in in vitro-activated microglia. We speculated that in MCAO mice, NR4A1 was up-regulated in the cytoplasm but absent in the nucleus of microglia; however, in in vitro studies, NR4A1 was significantly induced after microglial activation in both cytoplasm and nucleus, suggesting that *Il1b* and *Il6* were mainly transcriptionally regulated by nuclear NR4A1. We also overexpressed NR4A1-NES in BV2 cells to confirm that cytoplasmic NR4A1 specifically regulate *Tnf* expression, which probably revealed a de novo posttranscriptional role of cytoplasmic NR4A1, therefore expanding the mechanisms underlying its anti-inflammatory role, as well as replenishing the RBP pool that posttranscriptionally regulate the stability of *Tnf* mRNA.

We also found that TSA, a pan-HDAC inhibitor, is effective in increasing NR4A1 expression and down-regulating microglia-derived *Tnf* expression. We proposed that TSA-promoted *Tnf* mRNA degradation in an NR4A1-dependent manner, further supporting our finding that NR4A1 functions as an RBP to posttranscriptionally regulate *Tnf* mRNA in microglia. Several compounds have been found to potentially act as agonists of NR4A1, but the side effects of these compounds limit their application for neurological diseases. Cytosporone B (Csn-B), a natural compound, can suppress microglia-mediated inflammation in an NR4A1-dependent manner, but the specific mechanism underlying the anti-inflammatory effect of Csn-B remains elusive [12]. In addition, since Csn-B has been reported to translocate NR4A1 to mitochondria to induce apoptosis, it might initiate neuronal death, suggesting that its utility for CNS diseases is limited [51]. Celastrol also alleviates inflammation by inducing the interaction between NR4A1 and TRAF2 to promote mitophagy [52]. However, the immunosuppressive effect of celastrol can cause serious infection in patients [53]. Considering the detrimental role of neuroinflammation in multiple sclerosis, Alzheimer’s disease, and Parkinson’s disease, TSA might also be used for treatment of these CNS diseases.

N6-methyladenosine (m^6^A) methylation is the most prevalent modification of eukaryotic mRNAs and is interpreted by its readers to regulate mRNA fate [54,55]. Previous studies have demonstrated that m^6^A writers, erasers, and readers are involved in the immune response under various pathological context. As the most well-identified writer, METTL3 played diverse roles in different diseases. In endotoxemia, METTL3-mediated m^6^A-modification of *Tlr4* showed increased translation and slowed degradation, which was critical for neutrophil activation [56]. METTL3 also enhanced the stabilization of *Tab3* and contributed to the aggravation of renal injury and inflammation [57]. In the spinal cord injury, however, METTL3-methylated *Yap1* mRNA and promoted its stability, leading to reactive astrogliosis and functional recovery [58]. Furthermore, another m^6^A writer, METTL14, was reported to increase the translation of *Foxo1* and aggravated endothelial inflammation and atherosclerosis [24]. ALKBH5, a well-characterized m^6^A eraser, demethylated various target transcripts in neutrophils and promoted neutrophil migration, which is essential for antibacterial defense [59]. As to m^6^A readers, IGF2BP2 could stabilize m^6^A-modified target transcripts (e.g., *Cebpd*, *Tsc1*) to augment autoimmune inflammation [60,61]. However, inactivation of YTHDF2 increased m^6^A-modified inflammation-related transcripts and impaired hematopoietic stem cell function [62]. A previous study also identified m^6^A-hypermethylated mRNAs related to proinflammatory cytokines in the ischemic brain, including *Tnf* mRNA, indicating m^6^A-modification was involved in ischemic stroke [63]. Accordingly, our study identified microglial NR4A1 functions as an RBP to destabilize *Tnf* mRNA in an m^6^A-dependent manner and alleviates post-stroke neuroinflammation, highlighting the role of m^6^A in ischemic stroke.

Notably, a previous study found that m^6^A-loss in *Tnf* mRNA did not directly affect its degradation in macrophages using various approaches [64]. However, there are many m^6^A readers with opposite functions in regulating mRNA stability. For example, IGF2BP proteins could stabilize mRNA, whereas FMR1 was reported to promote the degradation of its target mRNAs [65]. Since the stability of *Tnf* mRNA probably be regulated by both types of m^6^A readers, m^6^A-loss in *Tnf* mRNA might disable all the m^6^A readers to regulate its stability, leading to the net effect of m^6^A-loss on *Tnf* stability to be insignificant. In addition, according to this study, simultaneously knocking down YTHDF proteins significantly down-regulated *Tnf* expression, but the authors did not investigate the effect of YTHDF-knockout on *Tnf* stability. Here, our study used *Nr4a1*-deficient cells rather than *Mettl3/14*-deficient cells to demonstrate that genetic manipulation of specific m^6^A readers could directly regulate *Tnf* stability, which might explain the inconsistency. However, the domain of NR4A1 that binds to m^6^A sites remains elusive. Future structural studies exploring the specific domain that binds to m^6^A-modified mRNAs are warranted. In addition, our study also used BV2 and HEK293T cells instead of primary microglia to demonstrate that NR4A1 regulates *Tnf* by directly binding to the m^6^A sites because these experiments require a large number of cells, and BV2 cells share common features in immune response with primary microglia. Still, the function of NR4A1 as an RBP needs to be further validated in ex vivo microglia (e.g., primary microglia, iPSC-derived microglia).

In addition, NR4A1 was reported to be expressed in various immune cells, including macrophages and T cells [11,66,67]. Since a large number of peripheral immune cells could infiltrate the brain after ischemic stroke, it would be difficult to differ the function of microglial NR4A1 and NR4A1 in other immune cells in *Nr4a1*-knockout mice. Previous studies have confirmed that *Nr4a1*-deficiency caused a significant decrease in Ly6C^-^ monocyte, which might also affect the outcome of ischemic brain injury in mice [68]. In addition, neuronal NR4A1 also protected dopaminergic neurons from MPTP, suggesting the pro-survival role of NR4A1 in neurons [69]. However, in *Tmem119-CreERT2; Nr4a1*^*fl/fl*^ mice, microglial NR4A1 was specifically knockout, enabling us to confirm the neuroprotective role of microglial NR4A1 in the context of ischemic stroke.

Accordingly, our study found that global and conditional microglial knockout of *Nr4a1*-enhanced *Tnf* expression and remarkably exacerbated ischemic brain injury. We also used Etanercept to block the effect of TNF-α in MCAO model and found that Etanercept could rescue the detrimental effect of *Nr4a1*-knockout in microglia. However, the effect of TNF-α in ischemic stroke is controversial. Previous studies using *Tnf*-knockout mice showed that TNF-α was neuroprotective in ischemic stroke, while pharmacologically blocking TNF-α attenuated brain injury [45,70,71]. Moreover, neurons pretreated with TNF-α were more resistant to death [72,73]. It has also been suggested TNF-α was essential for maintaining neuronal function [74]. However, ablation of soluble TNF-α is neuroprotective in ischemic stroke [75]. Thus, we speculated that loss of TNF-α under steady state probably make neurons susceptible to ischemia, while acute blocking increased soluble TNF-α in the injured brain could be neuroprotective. Furthermore, although Etanercept was reported to be neuroprotective in MCAO rats, we found 2 studies reported that Etanercept did not show therapeutic effect in MCAO mice [76,77]. Several differences between our study and theirs might explain the inconsistencies. Sumbria and colleagues [77] used Etanercept at a dose of 1 mg/kg, which is lower than ours (10 mg/kg), indicating that higher dose of Etanercept might exhibit neuroprotective effect. In another study, Etanercept (10 mg/kg) improve neurological function but did not reduce the infarct size of MCAO mice [76]. The reason might be that they used permanent MCAO model, which there is no salvageable penumbra with reperfusion. However, in our study, we used transient MCAO model, and the penumbra exist for at least 24 to 48 h post-stroke in this model [78].

In summary, we found that NR4A1 is critical for accelerating *Tnf* mRNA decay in an m^6^A-dependent manner in P-bodies of activated microglia, which is distinct from its canonical function as a transcription factor. Furthermore, NR4A1 is significantly induced in the cytoplasm of microglia after ischemia and contributes to the suppression of post-stroke *Tnf* up-regulation, leading to improved outcomes. Thus, compounds with the ability to induce NR4A1 expression may improve stroke progression and be potential candidates for stroke therapy.

## Materials and methods

### Ethics statement

All animal procedures were approved by the Animal Care and Use Committee of the Model Animal Research Center, Nanjing University (approval number: 2018010025) and performed according to the Guidelines for the Ethical Review of Laboratory Animal Welfare (GB/T 35892–2018) and the General Requirements for Laboratory Animal Experiments (GB/T 35823–2018) of the People’s Republic of China.

### Animals

*Nr4a1*^-/-^ mice were purchased from Jackson Laboratory. Mice carrying *Nr4a1*-floxed alleles (*Nr4a1*^fl/fl^) were provided by GemPharmatech, and *Tmem119*-CreERT2 mice were provided by Shanghai Model Organisms. Microglia-specific *Nr4a1* conditional knockout (*Tmem119-*CreERT2; *Nr4a1*^*fl/fl*^) mice were generated by breeding *Tmem119-*CreERT2 mice with *Nr4a1*^fl/fl^ mice. Age- and gender-matched *Nr4a1*^fl/fl^ littermates served as WT controls for experiments involving *Tmem119-*CreERT2; *Nr4a1*^*fl/fl*^ mice. *Nr4a1*^-/-^, *Nr4a1*^fl/fl,^ and *Tmem119*-CreERT2; *Nr4a1*^fl/fl^ mice were on the C57BL/6 background. All experimental mice were fed a standard rodent diet and kept on a 12-h light and 12-h dark cycle. For genotyping, 2 primers were used (Cre_1, TGGCCCAGCTCCTCCTCATCCTCT; Cre_2, TCTGGCCTGGTCCCCTTCTGCTCT) for *Tmem119*-CreERT2, which gave 640-bp band; 2 primer pairs were used (Fl _1, AGCCTCTGGTTCTCCACAGA; Fl_2, GGGAAGATCCCAGAACCCAA) for *Nr4a1*-floxed alleles, which gave a 271 bp for WT control and a 360 bp for *Nr4a1*-floxed alleles. *Tmem119-*CreERT2; *Nr4a1*^*fl/fl*^ and *Nr4a1*^*fl/fl*^ mice were intraperitoneally injected with tamoxifen (dissolved in corn oil at 20 mg/ml) (75 mg/kg) for 5 consecutive days to induce Cre activity 3 weeks prior to MCAO.

### Primary mouse cortical neuron culture and neuron-conditioned medium (NCM) preparation

The cortices of E16-18 prenatal embryos were dissected and dissociated by trituration as previously described with minor modification [79]. The dissociated cells were seeded on poly-L-lysine-coated culture plates in neuronal culture medium (MEM supplemented with 5% FBS and 100 μg/ml streptomycin) at a density of 1 × 10^6^ cells/ml, and 24 h after plating, the cells were treated with 25 μm cytosine arabinoside for 18 h to inhibit mitosis. Subsequently, the culture medium was completely changed to neurobasal medium with B27. After a total of 8 to 10 days in culture, the neuronal cells were exposed to OGD conditions. Using MAP2 immunofluorescence staining, we confirmed that >90% of living cells were MAP2+ neurons. OGD was induced and NCM was collected as previously reported. Neurons were initially maintained in an anoxic (95% N2 and 5% CO2) and serum/glucose-free DMEM environment at 37°C for 30 min. The cells were then transferred to a normoxic incubator (95% air and 5% CO2) with normal glucose medium without B27 for reperfusion. The supernatant was harvested after 3 h as NCM, which was used to stimulate primary cultured microglia.

### Primary mouse microglia culture and treatment

Primary microglia were isolated from glial cultures prepared from C57BL/6J and *Nr4a1*^-/-^ neonatal pups (P0-P1) as previously described [80]. Loosely attached microglia were harvested from the medium 10 to 12 days after seeding by shaking the flasks at 300 r.p.m. for 10 min and then plated into 24- or 12-well plates at a density of 5 × 10^5^ cells/ml. The purity of the microglial cells was more than 95%, as determined by Iba1 staining. Forty-eight hours after plating, the microglial cells were washed with PBS twice and then stimulated with 100 ng/ml LPS (Sigma) and 1 mM ATP (Sigma) for 3 h or prepared NCM for 24 h. NCM from control and OGD-treated neurons was centrifuged and diluted to 3:1 with microglial complete medium prior to being added to microglia. After 24 h of treatment with NCM, the medium was completely replaced with fresh microglial medium and collected for downstream experiments. For TSA (HY-15144, MedChemExpress) treatment, primary microglia were pretreated with 50 nM TSA for 2 h prior to ATP and LPS stimulation. For verification of *Nr4a1*-knockout efficiency of primary microglia isolated from *Tmem119-*CreERT2; *Nr4a1*^*fl/fl*^ and *Nr4a1*^*fl/fl*^ mice, seeded microglia were treated with 2 μm 4-hydroxytamoxifen to induce Cre activity.

### Middle cerebral artery occlusion (MCAO)

MCAO was induced by the intraluminal filament method as previously described with some minor improvements [48]. Briefly, 9- to 13-week-old male mice were anesthetized with 1.5% isoflurane in a 30% O_2_/68.5% N_2_O mixture. A 6–0 silicon-coated monofilament nylon suture was inserted into the exposed right external carotid artery and advanced into the internal carotid artery to obstruct the origin of the middle cerebral artery (MCA). The filament was withdrawn to allow cerebral blood reperfusion after 50 min. During the operation, laser Doppler flowmetry (PeriFlux 5000, Perimed) was used to monitor CBF, and a heating lamp was used to maintain the body temperature of the mice at 37°C ± 0.5°C. The suture was considered to have been successfully placed in mice that exhibited a drop in blood perfusion of ≥30% of the baseline level, and mice in which the procedure was unsuccessful were excluded. For Etanercept treatment, mice were intravenously injected with Etanercept (10 mg/kg) or saline immediately after reperfusion and every 24 h for 3 consecutive days.

### Neurological behavior tests

The neurological function of each group of mice was tested after MCAO by using the mNSS test, foot fault test, and grip strength test. The mNSS test consisted of motor, sensory, reflex, and balance tests. Neurological function was graded on a scale of 0 to 18, with 0 representing a normal score and 18 representing the maximal deficit. The rotarod test was performed with a five-lane rotarod device (YLS-4C, Gene&I). The latency to fall off the rotating rod was recorded to measure balance and sensorimotor coordination. The open field test was used to evaluate locomotor activity. Mice were gently placed into a corner of the open field box and allowed to explore freely for 10 min. The total distance moved, mean velocity was recorded. In the foot fault test, mice were placed on an elevated grid made of 12 × 12 mm^2^ cells and allowed to freely explore for 2 min. The behavior of each animal was videotaped. A foot fault was recorded when the left forepaw fell or slipped between the rungs. In the grip strength test, each mouse was held by the tail, lowered so that it could grab the grid of a grip strength meter (BIO-GS3, Bioseb) and then pulled backwards in the horizontal plane. The maximum grip strength of the forelimbs was recorded. The behavioral test data were evaluated by an experimenter blinded to the experiments.

### Quantification of infarct size

Nissl staining was applied to evaluate infarct size 3 days after MCAO. Frozen brain tissues were sectioned into 20-μm thick slices, stained with 1% cresyl violet for 20 min and then differentiated with 70% ethanol for 1 min. The percentage of infarction volume was calculated by the following formula: (mean ipsilateral infarct area × layer thickness)/(mean contralateral hemisphere area × 2 × layer thickness) × 100%.

### RNA isolation and quantitative PCR

Total RNA was extracted from fresh brain tissue and cultured primary microglia using TRIzol (15596026, Thermo Fisher) and reverse-transcribed into cDNA using HiScript III RT SuperMix (R323-01, Vazyme) according to the manufacturer’s instructions. Quantitative real-time PCR was performed on the Applied Biosystems StepOnePlus system with SYBR Green (A25742, Applied Biosystems). Relative gene expression was analyzed by the 2^-(ΔΔCt)^ method, and the values were normalized to the level of *Gapdh*. The primers used are listed in **S3 Table**.

### Preparation of postmortem human brain tissues

The brains of patients who died from ischemic stroke or nonneurological diseases were provided by the Chinese Brain Bank Center (approval number: 2021-scuec-034) (**S4 Table**). Written informed consent was received from the donors before the donation. Unfixed postmortem human brain tissues were processed according to a published protocol with slight modifications [81]. Stroke-affected regions in the cerebral cortex from stroke patients and the corresponding regions from nonstroke patients were dissected and placed into fresh fixative (15% formalin in 0.1 M PBS (pH 7.4)) for 24 to 48 h. These dissected tissue blocks were transferred to 20% sucrose (in 0.1 M PBS with 0.1% Na-azide) for 2 to 3 days followed by 30% sucrose (in 0.1 M PBS with 0.1% Na-azide) for 2 to 3 days and finally embedded in O.C.T. compound (4583, Sakura) for cryosectioning.

### Immunofluorescence staining

Mice were placed under deep anesthesia at different times after MCAO and transcardially perfused with saline followed by cold 4% formaldehyde. After complete infiltration of 15% and 25% sucrose (in 0.1 M PBS), the brain tissues were sectioned into 20-μm slices with a rotatory microtome (Leica). Frozen brain slices and 4% formaldehyde-fixed cultured cells were permeabilized with 0.25% Triton-X100 (in 0.1 M PBS), blocked with 2% BSA (in 0.1 M PBS), and then incubated with primary antibodies against NR4A1 (1:500, NB100-56745, Novus Biologicals; 1:250, sc-166166, Santa Cruz Biotechnology), Iba-1 (1:500, ab5076, Abcam), and DCP1A (1:250, sc-100706, Santa Cruz Biotechnology) at 4°C overnight. After washing, the slices were further incubated with fluorescent dye-conjugated secondary antibodies for 2 h at room temperature. DAPI (1 μg/ml) was used to stain the nuclei. Fluorescence images were obtained using a confocal microscope (FV3000, Olympus).

### Measurement of inflammatory factors

Medium from NCM-treated primary WT and *Nr4a1*^-/-^ microglia was collected, and the concentration of TNF-α, IL-6, IL-12, IFN, MCP-1, and IL-10 were determined by cytometric bead array using a CBA Mouse Inflammation Kit (552364, BD Biosciences). Measurements were taken with a BD Accuri C6 Flow Cytometer, and the data were analyzed using FCAP Array software (BD Biosciences).

Ischemic tissues were homogenized in 1× PBS and stored overnight at −20°C. After 3 freeze–thaw cycle, the homogenates were centrifuged at 14,000 g for 15 min. The supernatant was collected, and the concentration of total protein was quantified by BCA Protein Assay Kit (23227, Thermo Fisher). The levels of TNF-α, IL-1β, IL-6, IL-10, and TGF-β were determined by ELISA kits (TNF-α: CEK1783, Bioworld; IL-1β: CEK1788, Bioworld; IL-6: CEK1785, Bioworld; IL-10: CSB-E04594m, Cusabio; TGF-β: CSB-E04726m, Cusabio).

### Cell viability and apoptosis

To assess the effect of NCM-activated microglia on neuronal activity, primary cortical neurons were incubated with media collected from NCM-treated WT and *Nr4a1*^-/-^ microglia and diluted 1:1 with neuronal culture media for 18 h. Then, calcein-AM/PI staining (C542, Dojindo) and the Cell Counting Kit-8 (CCK-8, CK04, Dojindo) assay were used to detect neuronal survival and death. For Calcein-AM/PI staining, cells were coincubated with 2 mM calcein-AM and 3 mg/ml PI for 15 min at 37°C. Images were captured with a fluorescence microscope (IX73, Olympus). Viability was evaluated and expressed as the ratio of calcein-AM^+^ (green fluorescence) to PI^+^ (red fluorescence) cells. For the CCK8 assay, 10 μl CCK8 was added to the cultured cells for 3.5 h at 37°C in accordance with the manufacturer’s instructions. The optical density (OD) was measured at 450 nm.

### Western blotting

Equal quantities of protein were subjected to SDS-PAGE and transferred to polyvinylidene difluoride (PVDF) membranes (Millipore, United States of America). After being blocked in 5% nonfat milk for 1 h at room temperature, the membranes were incubated overnight at 4°C with primary antibodies against NR4A1 (1:500, sc-166166, Santa Cruz Biotechnology), GAPDH (1:1,000, 5174, Cell Signaling Technology), DCP1A (1:500, sc-100706, Santa Cruz Biotechnology), Histone H3 (1:2,000, 4499, Cell Signaling Technology), YTHDF2 (1:5,000, 24744-1-AP, Proteintech), METTL3 (1:1,000, 15073-1-AP, Proteintech), and FLAG (1:1,000, AE005, Abclonal). The membranes were subsequently incubated with corresponding secondary antibodies and visualized with Western Blotting Chemiluminescent Substrate (WBKLS0500, Millipore).

### RNA electrophoretic mobility shift assay (REMSA)

Biotin-labeled RNA oligonucleotides (biotin-CGUCUCGGACUCGGACUGCU, biotin-CGUCUCGG(m^6^A)CUCGG(m^6^A)CUGCU) were synthesized by Synbio Technologies. One microliter of each RNA probe (2 nM final concentration) and 1 μl of protein (gradient concentration indicated in S3B Fig) were incubated in 8 μl of binding buffer (10 mM HEPES (pH 7.6), 50 mM KCl, 1 mM EDTA, 0.05% Triton X-100, 5% glycerol, 1 mM dithiothreitol, and 40 U/ml RNasin) at room temperature for 30 min. Then, 1 μl of glutaraldehyde (0.2% final concentration) was added to the mixtures and incubated for 10 min at room temperature. The mixtures were then separated on 6% nondenaturing polyacrylamide gels in 0.5× TBE buffer at room temperature for 40 min at 100 V. The RNA oligonucleotides were transferred to nylon membranes at 400 mA for 30 min on ice. Biotin-labeled RNAs were detected by the Chemiluminescent Nucleic Acid Detection Module (89880, Thermo Fisher).

### Protein expression and purification

The DNA sequence of mouse NR4A1 (His-GST-tagged) was subcloned into the pGS21a vector. *E*. *coli* strain BL21 (DE3) was transformed with recombinant plasmid. His-GST-NR4A1 was obtained by two-step purification using a Ni column and Superdex 200. The quality and the purity of the protein were determined by Coomassie staining, and western blotting was performed for confirmation.

### RNA stability assay

Primary microglia were activated with ATP (1 mM) and LPS (100 ng/ml) for 3 h and then treated with actinomycin D (1 μg/ml, A4262, Sigma) to terminate transcription. Total RNA was extracted at the indicated time points and prepared for qPCR.

### Nuclear and cytoplasmic proteins extraction

Nuclear and cytoplasmic proteins were extracted using the NE-PER Nuclear and Cytoplasmic Extraction Reagent Kit (78835, Thermo Fisher) with some modifications. Briefly, 1.0 × 10^7^ cells were centrifuged at 500× g for 2 to 3 min at 4°C. The pellets were resuspended in 100 μl CER I and vigorously vortexed. CER II was then added to the lysates, which were vortexed and centrifuged at 16,000× g for 5 min. The supernatants were collected as the cytoplasmic fractions. The residual pellets were resuspended in NER, repeatedly vortexed, and centrifuged at 16,000× g for 10 min and the supernatants were collected as the nuclear fractions. Nuclear and cytoplasmic proteins were analyzed by western blotting. Histone H3 and GAPDH were used as nuclear and cytoplasmic markers, respectively.

### RNA pull-down

Biotin-labeled RNA oligonucleotides (“GGACU” motif: biotin-CGUCUCGGACUCGGACUGCU, biotin-CGUCUCGG(m^6^A)CUCGG(m^6^A)CUGCU; “AGACA” motif: biotin-CGUCUCAGACACAGACAGCU, biotin-CGUCUCAG(m^6^A)CACAG(m^6^A)CAGCU) were synthesized by Synbio Technologies. RNA probes (50 pmol) were conjugated to 30 μl streptavidin magnetic beads (HY-K0208, MedChemExpress) in binding buffer (10 mM HEPES (pH 7.6), 50 mM KCl, 1 mM EDTA, 0.05% Triton X-100, 5% glycerol, 1 mM dithiothreitol, and 40 U/ml RNasin) at 4°C for 30 min. RNA probe-conjugated magnetic beads were then incubated with 250 μg cytoplasmic extract from ATP (1 mM) and LPS (100 ng/ml)-activated primary microglia in binding buffer in a final volume of 1 ml for 3 h at 4°C. After repeated washes, the beads were resuspended in 1× western blot loading buffer and boiled at 98°C for 5 min to elute the protein from the beads for western blot analysis.

### RNA-binding protein immunoprecipitation (RIP)

RIP was performed following the instructions of the Magna RIP Kit (17–700, Millipore) with some modifications. Briefly, RIP lysates from 2.0 × 10^7^ BV2 cells or 1.0 × 10^7^ microglia were collected in RIP lysis buffer. Five micrograms of an anti-NR4A1 (sc-166166, Santa Cruz Biotechnology) antibody or control mouse IgG (CS200621, Millipore) were immobilized onto protein A/G magnetic beads by incubation in RIP Wash Buffer for 1 h at room temperature. After 3 washes, the beads were incubated with the RIP lysates in RIP Immunoprecipitation Buffer overnight at 4°C. After 5 washes with RIP Wash Buffer, the beads were incubated in Proteinase K Buffer at 55°C for 30 min to digest the protein. RNA was extracted by phenol: chloroform: isoamyl alcohol (125:24:1) and analyzed by qPCR.

### m^6^A-RIP-qPCR

m^6^A-RIP was performed according to a published protocol with slight modifications [82]. Briefly, 300 μg total RNA was fragmented into approximately 100-nucleotide oligonucleotides by meta-induced fragmentation. Then, the purified RNA fragments were incubated with 12.5 μg m^6^A-specific antibody (202003, Synaptic Systems) or rabbit IgG (PP64B, Millipore) in immunoprecipitation buffer (50 mM Tris-HCl, 750 mM NaCl, and 0.5% NP-40) supplemented with RNase (0.4 U/μl) and ribonucleoside vanadyl complexes (2 mM) for 2 h at 4°C. m^6^A-RNA was immunoprecipitated with magnetic beads and eluted by competition with free m^6^A (M2780, Sigma-Aldrich) in elution buffer (50 mM Tris-HCl, 750 mM NaCl, 0.5% NP-40, and 6.7 mM m^6^A). Enrichment of m^6^A was analyzed by qPCR. The primers used for m^6^A-RIP-qPCR are listed in **S3 Table**.

### Dual-luciferase reporter assay

The 5′ UTR, CDS, and 3′ UTR of *Tnf* were cloned into the pGL3-promoter vector (Promega) at the HindIII, NcoI, XbaI, and FseI restriction sites. Mutants were generated by site-directed mutagenesis. Mouse *Tnf* promoter(−1,260 + 140 bp) was cloned into pGL3-Basic vector at the MluI and XhoI restriction sites. A luciferase reporter plasmid (1.5 μg) and TK-Renilla (0.16 μg) were cotransfected into HEK293T cells in 12-well plates using Lipofectamine 3000 (L3000015, Invitrogen). After 24 h, firefly/Renilla luciferase activities were measured using a Promega Dual-Luciferase reporter system (E2920, Promega).

### Proximity ligation assay

Protein interactions were detected by the proximity ligation assay using the Duolink in Situ Fluorescence Kit (DUO92008-30RXN, Sigma). Briefly, primary microglia were fixed in 4% paraformaldehyde at room temperature for 15 min, permeabilized in 0.25% Triton X-100 at room temperature for 20 min, and blocked in the provided blocking buffer for 1 h at 37°C. The cells were then incubated with primary antibodies (anti-NR4A1 and anti-DCP1A antibody) overnight at 4°C. Subsequent secondary antibody (anti-rabbit PLUS and anti-mouse MINUS) incubation, ligation, and amplification were performed according to the manufacturer’s instructions.

### NR4A1 overexpression in BV2 cells

BV2 cells were transfected with lentivirus overexpressing FLAG-NR4A1 (Shanghai Genechem) or FLAG-NR4A1-NES (Shanghai Genechem) and allowed to grow for 72 h for subsequent experiments.

### CRISPR/Cas9-mediated *Mettl3* knockout in BV2 cells

BV2 cells were transfected with a Cas9 lentivirus containing *Mettl3* single-guide RNAs (sgRNA) (Shanghai Genechem). Single cells were isolated 72 h after transfection into 96-well plates. Independent clones were allowed to grow for 3 weeks. The sgRNA sequence was GTTGGGGACAGTGCCGCTTC.

### Dot blot

The indicated amount of RNA oligonucleotides was incubated at 95°C for 3 min, followed by chilling on ice. RNA samples were dropped onto nylon membranes. After UV crosslinking, the membrane was stained with 0.02% methylene blue in 0.3 M sodium acetate. The membrane was then blocked with 5% nonfat milk and incubated with anti-m^6^A antibody (1:1,000, 202003, Synaptic Systems) overnight at 4°C. After incubating with secondary antibody, the membrane was visualized with Western Blotting Chemiluminescent Substrate (WBKLS0500, Millipore).

### Photoactivatable ribonucleotide crosslinking and immunoprecipitation (PAR-CLIP)

PAR-CLIP was performed according to a published protocol with slight modifications [83,84]. Briefly, BV2 cells were treated with 4-thiouridine (4-SU) (100 μM) for 16 h and then exposed to UVA (365 nm) for crosslinking. Next, the cells were resuspended in NP-40 lysis buffer and incubated with RNase T1 (1 U/μl) at 22°C for 10 min. Five micrograms of an anti-NR4A1 (sc-166166, Santa Cruz Biotechnology) antibody or control mouse IgG (CS200621, Millipore) were immobilized onto protein A/G magnetic beads. The cell lysates were then incubated with the beads in RIP Immunoprecipitation Buffer overnight at 4°C. After 5 washes with RIP Wash Buffer, the beads were incubated in Proteinase K Buffer at 55°C for 30 min to digest the protein. RNA was extracted by phenol: chloroform: isoamyl alcohol (125:24:1) and analyzed by qPCR.

### Protein coimmunoprecipitation (Co-IP)

BV2 cells were grown in 10-cm dishes, transfected with lentivirus overexpressing FLAG-NR4A1 (Shanghai Genechem), and lysed with lysis buffer (25 mM Tris-HCl (pH 7.4), 150 mM NaCl, 1 mM EDTA, 1% NP40, and 5% glycerol) for 15 min and then centrifuged at 12,000 r.p.m. for 15 min to collect clear lysates. For immunoprecipitation, cell lysates were incubated with 1 μg FLAG antibody (14793, Cell Signaling Technology) and rabbit IgG (PP64B, Millipore) at 4°C overnight and then incubated with Protein G Agarose Beads (Millipore) at 4°C for 2 h. After incubation, the beads were washed 5 times with lysis buffer. The precipitated proteins were eluted with 1× western blot loading buffer and analyzed by western blotting.

### Coimmunoprecipitation (Co-IP) coupled with mass spectrometry (MS)

Precipitated protein samples were subjected to SDS-PAGE and visualized by Coomassie Blue staining. MS was performed by Jingjie PTM Biolabs. Protein-containing gel pieces were destained with 50 mM NH_4_HCO_3_ in 50% acetonitrile, dehydrated with 100 μl 100% acetonitrile for 5 min, rehydrated in 10 mM dithiothreitol, and then incubated at 56°C for 60 min. After sequential dehydration (with 100% acetonitrile) and rehydration (with 55 mM iodoacetamide), the gel pieces were incubated at room temperature for 45 min, washed with 50 mM NH_4_HCO_3_, dehydrated again with 100% acetonitrile, rehydrated with 10 ng/μl trypsin solution (in 50 mM NH_4_HCO_3_) on ice for 1 h, and digested with trypsin at 37°C overnight. The tryptic peptides were extracted with 50% acetonitrile/5% formic acid. The tryptic peptides were dissolved in 0.1% formic acid (solvent A) and directly loaded onto a reversed-phase analytical column (15 cm in length, 75-μm i.d.), with a gradient of solvent B (0.1% formic acid in 98% acetonitrile) increasing from 6% to 23% over 16 min at a constant flow rate of 400 nl/min on an EASY-nLC 1000 UPLC system. The peptides were subjected to an NSI source and quantified by MS/MS on a Q Exactive Plus mass spectrometer (Thermo Fisher) coupled online to the UPLC system using an electrospray voltage of 2.0 kV. Full scan spectra from m/z 350 to 1,800 at resolution of 70,000 were acquired in the Orbitrap. Peptides were then selected for MS/MS at an NCE of 28, and the fragments were detected in the Orbitrap at a resolution of 17,500. The data-dependent procedure that alternated between 1 MS scan followed by 20 MS/MS scans with 15.0 s dynamic exclusion. Automatic gain control (AGC) was set at 5E4. The resulting MS/MS data were processed using Proteome Discoverer 1.3. Tandem mass spectra were searched against the UniProtKB mouse database. Trypsin/P was specified as a cleavage enzyme. The mass error was set to 10 ppm for precursor ions and 0.02 Da for fragment ions. Carbamidomethylation of Cys was specified as a fixed modification, and oxidation of Met was specified as a variable modification. Peptide confidence was set to high, and the peptide ion score was set to >20. The GO analysis was performed by the OmicShare tools (http://omicshare.com/tools).

### RIP-seq analysis

NR4A1-binding RNAs were precipitated from 5.0 × 10^7^ ATP+LPS-stimulated BV2 cells with anti-NR4A1 (sc-166166, Santa Cruz Biotechnology) antibody, and rRNAs were then removed using Ribo-off rRNA Depletion Kit (N406-01/02, Vazyme) according to the manufacturer’s instruction. The purified RNAs were used to construct the library by Novogene Corporation. Subsequently, pair-end sequencing of sample was performed on Illumina platform. Library quality was assessed on the Agilent Bioanalyzer 2100 system. Peaks of RIP enrichment over background were identified using a peak calling R package, RIPSeeker [85]. HOMER was used to detect the motifs in peak regions.

### Statistical analysis

The data are represented as mean ± SEM. Statistical differences were determined by one-way ANOVA followed by post hoc multiple-comparisons tests (Dunnett’s correction) to analyze differences among 3 or more groups with 1 independent variable, by two-way ANOVA followed by post hoc multiple-comparisons tests (Bonferroni’s correction) to analyze among 3 or more groups with 2 independent variables or by unpaired Student’s *t* test to analyze differences between 2 groups. *P* < 0.05 was considered statistically significant.

## Supporting information

S1 Fig ATP and ATP+LPS induces different expression pattern of NR4A1 in microglia. **(A)** Immunoblot analysis of NR4A1 in primary microglia treated with or without LPS (100 ng/ml), ATP (1mM), or ATP (1 mM) + LPS (100 ng/ml). The original blot for this figure can be found in S1 Raw Images.
(TIF)

S2 Fig Cytoplasmic NR4A1 specifically regulates *Tnf* mRNA. **(A–C)** RIP analysis of NR4A1-bound *Il1b*, *Il6*, *Il10* mRNA in untreated and ATP+LPS-treated BV2 cells (*n* = 3 biological repeats in each group). **(D–F)** RNA stability assay of *Il1b*, *Il6*, *Il10* mRNA in ATP+LPS-activated WT and *Nr4a1*^-/-^ primary microglia at the indicated time points after actinomycin D treatment (*n* = 4 biological repeats in each group). **(G)** Luciferase activities of the promoter of *Tnf* in HEK293T cells overexpressing NR4A1 or empty vector (*n* = 4 biological repeats in each group). **(H)** Immunoblot analysis of NR4A1 overexpression in BV2 cells treated with or without ATP+LPS. **(I)** Immunoblot analysis of NR4A1-NES overexpression in BV2 cells treated with or without ATP+LPS. **(J)** Representative images of BV2 cells with FLAG-NR4A1 or FLAG-NR4A1-NES overexpression showing subcellular localization of FLAG-NR4A1 and FLAG-NR4A1-NES. Scale bar, 30 μm. **(K)** mRNA levels of *Tnf*, *Il1b*, and *Il6* in BV2 cells with or without WT-NR4A1 overexpression treated with or without ATP+LPS (*n* = 3 biological repeats in each group). **(L)** mRNA levels of *Tnf*, *Il1b*, and *Il6* in BV2 cells with or without NR4A1-NES overexpression treated with or without ATP+LPS (*n* = 3 biological repeats in each group). **(M)** RNA stability assay of *Tnf* mRNA in ATP+ LPS-activated BV2 cells with or without NR4A1-NES overexpression at the indicated time points after actinomycin D treatment (*n* = 3 biological repeats in each group). Data are presented as mean ± SEM. In (**A**), (**B**), (**C**), (**K**), (**L**) two-way ANOVA with post hoc Bonferroni’s test. In (**G**), two-tailed unpaired Student’s *t* test. In (**D**), (**E**), (**F**), (**M**) two-way ANOVA. *P < 0.05; **P < 0.01; ***P < 0.001. The underlying data for this figure can be found in S1 Data. The original blot for this figure can be found in S1 Raw Images.
(TIF)

S3 Fig Cytoplasmic NR4A1 regulates *Tnf* mRNA in an m^6^A-dependent manner. **(A)** Dot blot of m^6^A-containing or unmethylated oligonucleotides at the indicated concentrations. **(B)** REMSA of purified recombinant NR4A1 at the indicated concentrations to m^6^A-containing or unmethylated oligonucleotides. **(C)** PAR-CLIP analysis of NR4A1-bound m^6^A-containing CDS and 3′ UTR region of *Tnf* mRNA in ATP+LPS-treated BV2 cells (*n* = 3 biological repeats in each group). **(D)** Immunoblot analysis of METTL3 expression in BV2 cells with or without *Mettl3*-KO. **(E)** mRNA levels of *Tnf* in *Mettl3*-KO BV2 cells with or without ATP+LPS treatment (*n* = 3 biological repeats in each group). Data are presented as mean ± SEM. In (**C**), multiple Student’s *t* test. In (**F**), two-way ANOVA with post hoc Bonferroni’s test. *P < 0.05. The underlying data for this figure can be found in S1 Data. The original blot for this figure can be found in S1 Raw Images.
(TIF)

S4 Fig Validation of microglia-specific knockout of *Nr4a1*. **(A)** Genotyping of *Tmem119*-CreERT2; *Nr4a1*^fl/fl^ mice. **(B)** Immunoblot analysis of NR4A1 expression in primary microglia isolated from *Nr4a1*^fl/fl^ and *Tmem119*-CreERT2; *Nr4a1*^fl/fl^ mice treated with 4-Hydroxytamoxifen (2 μm) with or without ATP+LPS stimulation. The original blot for this figure can be found in S1 Raw Images.
(TIF)

S5 Fig Microglial *Nr4a1* knockout promotes TNF-α expression. **(A)** mRNA level of *Tgfb1* in ischemic hemispheres from *Tmem119-*CreERT2; *Nr4a1*^fl/fl^ mice and *Nr4a1*^fl/fl^ mice 6h and 24h after MCAO (*n* = 3 for sham mice, *n* = 5 for MCAO mice per group). **(B–F)** Protein levels of several inflammatory factors in ischemic hemispheres from *Tmem119-*CreERT2; *Nr4a1*^fl/fl^ mice and *Nr4a1*^fl/fl^ mice 6 h and 24 h after MCAO (*n* = 3 for sham mice, *n* = 5 for MCAO mice per group). Data are presented as mean ± SEM. In (**A–F**), two-way ANOVA with post hoc Bonferroni’s test. *P < 0.05. The underlying data for this figure can be found in S1 Data.
(TIF)

S6 Fig NR4A1 alleviates microglia-mediated neuroinflammation in in vitro stroke model. **(A)** Paradigm of the preparation of oxygen-glucose deprivation-treated neuron-conditioned media (OGD-NCM). **(B, C)** mRNA (**B**) and protein (**C**) levels of several inflammatory factors in WT and *Nr4a1*^-/-^ primary microglia exposed to conditioned medium from control or OGD-treated neurons (in **B**, *n* = 6 biological repeats for CON-NCM-treated WT and *Nr4a1*^-/-^ primary microglia, *n* = 8 biological repeats for OGD-NCM-treated WT and *Nr4a1*^-/-^ primary microglia; in **C**, *n* = 3 biological repeats in each group). Data are presented as mean ± SEM. In (**B**), (**C**), two-way ANOVA with post hoc Bonferroni’s test. *P < 0.05; **P < 0.01; ***P < 0.001. The underlying data for this figure can be found in S1 Data.
(TIF)

S7 Fig NR4A1 alleviates microglia-potentiated neuronal damage in in vitro stroke model. **(A)** Paradigm of the preparation of oxygen-glucose deprivation-treated neuron-conditioned media (OGD-NCM) and microglia-conditioned media (MCM). **(B)** Representative images of Calcein-AM/PI-stained primary neurons treated with conditioned medium from activated WT or *Nr4a1*^-/-^ primary microglia. Scale bar, 20 μm. **(C)** Quantification of Calcein-AM/PI staining (*n* = 4, 4 and 6 biological repeats for control, WT MEM and *Nr4a1*^-/-^ MEM). **(D)** Results of the CCK8 assay in primary neurons treated with conditioned medium from activated WT or *Nr4a1*^-/-^ primary microglia (*n* = 4, 4 and 5 biological repeats for control, WT and *Nr4a1*^-/-^ MEM). Data are presented as mean ± SEM. In (**C**), (**D**), one-way ANOVA with post hoc Dunnett’s test. **P < 0.01; ***P < 0.001. The underlying data for this figure can be found in S1 Data.
(TIF)

S1 Table NR4A1-interacting proteins. (DOCX)

S2 Table NR4A1-binding peaks (RIP-seq). (XLS)

S3 Table Primers used in this study. (DOCX)

S4 Table Information of donors. (DOCX)

S1 Data Underlying data for Figs 1H; 2A–C, 2E–H; 3F–3G, 3I–3J; 4B and 4D; Fig 5A–5D, 5F–5J; 6A–6D, 6F–6I; S2A–S2G, S2K–S2M; S3C and S3E; S5A–S5F; S6B and S6C, S7C and S7D.
(XLSX)

S1 Raw Images Original images supporting blot and gel results for Figs 1A and 1B, 1I; 2D; 3D; 4A; S1; S2H and S2I; S3A, S3B, and S3D; S4A and S4B.
(PDF)

## Abbreviations

AGC: automatic gain control
Co-IP: coimmunoprecipitation
DAMP: damage-associated molecular pattern
DBD: DNA-binding domain
DBP: DNA-binding protein
EAE: experimental autoimmune encephalomyelitis
GCR: glucocorticoid receptor
LBD: ligand-binding domain
MCA: middle cerebral artery
MCAO: middle cerebral artery occlusion
mNSS: modified neurological severity score
miRNA: microRNA
NCM: neuron-conditioned media
OD: optical density
OGD: oxygen-glucose deprivation
P-bodies: processing bodies
PI: propidium iodide
RBP: RNA-binding protein
REMSA: RNA electrophoretic mobility shift assay
RIP-seq: RNA-binding protein immunoprecipitation sequencing
TSA: trichostatin A
WT: wild-type
