# Supplementary figures and images for: Noncanonical contribution of microglial transcription factor NR4A1 to post-stroke recovery through TNF mRNA destabilization
Source: PLoS Biol. 2023 Jul 24;21(7):e3002199. doi: 10.1371/journal.pbio.3002199 (PMC10365314; doi:10.1371/journal.pbio.3002199)

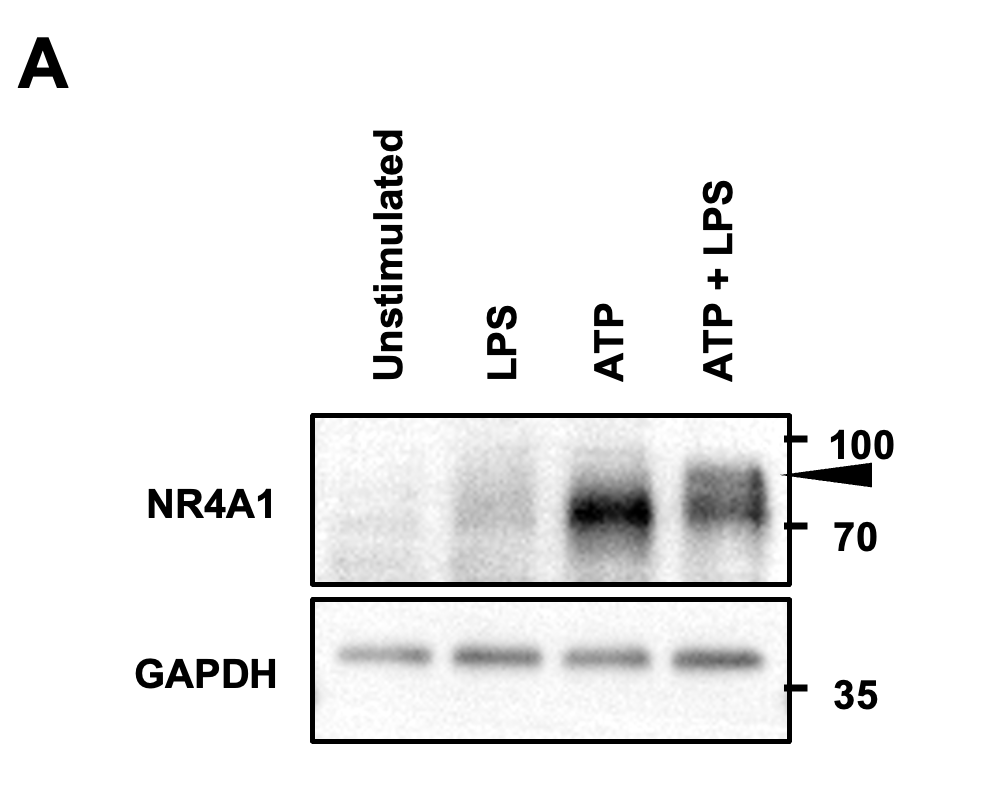

Supplement: S1 Fig — (A) Immunoblot analysis of NR4A1 in primary microglia treated with or without LPS (100 ng/ml), ATP (1mM), or ATP (1 mM) + LPS (100 ng/ml). The original blot for this figure can be found in S1 Raw Images. (TIF) [file pbio.3002199.s001.tif]

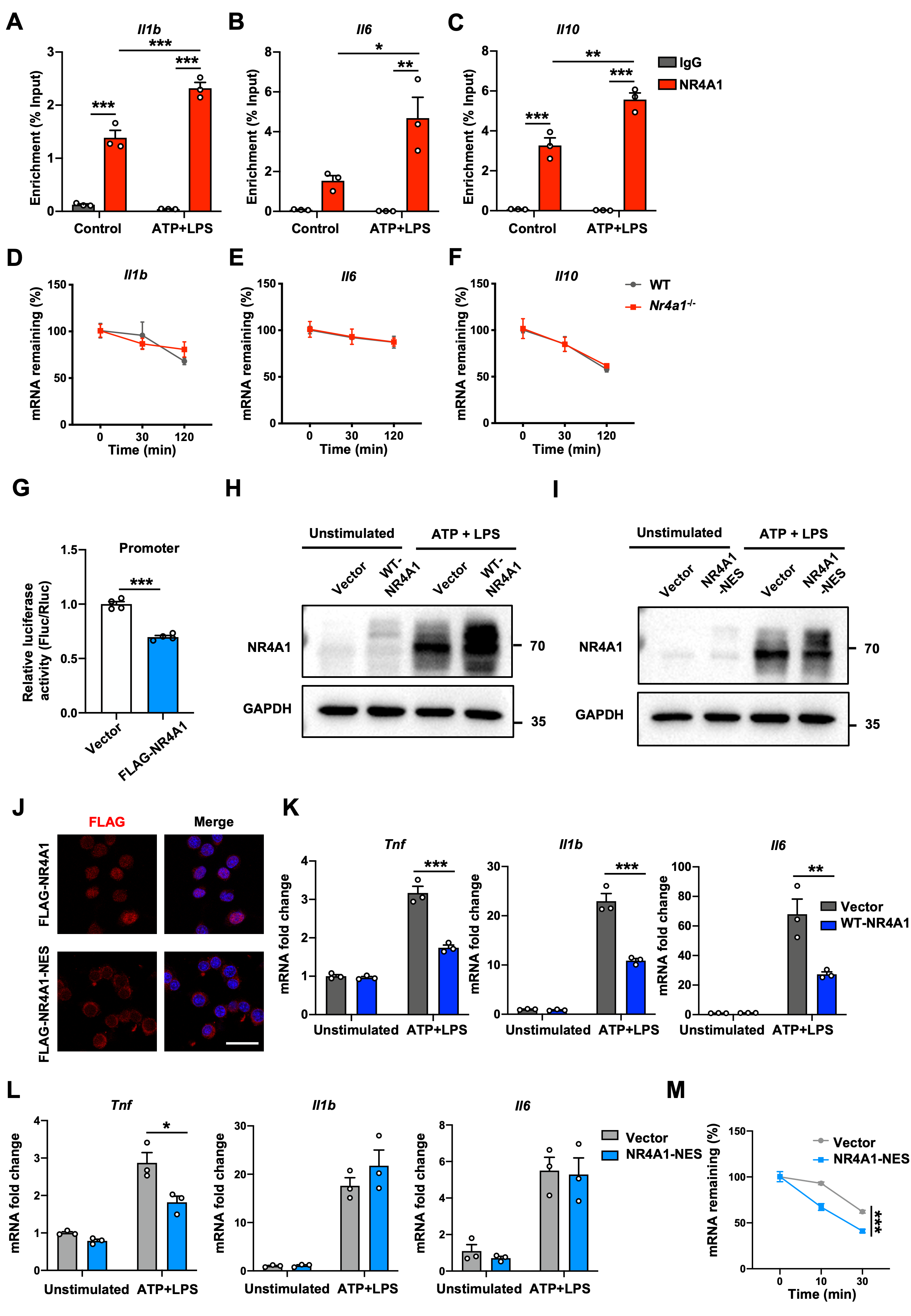

Supplement: S2 Fig — (A–C) RIP analysis of NR4A1-bound Il1b, Il6, Il10 mRNA in untreated and ATP+LPS-treated BV2 cells (n = 3 biological repeats in each group). (D–F) RNA stability assay of Il1b, Il6, Il10 mRNA in ATP+LPS-activated WT and Nr4a1-/- primary microglia at the indicated time points after actinomycin D treatment (n = 4 biological repeats in each group). (G) Luciferase activities of the promoter of Tnf in HEK293T cells overexpressing NR4A1 or empty vector (n = 4 biological repeats in each group). (H) Immunoblot analysis of NR4A1 overexpression in BV2 cells treated with or without ATP+LPS. (I) Immunoblot analysis of NR4A1-NES overexpression in BV2 cells treated with or without ATP+LPS. (J) Representative images of BV2 cells with FLAG-NR4A1 or FLAG-NR4A1-NES overexpression showing subcellular localization of FLAG-NR4A1 and FLAG-NR4A1-NES. Scale bar, 30 μm. (K) mRNA levels of Tnf, Il1b, and Il6 in BV2 cells with or without WT-NR4A1 overexpression treated with or without ATP+LPS (n = 3 biological repeats in each group). (L) mRNA levels of Tnf, Il1b, and Il6 in BV2 cells with or without NR4A1-NES overexpression treated with or without ATP+LPS (n = 3 biological repeats in each group). (M) RNA stability assay of Tnf mRNA in ATP+ LPS-activated BV2 cells with or without NR4A1-NES overexpression at the indicated time points after actinomycin D treatment (n = 3 biological repeats in each group). Data are presented as mean ± SEM. In (A), (B), (C), (K), (L) two-way ANOVA with post hoc Bonferroni’s test. In (G), two-tailed unpaired Student’s t test. In (D), (E), (F), (M) two-way ANOVA. *P < 0.05; **P < 0.01; ***P < 0.001. The underlying data for this figure can be found in S1 Data. The original blot for this figure can be found in S1 Raw Images. (TIF) [file pbio.3002199.s002.tif]

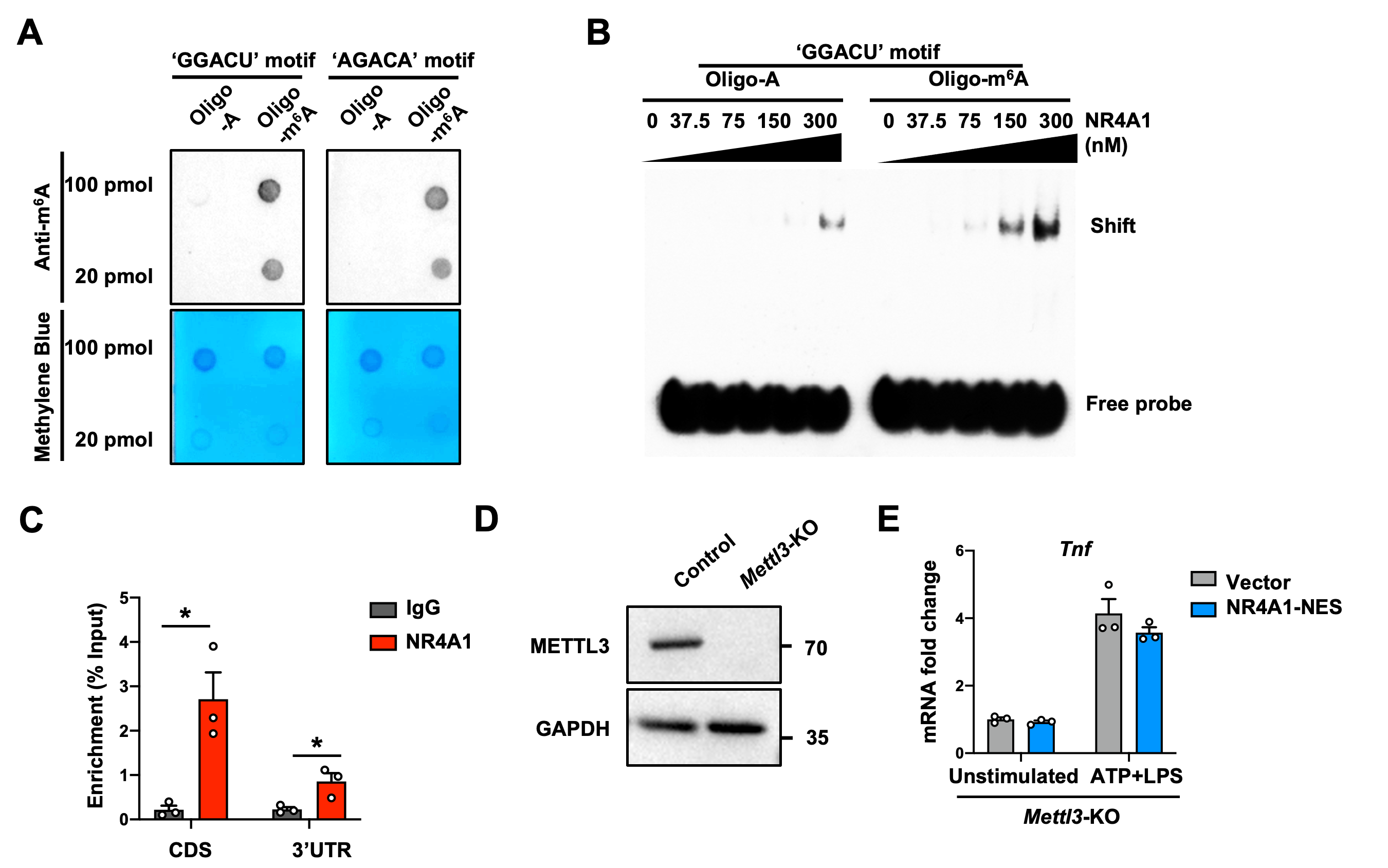

Supplement: S3 Fig — (A) Dot blot of m6A-containing or unmethylated oligonucleotides at the indicated concentrations. (B) REMSA of purified recombinant NR4A1 at the indicated concentrations to m6A-containing or unmethylated oligonucleotides. (C) PAR-CLIP analysis of NR4A1-bound m6A-containing CDS and 3′ UTR region of Tnf mRNA in ATP+LPS-treated BV2 cells (n = 3 biological repeats in each group). (D) Immunoblot analysis of METTL3 expression in BV2 cells with or without Mettl3-KO. (E) mRNA levels of Tnf in Mettl3-KO BV2 cells with or without ATP+LPS treatment (n = 3 biological repeats in each group). Data are presented as mean ± SEM. In (C), multiple Student’s t test. In (F), two-way ANOVA with post hoc Bonferroni’s test. *P < 0.05. The underlying data for this figure can be found in S1 Data. The original blot for this figure can be found in S1 Raw Images. (TIF) [file pbio.3002199.s003.tif]

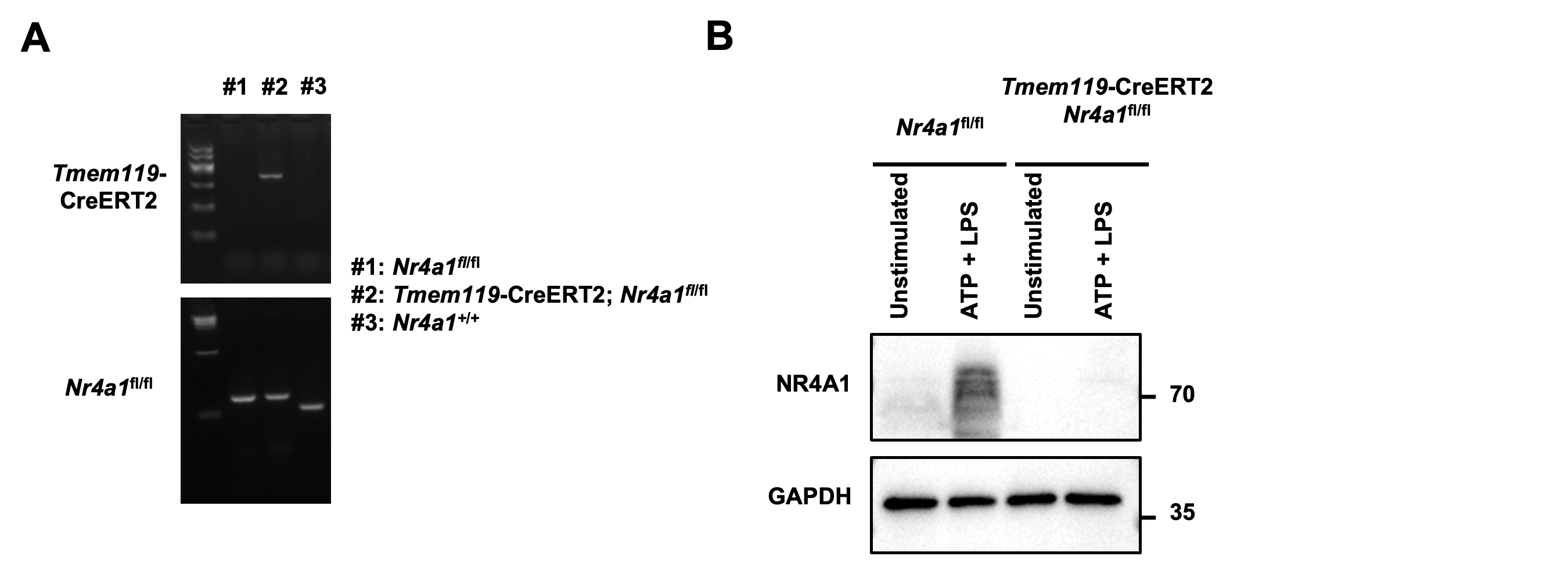

Supplement: S4 Fig — (A) Genotyping of Tmem119-CreERT2; Nr4a1fl/fl mice. (B) Immunoblot analysis of NR4A1 expression in primary microglia isolated from Nr4a1fl/fl and Tmem119-CreERT2; Nr4a1fl/fl mice treated with 4-Hydroxytamoxifen (2 μm) with or without ATP+LPS stimulation. The original blot for this figure can be found in S1 Raw Images. (TIF) [file pbio.3002199.s004.tif]

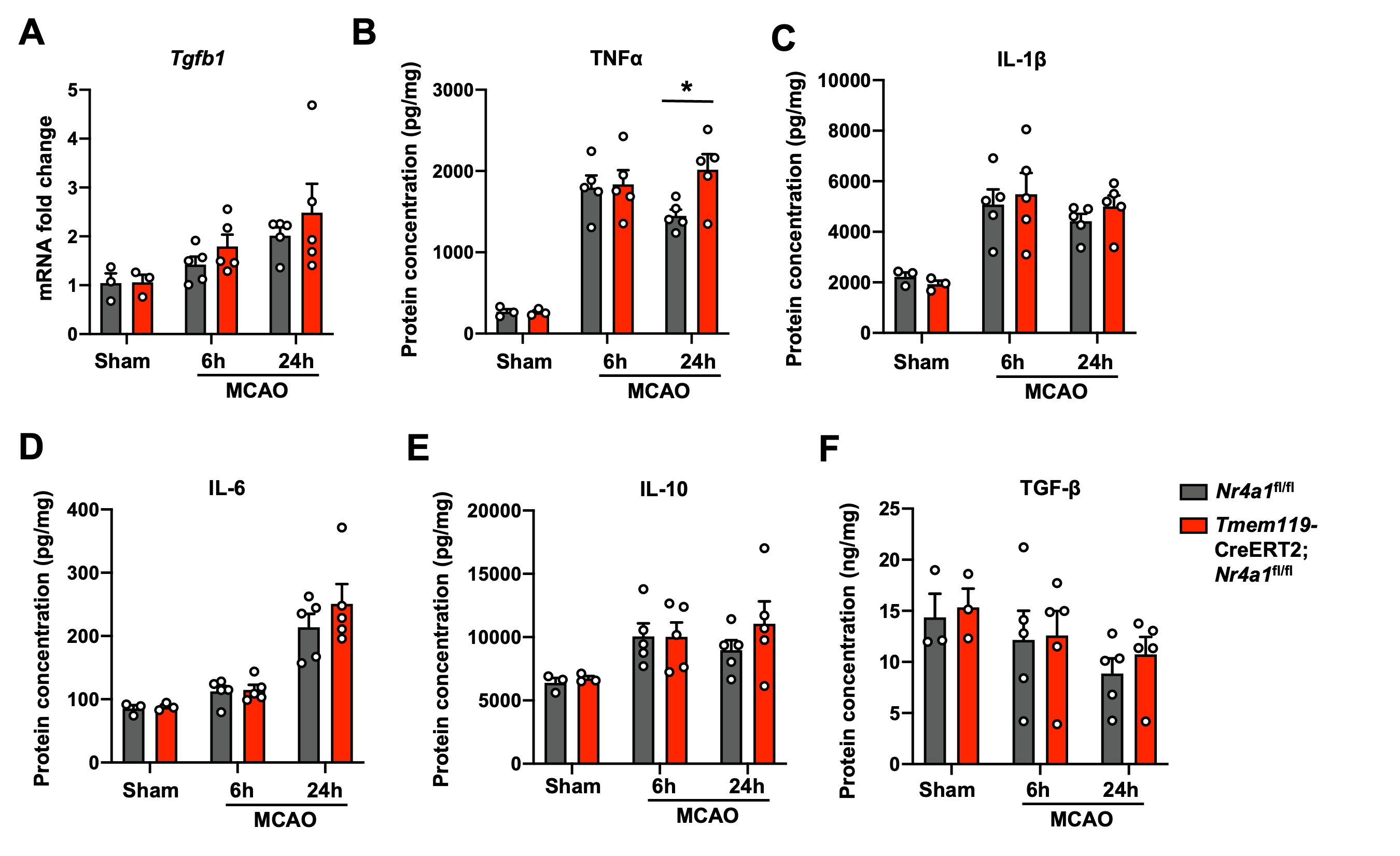

Supplement: S5 Fig — (A) mRNA level of Tgfb1 in ischemic hemispheres from Tmem119-CreERT2; Nr4a1fl/fl mice and Nr4a1fl/fl mice 6h and 24h after MCAO (n = 3 for sham mice, n = 5 for MCAO mice per group). (B–F) Protein levels of several inflammatory factors in ischemic hemispheres from Tmem119-CreERT2; Nr4a1fl/fl mice and Nr4a1fl/fl mice 6 h and 24 h after MCAO (n = 3 for sham mice, n = 5 for MCAO mice per group). Data are presented as mean ± SEM. In (A–F), two-way ANOVA with post hoc Bonferroni’s test. *P < 0.05. The underlying data for this figure can be found in S1 Data. (TIF) [file pbio.3002199.s005.tif]

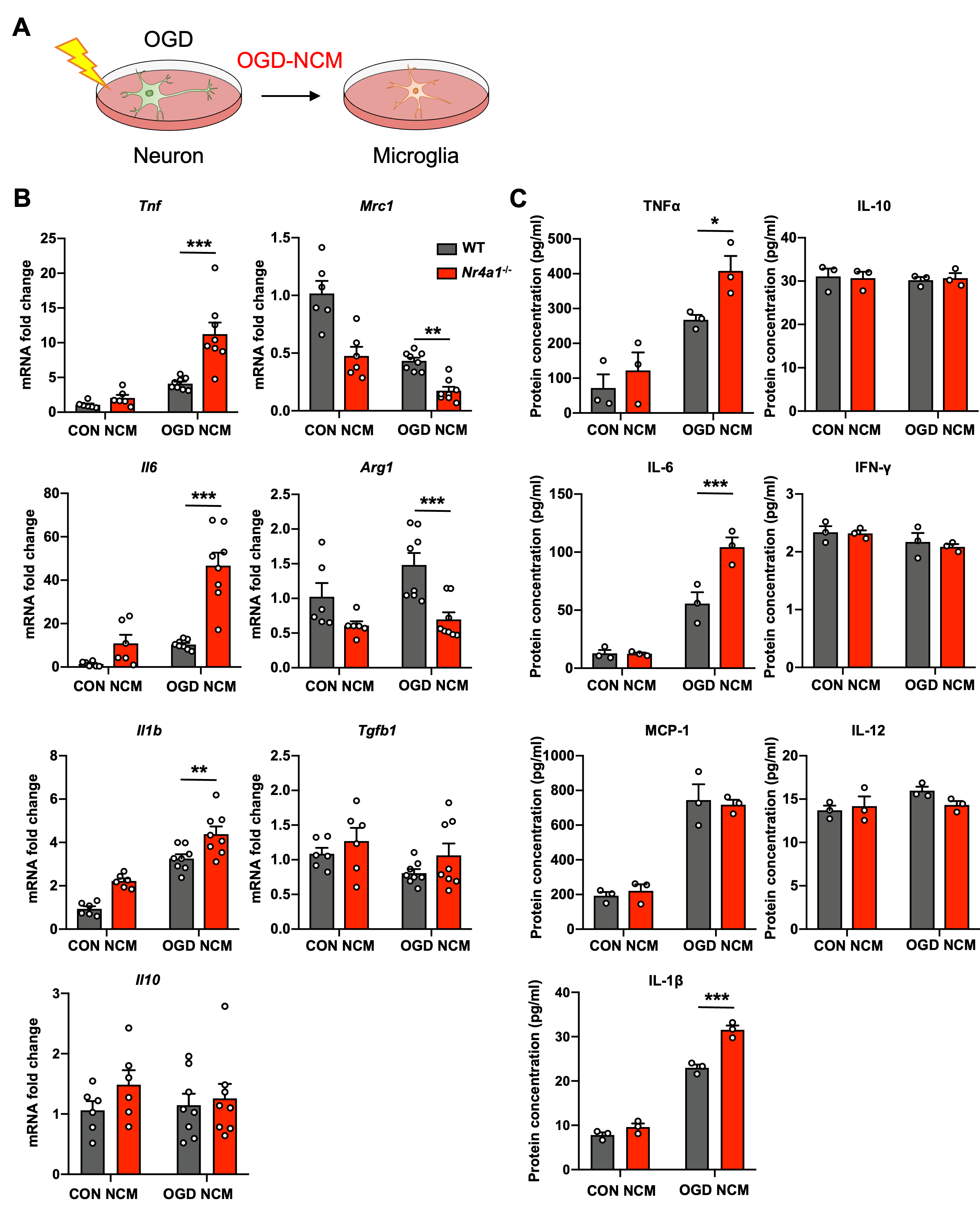

Supplement: S6 Fig — (A) Paradigm of the preparation of oxygen-glucose deprivation-treated neuron-conditioned media (OGD-NCM). (B, C) mRNA (B) and protein (C) levels of several inflammatory factors in WT and Nr4a1-/- primary microglia exposed to conditioned medium from control or OGD-treated neurons (in B, n = 6 biological repeats for CON-NCM-treated WT and Nr4a1-/- primary microglia, n = 8 biological repeats for OGD-NCM-treated WT and Nr4a1-/- primary microglia; in C, n = 3 biological repeats in each group). Data are presented as mean ± SEM. In (B), (C), two-way ANOVA with post hoc Bonferroni’s test. *P < 0.05; **P < 0.01; ***P < 0.001. The underlying data for this figure can be found in S1 Data. (TIF) [file pbio.3002199.s006.tif]

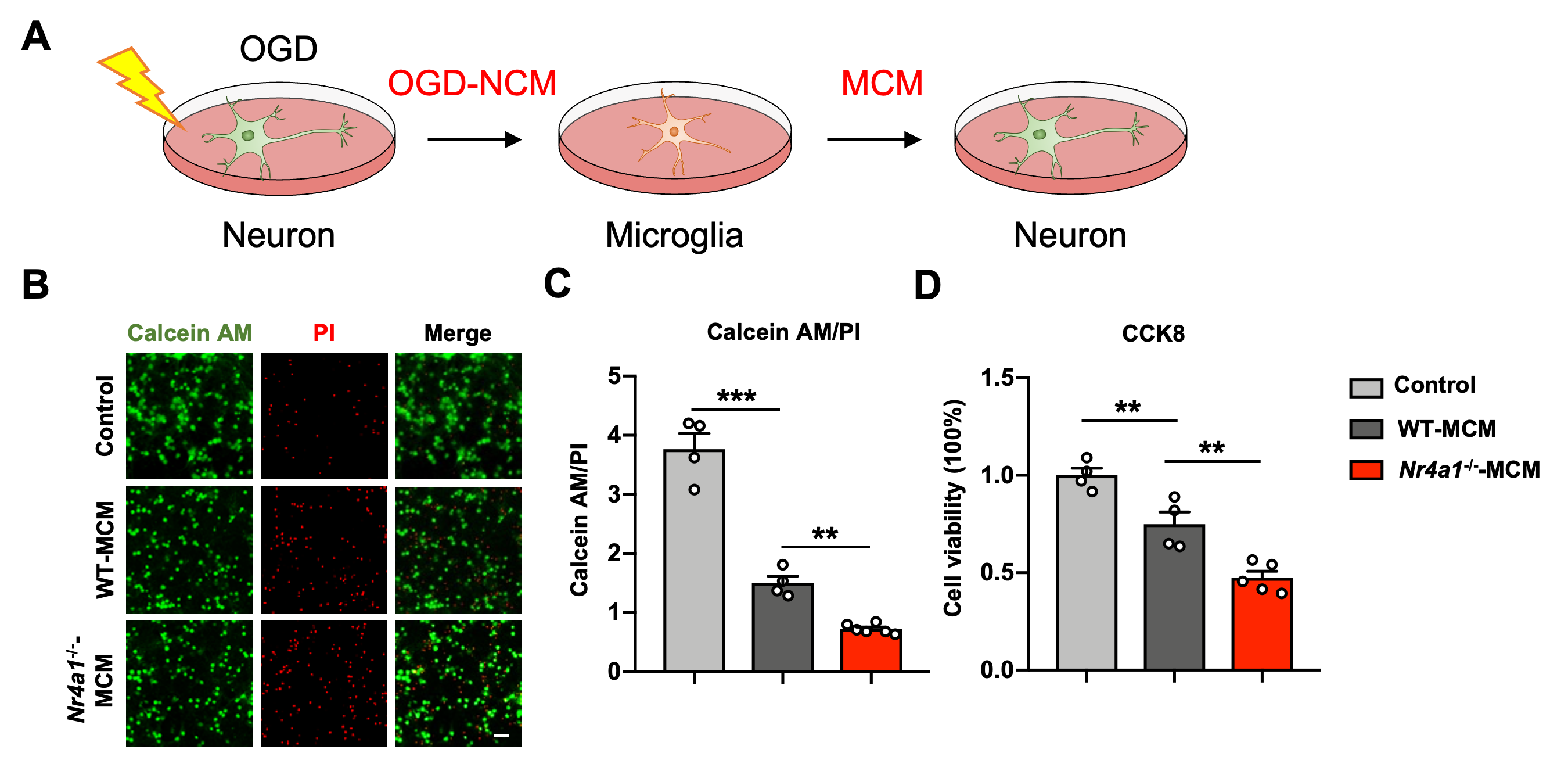

Supplement: S7 Fig — (A) Paradigm of the preparation of oxygen-glucose deprivation-treated neuron-conditioned media (OGD-NCM) and microglia-conditioned media (MCM). (B) Representative images of Calcein-AM/PI-stained primary neurons treated with conditioned medium from activated WT or Nr4a1-/- primary microglia. Scale bar, 20 μm. (C) Quantification of Calcein-AM/PI staining (n = 4, 4 and 6 biological repeats for control, WT MEM and Nr4a1-/- MEM). (D) Results of the CCK8 assay in primary neurons treated with conditioned medium from activated WT or Nr4a1-/- primary microglia (n = 4, 4 and 5 biological repeats for control, WT and Nr4a1-/- MEM). Data are presented as mean ± SEM. In (C), (D), one-way ANOVA with post hoc Dunnett’s test. **P < 0.01; ***P < 0.001. The underlying data for this figure can be found in S1 Data. (TIF) [file pbio.3002199.s007.tif]

**Fig 1A**

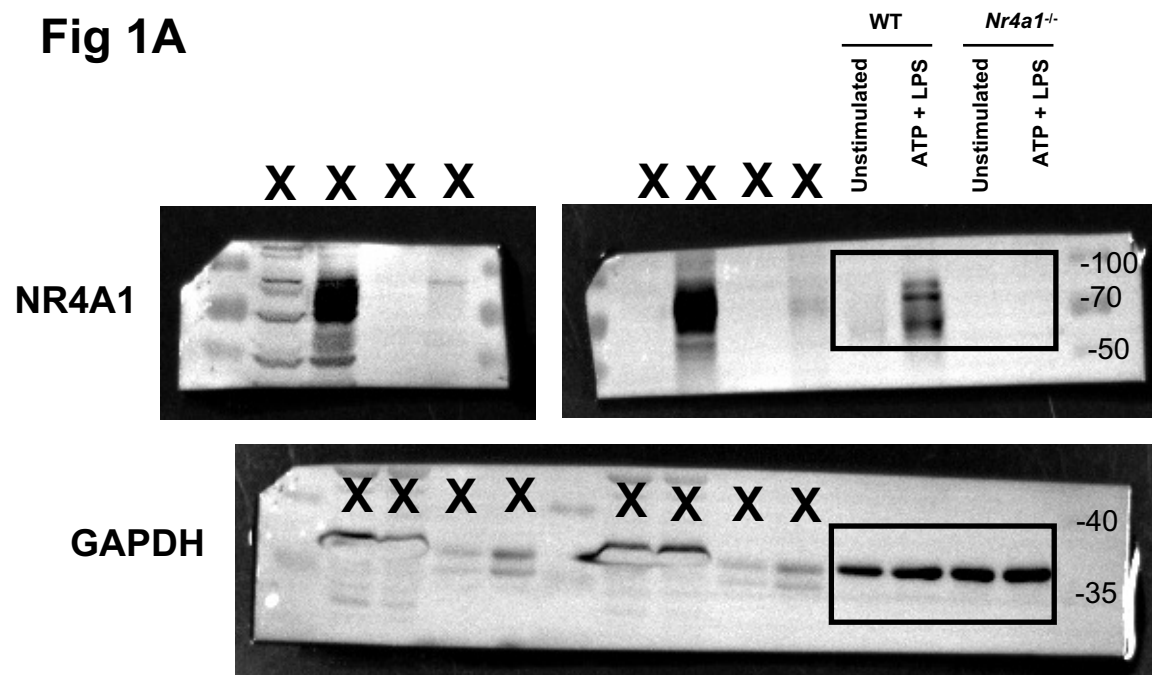

**Fig 1B**

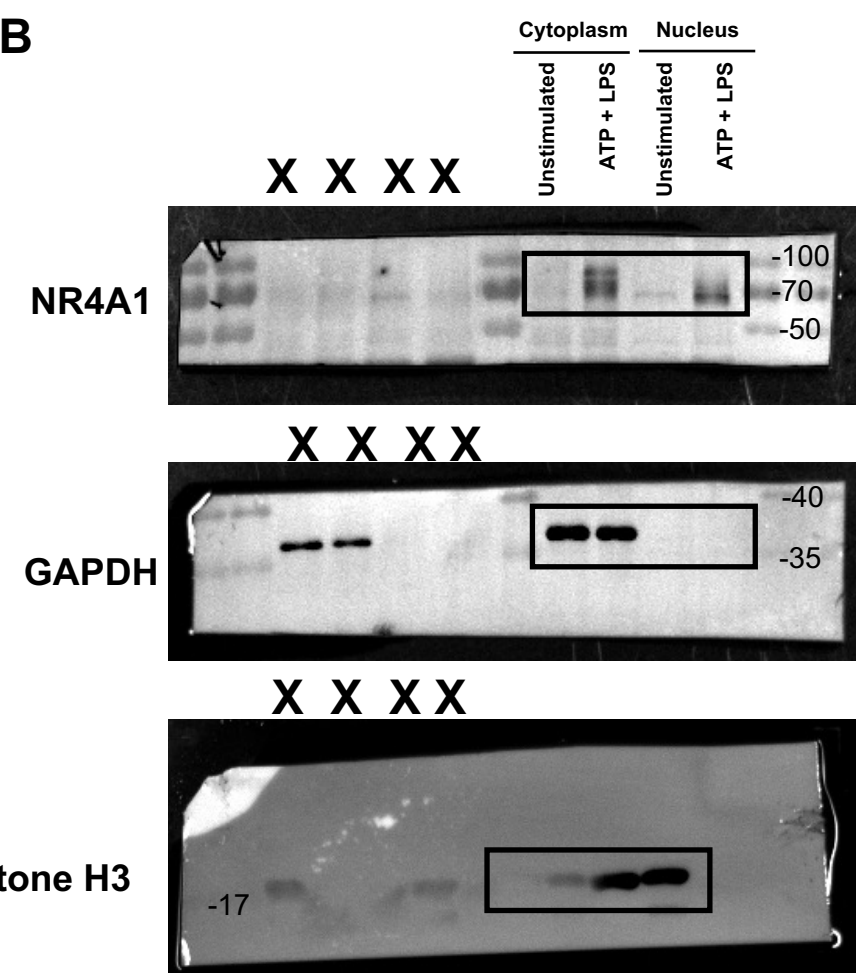

**Fig 1I**

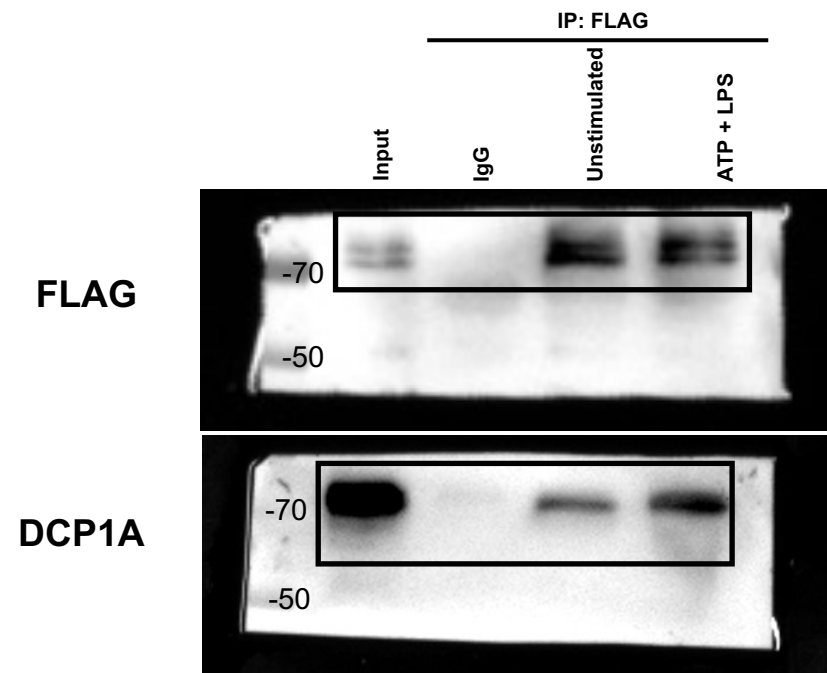

Fig 2D

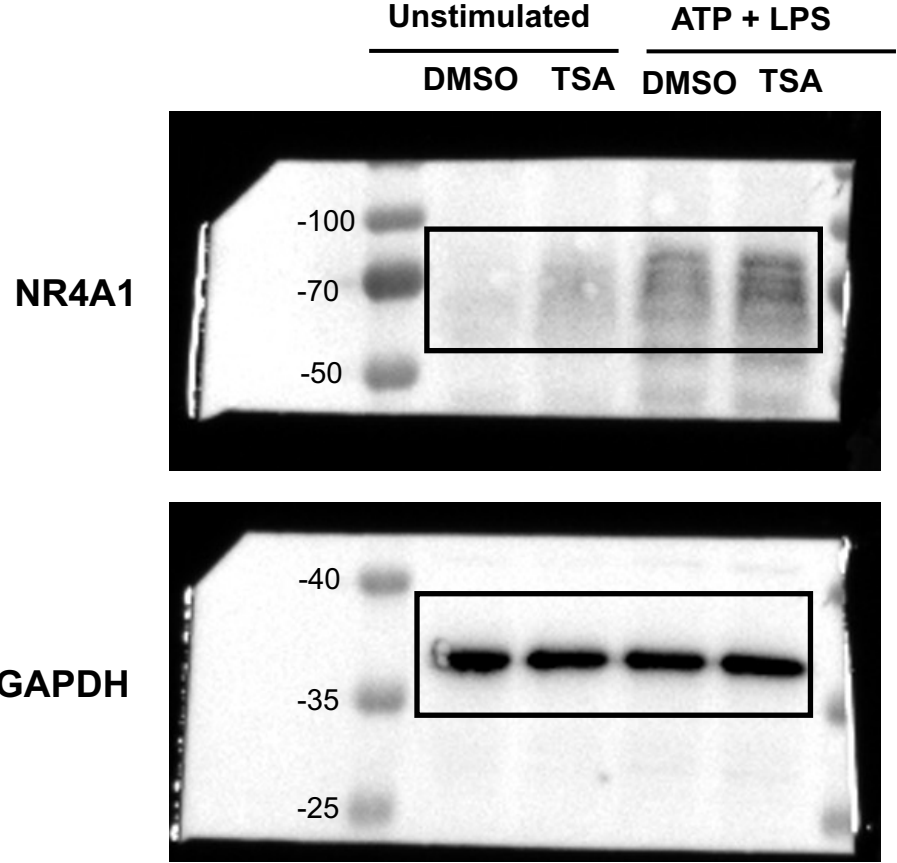

Fig 3D

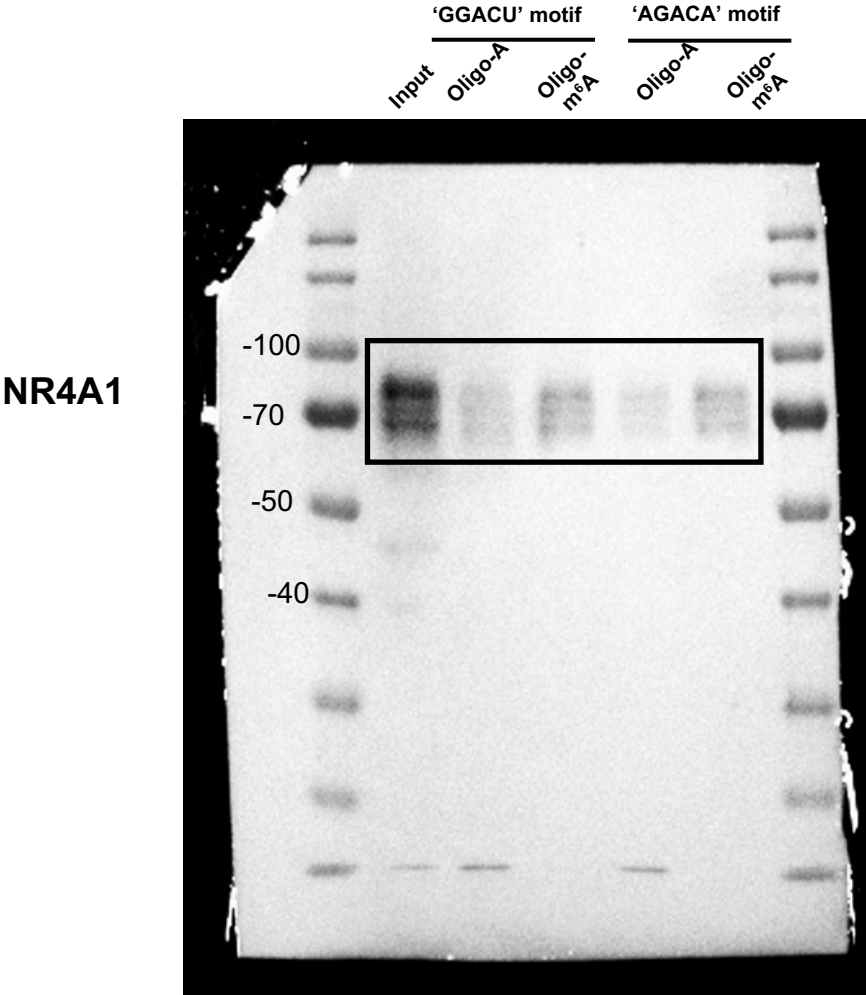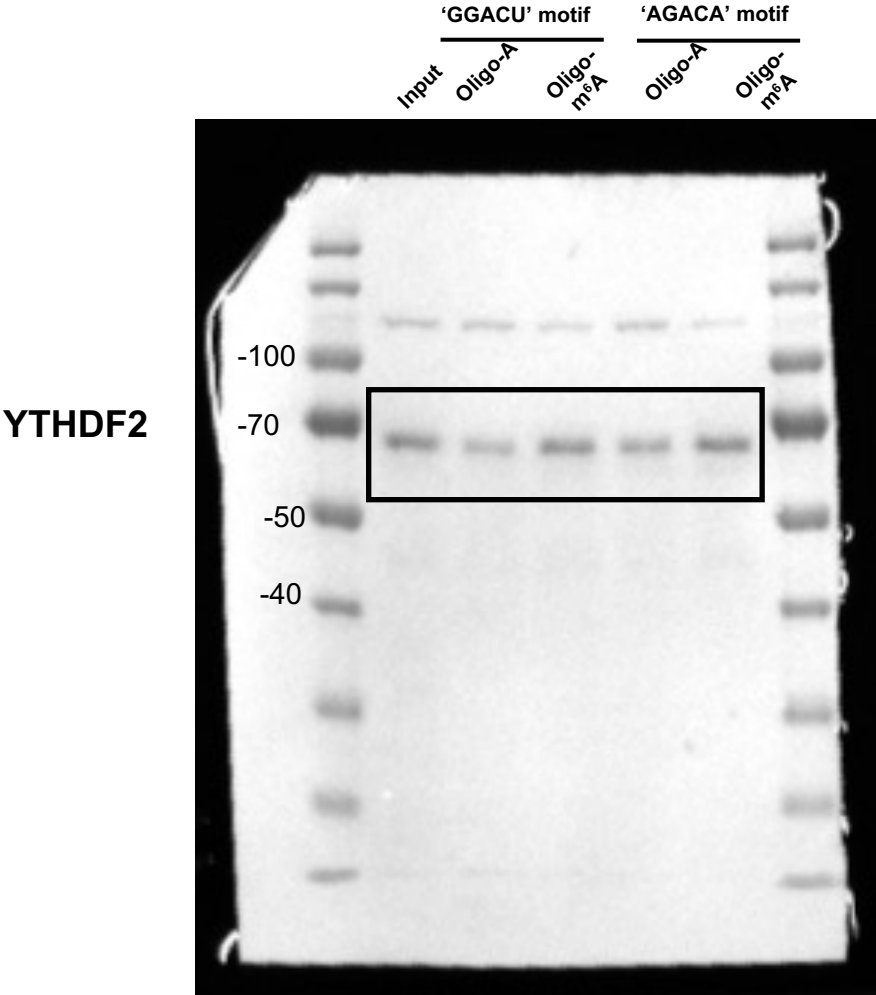

Fig 4A

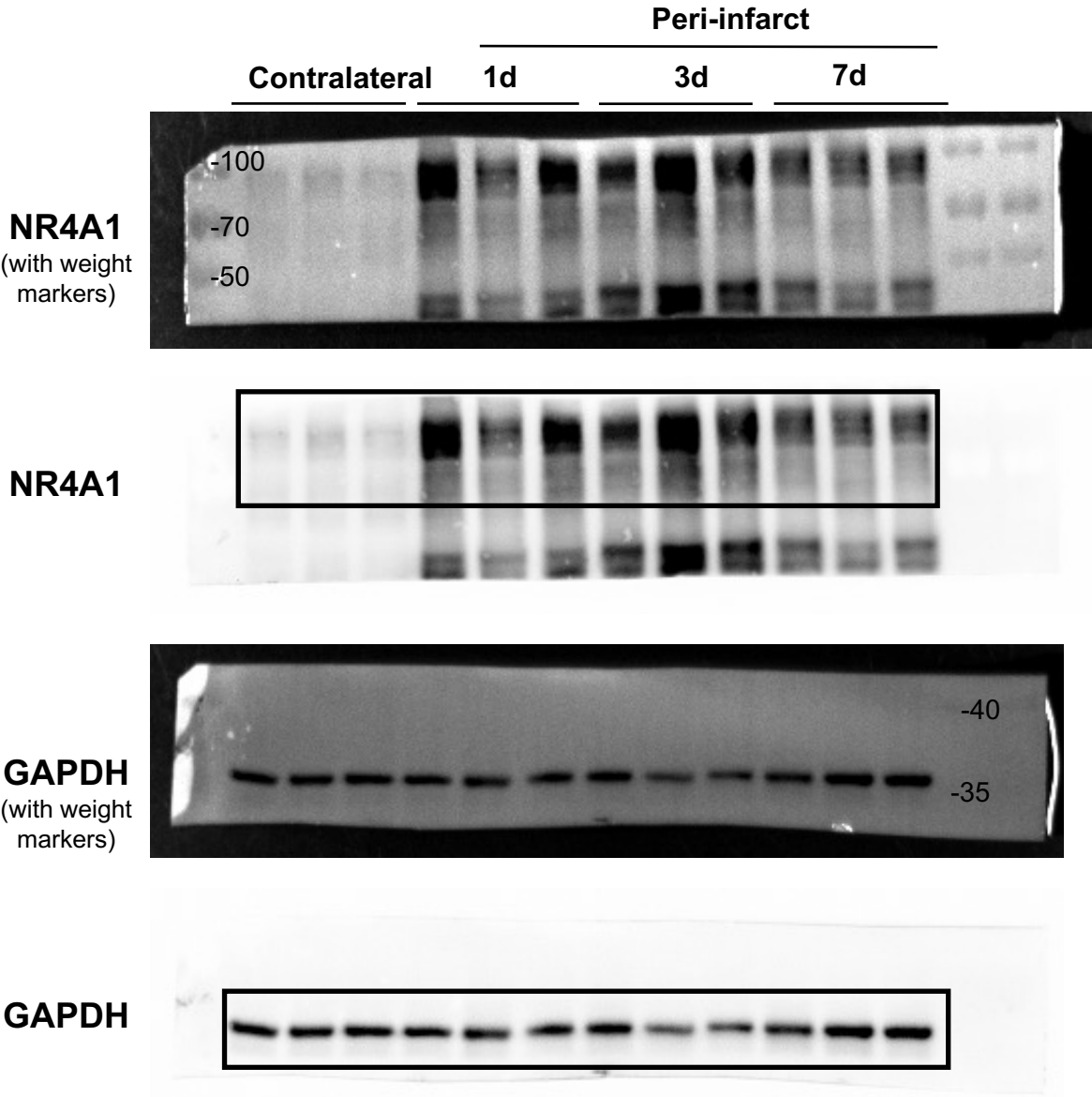

S1 Fig

NR4A1

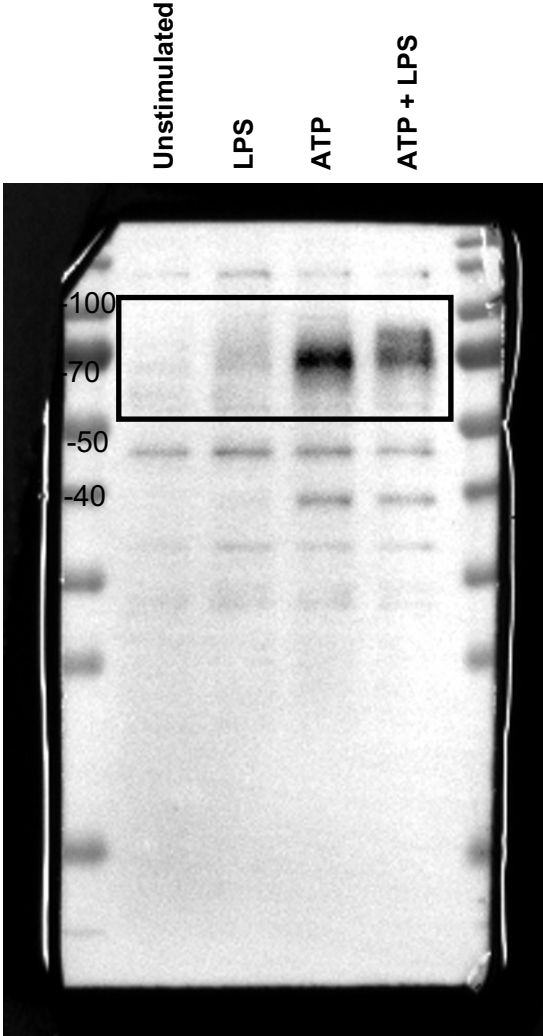

GAPDH

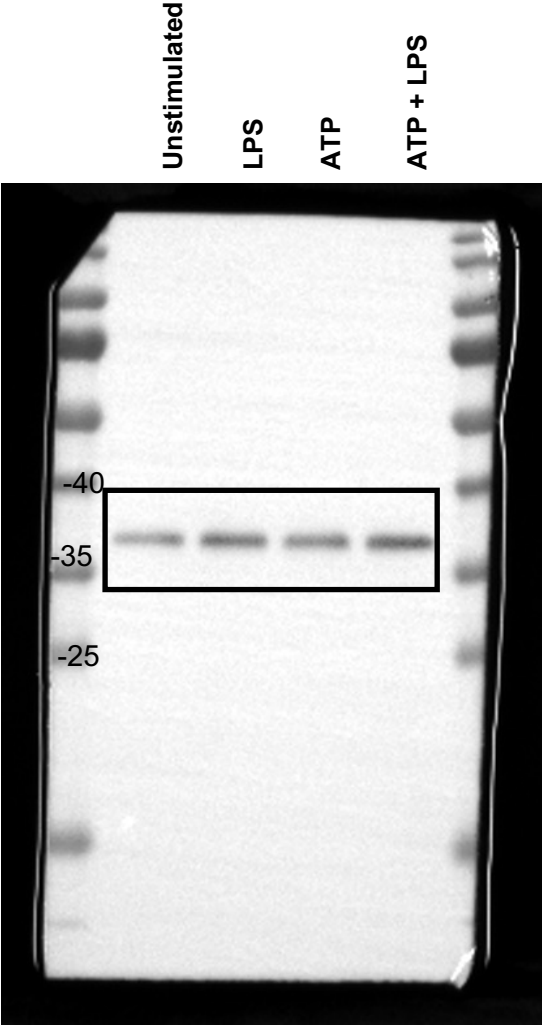

S2H Fig

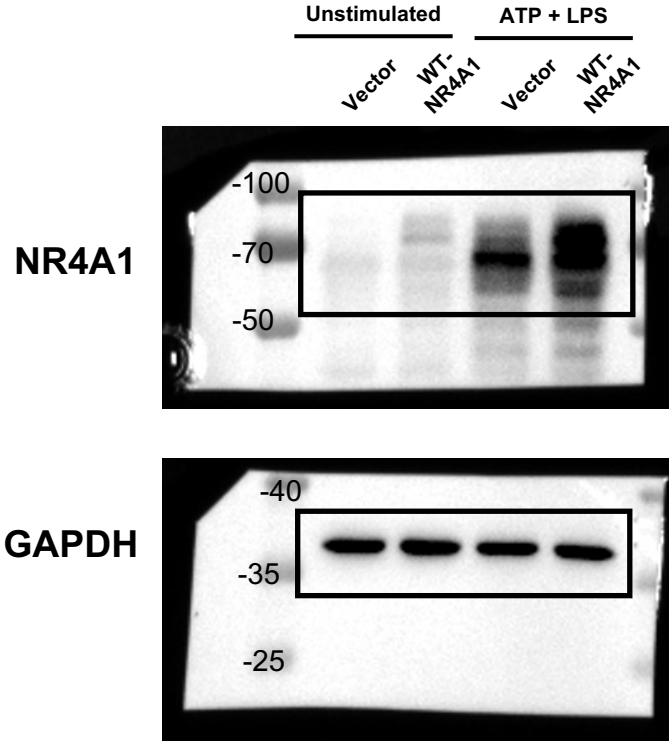

S2I Fig

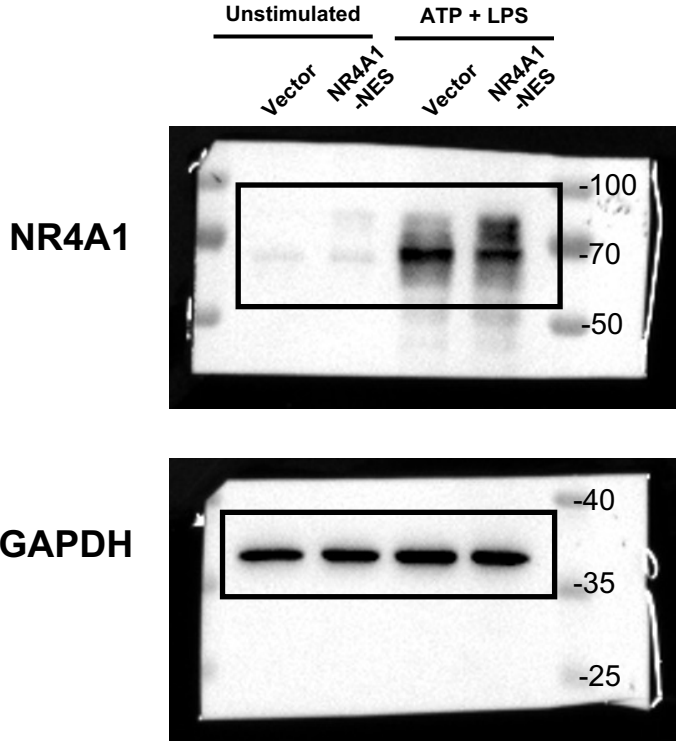

S3A Fig

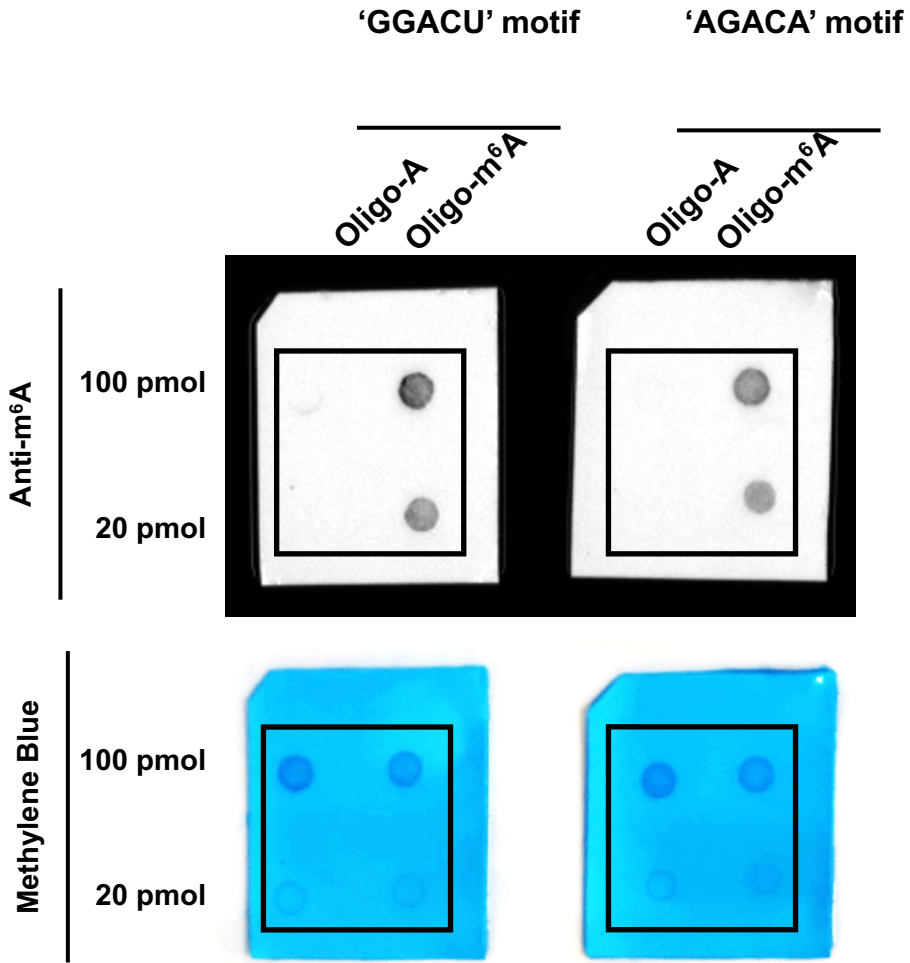

S3B Fig

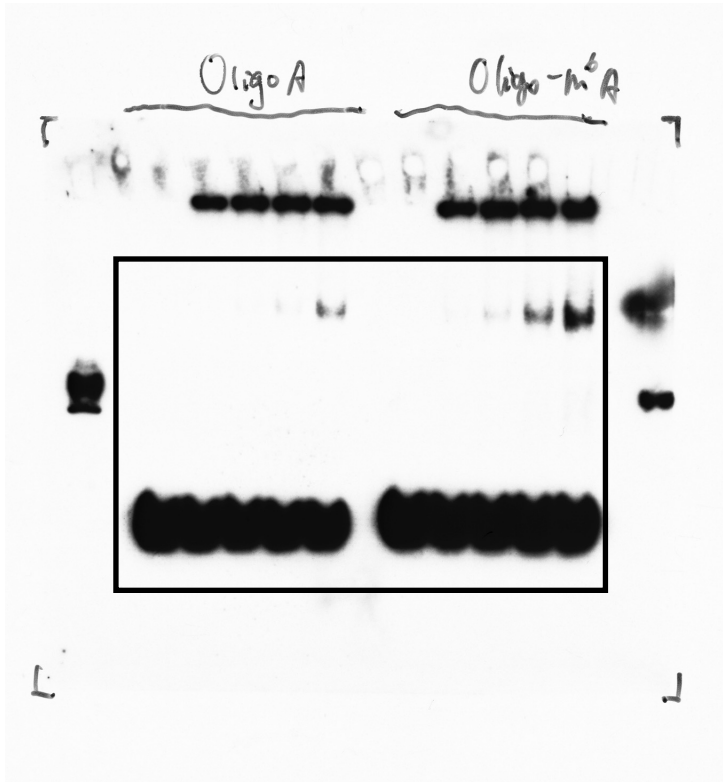

S3D Fig

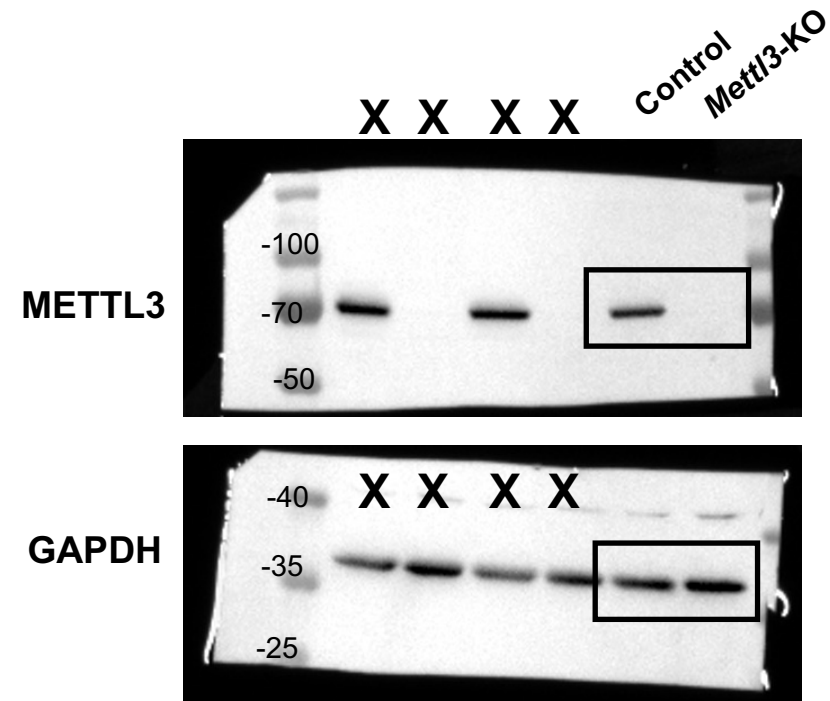

S4A Fig

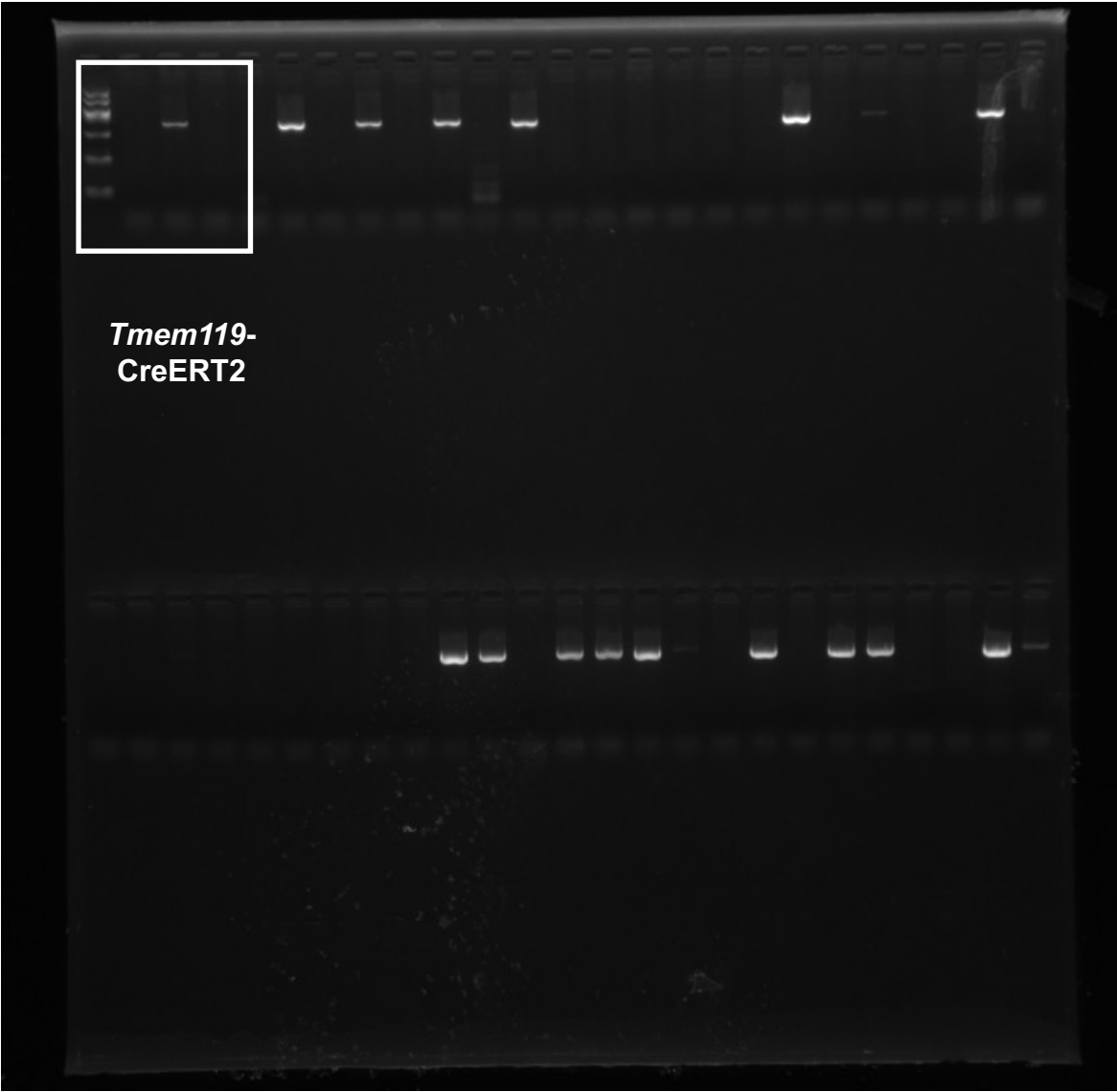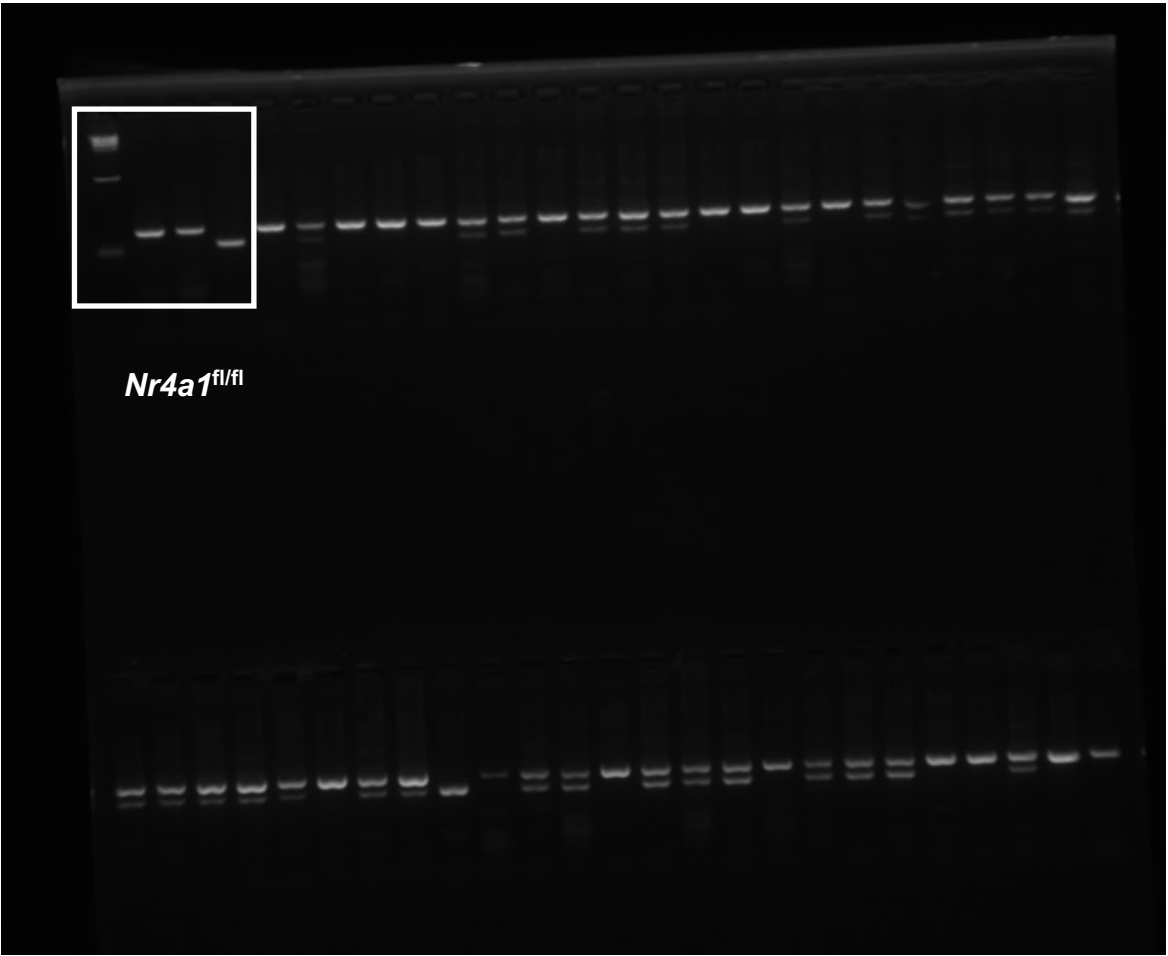

S4B Fig

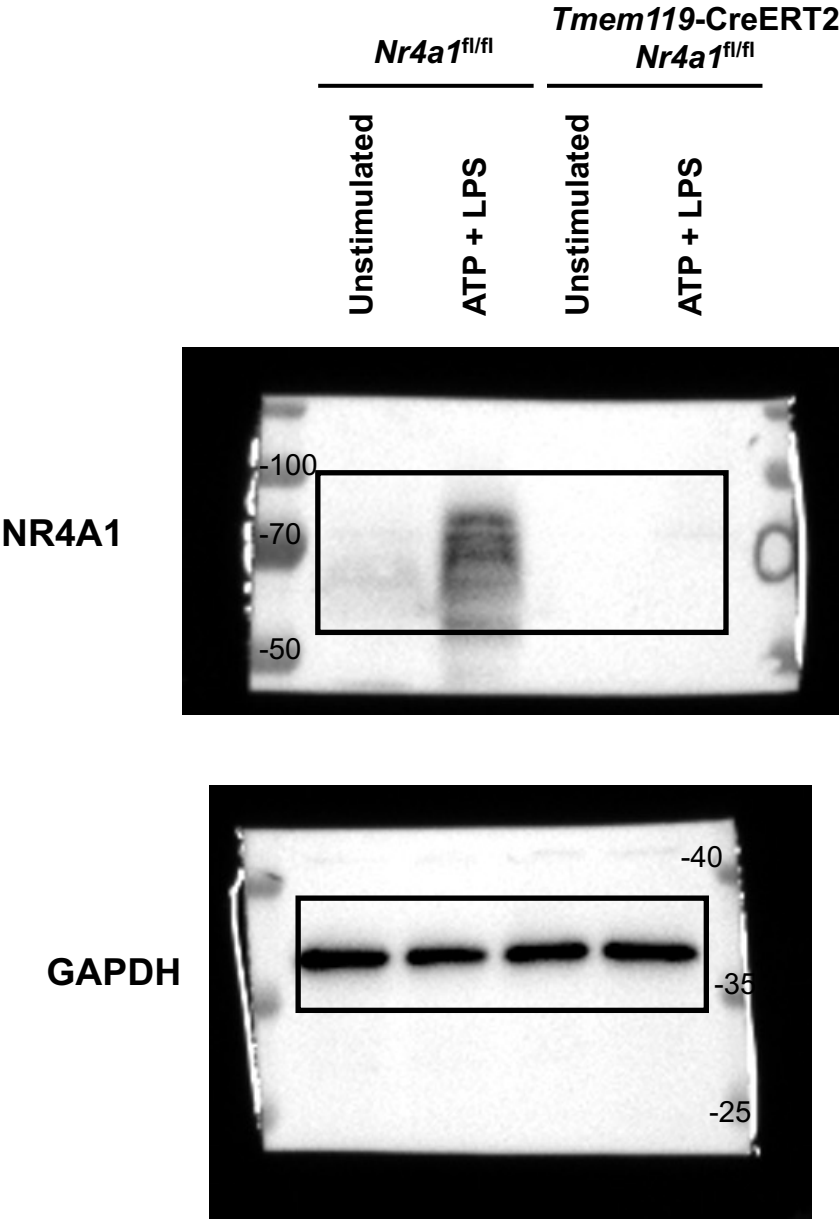

Supplement: S1 Raw Images — Original images supporting blot and gel results for Figs 1A and 1B, 1I; 2D; 3D; 4A; S1; S2H and S2I; S3A, S3B, and S3D; S4A and S4B. (PDF) [file pbio.3002199.s013.pdf]
