# Supplementary material for: Noncanonical contribution of microglial transcription factor NR4A1 to post-stroke recovery through TNF mRNA destabilization
Source: PLoS Biol. 2023 Jul 24;21(7):e3002199. doi: 10.1371/journal.pbio.3002199 (PMC10365314; doi:10.1371/journal.pbio.3002199)
Supplement: S1 Table — (DOCX) [file pbio.3002199.s008.docx]

| **Flag-NR4A1 IP** |  |  |  |  |  |  |  |
| --- | --- | --- | --- | --- | --- | --- | --- |
| **Accession** | **Gene names** | **MW [kDa]** | **Protein score** | **Sequence coverage (%)** | **# Unique Peptides** | **# Peptides** | **# PSMs** |
| Q8VDD5 | Myh9 | 226.23 | 2177.25 | 30.31 | 46 | 46 | 52 |
| P63017 | Hspa8 | 70.83 | 1830.60 | 43.96 | 22 | 25 | 44 |
| P52480 | Pkm | 57.81 | 1661.62 | 53.67 | 27 | 27 | 39 |
| P63038 | Hspd1 | 60.92 | 1396.19 | 43.28 | 21 | 21 | 29 |
| P20029 | Hspa5 | 72.38 | 1312.12 | 44.27 | 26 | 28 | 31 |
| P11499 | Hsp90ab1 | 83.23 | 1290.64 | 38.12 | 15 | 25 | 34 |
| P38647 | Hspa9 | 73.42 | 1195.56 | 41.24 | 23 | 24 | 26 |
| P60710 | Actb | 41.71 | 1184.95 | 45.33 | 6 | 13 | 27 |
| P09405 | Ncl | 76.68 | 1166.66 | 33.52 | 25 | 25 | 27 |
| P07901 | Hsp90aa1 | 84.73 | 1112.24 | 32.47 | 12 | 22 | 28 |
| Q03265 | Atp5f1a | 59.72 | 1065.35 | 48.28 | 24 | 24 | 28 |
| P99024 | Tubb5 | 49.64 | 1029.52 | 46.85 | 5 | 15 | 27 |
| P17182 | Eno1 | 47.11 | 1014.72 | 47.47 | 16 | 16 | 26 |
| P56480 | Atp5f1b | 56.27 | 997.41 | 47.07 | 16 | 16 | 22 |
| P09411 | Pgk1 | 44.52 | 890.23 | 39.57 | 15 | 15 | 21 |
| P68372 | Tubb4b | 49.80 | 845.27 | 42.92 | 3 | 14 | 21 |
| P26041 | Msn | 67.72 | 839.95 | 28.94 | 16 | 20 | 24 |
| P29341 | Pabpc1 | 70.63 | 802.88 | 30.19 | 17 | 17 | 20 |
| P40142 | Tkt | 67.59 | 797.55 | 32.91 | 14 | 14 | 20 |
| Q99MR8 | Mccc1 | 79.29 | 789.29 | 29.85 | 16 | 16 | 19 |
| P16858 | Gapdh | 35.79 | 780.85 | 38.44 | 9 | 9 | 20 |
| P05064 | Aldoa | 39.33 | 768.71 | 40.93 | 13 | 13 | 17 |
| P58252 | Eef2 | 95.25 | 767.35 | 20.75 | 17 | 17 | 24 |
| Q9Z1Q9 | Vars | 140.13 | 709.12 | 19.24 | 19 | 19 | 20 |
| P08113 | Hsp90b1 | 92.42 | 707.59 | 25.94 | 16 | 16 | 17 |
| Q3ULD5 | Mccc2 | 61.34 | 700.09 | 25.58 | 12 | 12 | 16 |
| P80318 | Cct3 | 60.59 | 698.40 | 33.21 | 15 | 15 | 17 |
| Q68FD5 | Cltc | 191.43 | 690.76 | 15.76 | 19 | 19 | 19 |
| Q02053 | Uba1 | 117.73 | 687.09 | 19.28 | 15 | 15 | 18 |
| P60843 | Eif4a1 | 46.12 | 678.01 | 39.16 | 15 | 15 | 18 |
| P68373 | Tuba1c | 49.88 | 660.81 | 34.74 | 11 | 11 | 16 |
| Q61316 | Hspa4 | 94.07 | 660.11 | 22.83 | 16 | 16 | 18 |
| P42932 | Cct8 | 59.52 | 655.29 | 35.22 | 17 | 17 | 17 |
| Q9EQK5 | Mvp | 95.87 | 617.60 | 25.55 | 17 | 17 | 17 |
| P23116 | Eif3a | 161.84 | 615.90 | 16.15 | 18 | 18 | 18 |
| Q9D8E6 | Rpl4 | 47.12 | 607.58 | 30.55 | 13 | 13 | 18 |
| Q61233 | Lcp1 | 70.10 | 602.44 | 29.03 | 16 | 16 | 16 |
| P27773 | Pdia3 | 56.64 | 601.91 | 37.82 | 16 | 16 | 18 |
| P80314 | Cct2 | 57.44 | 598.44 | 30.47 | 12 | 12 | 14 |
| P26039 | Tln1 | 269.65 | 593.11 | 7.63 | 15 | 15 | 15 |
| P10126 | Eef1a1 | 50.08 | 586.15 | 25.54 | 10 | 10 | 16 |
| Q8VDN2 | Atp1a1 | 112.91 | 574.70 | 16.62 | 14 | 14 | 14 |
| P06151 | Ldha | 36.48 | 563.27 | 36.14 | 11 | 11 | 17 |
| P61979 | Hnrnpk | 50.94 | 548.48 | 34.13 | 13 | 13 | 13 |
| Q9JKF1 | Iqgap1 | 188.62 | 540.95 | 11.83 | 17 | 17 | 17 |
| P09103 | P4hb | 57.02 | 540.93 | 24.56 | 11 | 11 | 13 |
| P11983 | Tcp1 | 60.41 | 523.45 | 24.1 | 12 | 12 | 14 |
| Q922F4 | Tubb6 | 50.06 | 521.64 | 23.27 | 4 | 9 | 12 |
| Q9CZN7 | Shmt2 | 55.72 | 517.68 | 26.79 | 12 | 12 | 13 |
| P68033 | Actc1 | 41.99 | 513.87 | 24.67 | 1 | 8 | 16 |
| P29351 | Ptpn6 | 67.52 | 512.15 | 23.19 | 12 | 12 | 12 |
| P10649 | Gstm1 | 25.95 | 503.15 | 45.87 | 9 | 9 | 10 |
| P50516 | Atp6v1a | 68.28 | 502.19 | 22.04 | 12 | 12 | 15 |
| Q99K85 | Psat1 | 40.45 | 497.77 | 35.68 | 11 | 11 | 12 |
| Q8CGC7 | Eprs | 169.97 | 494.96 | 9.79 | 13 | 13 | 14 |
| P06745 | Gpi | 62.73 | 464.65 | 24.91 | 12 | 12 | 14 |
| P47738 | Aldh2 | 56.50 | 449.91 | 28.13 | 11 | 11 | 12 |
| P60122 | Ruvbl1 | 50.18 | 439.26 | 28.51 | 9 | 9 | 10 |
| Q91YQ5 | Rpn1 | 68.49 | 434.19 | 23.52 | 10 | 10 | 10 |
| P80315 | Cct4 | 58.03 | 433.34 | 23.01 | 12 | 12 | 13 |
| Q7TPV4 | Mybbp1a | 151.94 | 432.70 | 8.63 | 10 | 10 | 10 |
| Q922B2 | Dars | 57.11 | 426.94 | 22.55 | 10 | 10 | 10 |
| Q9DBJ1 | Pgam1 | 28.81 | 416.98 | 41.34 | 9 | 9 | 12 |
| P08249 | Mdh2 | 35.59 | 412.37 | 36.39 | 9 | 9 | 11 |
| Q8BGQ7 | Aars | 106.84 | 403.53 | 11.98 | 9 | 9 | 9 |
| P47911 | Rpl6 | 33.49 | 394.56 | 30.41 | 10 | 10 | 13 |
| Q6ZWX6 | Eif2s1 | 36.09 | 390.67 | 22.22 | 7 | 7 | 7 |
| O55143 | Atp2a2 | 114.78 | 390.17 | 11.02 | 9 | 9 | 10 |
| Q9D8N0 | Eef1g | 50.03 | 389.55 | 24.94 | 9 | 9 | 11 |
| P80313 | Cct7 | 59.61 | 388.68 | 26.1 | 12 | 12 | 12 |
| P80316 | Cct5 | 59.59 | 385.50 | 18.85 | 10 | 10 | 10 |
| Q8BMF4 | Dlat | 67.90 | 382.97 | 17.45 | 8 | 8 | 8 |
| Q8R1B4 | Eif3c | 105.46 | 368.62 | 8.67 | 9 | 9 | 9 |
| P05202 | Got2 | 47.38 | 368.04 | 20.7 | 8 | 8 | 10 |
| Q8VEK3 | Hnrnpu | 87.86 | 364.19 | 11.5 | 8 | 8 | 9 |
| P05201 | Got1 | 46.22 | 359.68 | 21.07 | 7 | 7 | 8 |
| P62908 | Rps3 | 26.66 | 358.73 | 42.8 | 10 | 10 | 10 |
| O08749 | Dld | 54.24 | 354.65 | 17.68 | 8 | 8 | 11 |
| P48036 | Anxa5 | 35.73 | 354.46 | 25.39 | 7 | 7 | 8 |
| Q922U2 | Krt5 | 61.73 | 352.23 | 11.38 | 3 | 8 | 10 |
| Q9Z0N1 | Eif2s3x | 51.03 | 347.65 | 16.53 | 8 | 8 | 10 |
| Q9ERK4 | Cse1l | 110.38 | 346.64 | 11.95 | 9 | 9 | 9 |
| Q8JZQ9 | Eif3b | 91.31 | 345.73 | 15.57 | 11 | 11 | 12 |
| Q8QZY1 | Eif3l | 66.57 | 341.45 | 14.01 | 8 | 8 | 8 |
| P80317 | Cct6a | 57.97 | 336.90 | 22.98 | 11 | 11 | 11 |
| P07356 | Anxa2 | 38.65 | 336.28 | 31.27 | 9 | 9 | 9 |
| O35129 | Phb2 | 33.28 | 334.78 | 31.44 | 8 | 8 | 8 |
| Q9D0E1 | Hnrnpm | 77.60 | 333.84 | 17.28 | 10 | 10 | 10 |
| P14211 | Calr | 47.96 | 332.69 | 20.91 | 6 | 6 | 9 |
| Q9WUA3 | Pfkp | 85.40 | 332.25 | 12.63 | 7 | 8 | 8 |
| Q61753 | Phgdh | 56.55 | 330.87 | 16.14 | 9 | 9 | 9 |
| P63101 | Ywhaz | 27.75 | 330.28 | 29.39 | 3 | 7 | 8 |
| Q78PY7 | Snd1 | 102.02 | 322.89 | 10.55 | 7 | 7 | 8 |
| P24452 | Capg | 39.22 | 320.32 | 22.73 | 5 | 5 | 6 |
| E9Q634 | Myo1e | 126.74 | 316.78 | 8.94 | 8 | 8 | 8 |
| Q8VIJ6 | Sfpq | 75.39 | 315.81 | 14.88 | 8 | 9 | 10 |
| P12970 | Rpl7a | 29.96 | 315.43 | 27.44 | 9 | 9 | 9 |
| Q6ZWN5 | Rps9 | 22.58 | 311.84 | 43.3 | 9 | 9 | 9 |
| Q9CWJ9 | Atic | 64.18 | 311.51 | 19.59 | 9 | 9 | 9 |
| P50247 | Ahcy | 47.66 | 309.24 | 17.36 | 7 | 7 | 7 |
| P62702 | Rps4x | 29.58 | 308.44 | 35.74 | 10 | 10 | 10 |
| P50580 | Pa2g4 | 43.67 | 307.71 | 20.3 | 7 | 7 | 9 |
| Q3TW96 | Uap1l1 | 56.58 | 306.05 | 20.12 | 10 | 10 | 10 |
| Q8BFR5 | Tufm | 49.48 | 305.82 | 18.14 | 6 | 7 | 7 |
| Q9CZD3 | Gars | 81.83 | 305.54 | 11.11 | 8 | 8 | 8 |
| P60229 | Eif3e | 52.19 | 305.37 | 24.94 | 10 | 10 | 11 |
| Q99KI0 | Aco2 | 85.41 | 304.52 | 15 | 10 | 10 | 10 |
| Q9D0I9 | Rars | 75.63 | 304.30 | 15.3 | 9 | 9 | 9 |
| Q9CQV8 | Ywhab | 28.07 | 302.06 | 23.98 | 2 | 6 | 8 |
| P47962 | Rpl5 | 34.38 | 296.14 | 22.9 | 6 | 6 | 8 |
| Q7TMK9 | Syncrip | 69.59 | 295.11 | 13 | 6 | 6 | 6 |
| Q8BU30 | Iars | 144.18 | 292.06 | 7.45 | 8 | 8 | 8 |
| Q9CZ30 | Ola1 | 44.70 | 291.41 | 18.94 | 7 | 7 | 8 |
| P51881 | Slc25a5 | 32.91 | 286.84 | 26.85 | 3 | 8 | 9 |
| Q93092 | Taldo1 | 37.36 | 286.51 | 18.1 | 8 | 8 | 9 |
| Q91WQ3 | Yars | 59.07 | 286.32 | 13.83 | 7 | 7 | 7 |
| P62754 | Rps6 | 28.66 | 284.11 | 24.1 | 6 | 6 | 8 |
| Q8BHN3 | Ganab | 106.84 | 283.57 | 8.16 | 6 | 6 | 7 |
| P62814 | Atp6v1b2 | 56.51 | 282.41 | 19.18 | 7 | 7 | 7 |
| Q9DBG6 | Rpn2 | 69.02 | 281.56 | 15.37 | 7 | 7 | 7 |
| P28658 | Atxn10 | 53.67 | 281.13 | 16.21 | 6 | 6 | 6 |
| Q8R180 | Ero1a | 54.05 | 278.88 | 12.93 | 5 | 5 | 5 |
| Q01853 | Vcp | 89.27 | 278.53 | 9.43 | 6 | 6 | 7 |
| Q922D8 | Mthfd1 | 101.14 | 278.24 | 8.66 | 7 | 7 | 7 |
| P26040 | Ezr | 69.36 | 277.60 | 8.02 | 2 | 6 | 7 |
| Q9D0K2 | Oxct1 | 55.95 | 277.34 | 14.04 | 6 | 6 | 6 |
| Q61598 | Gdi2 | 50.50 | 274.85 | 24.49 | 8 | 8 | 8 |
| Q76MZ3 | Ppp2r1a | 65.28 | 274.34 | 16.98 | 7 | 7 | 8 |
| Q6NZJ6 | Eif4g1 | 175.97 | 270.71 | 6.06 | 9 | 9 | 9 |
| Q922R8 | Pdia6 | 48.07 | 266.69 | 21.59 | 7 | 7 | 7 |
| P12382 | Pfkl | 85.31 | 264.83 | 10.51 | 4 | 5 | 5 |
| Q62318 | Trim28 | 88.79 | 264.61 | 11.87 | 8 | 8 | 8 |
| P14869 | Rplp0 | 34.19 | 263.75 | 25.55 | 7 | 7 | 8 |
| O35737 | Hnrnph1 | 49.17 | 261.59 | 18.93 | 4 | 6 | 8 |
| P62960 | Ybx1 | 35.71 | 261.37 | 24.22 | 3 | 4 | 4 |
| Q62167 | Ddx3x | 73.06 | 261.34 | 13.6 | 6 | 7 | 7 |
| P54071 | Idh2 | 50.87 | 260.18 | 14.6 | 6 | 6 | 7 |
| P48962 | Slc25a4 | 32.88 | 259.69 | 22.82 | 2 | 7 | 8 |
| Q9DCH4 | Eif3f | 37.96 | 259.34 | 19.11 | 5 | 5 | 5 |
| Q99L47 | St13 | 41.63 | 258.39 | 18.33 | 6 | 6 | 6 |
| Q501J6 | Ddx17 | 72.35 | 257.05 | 11.08 | 3 | 6 | 6 |
| P26638 | Sars | 58.35 | 255.86 | 12.11 | 5 | 5 | 6 |
| Q8VED5 | Krt79 | 57.52 | 254.44 | 7.34 | 1 | 5 | 7 |
| Q8BMJ2 | Lars | 134.11 | 254.33 | 6.2 | 6 | 6 | 6 |
| Q62465 | Vat1 | 43.07 | 254.20 | 18.47 | 6 | 6 | 6 |
| P46471 | Psmc2 | 48.62 | 253.73 | 12.7 | 5 | 5 | 5 |
| Q64337 | Sqstm1 | 48.13 | 252.98 | 13.12 | 4 | 4 | 5 |
| Q3TTY5 | Krt2 | 70.88 | 252.09 | 6.65 | 3 | 5 | 6 |
| Q61656 | Ddx5 | 69.25 | 251.53 | 12.7 | 4 | 7 | 7 |
| P16460 | Ass1 | 46.56 | 245.86 | 14.08 | 8 | 8 | 8 |
| Q80X90 | Flnb | 277.65 | 244.94 | 2.73 | 4 | 6 | 6 |
| Q99MN1 | Kars | 67.80 | 242.47 | 11.43 | 6 | 6 | 7 |
| P50446 | Krt6a | 59.30 | 242.45 | 9.76 | 1 | 6 | 7 |
| Q00612 | G6pdx | 59.22 | 240.75 | 14.95 | 7 | 7 | 7 |
| P14206 | Rpsa | 32.82 | 240.33 | 12.88 | 3 | 3 | 4 |
| Q8BTM8 | Flna | 281.05 | 239.17 | 3.82 | 6 | 8 | 8 |
| P47963 | Rpl13 | 24.29 | 238.40 | 21.8 | 5 | 5 | 6 |
| Q9DCD0 | Pgd | 53.21 | 237.15 | 14.91 | 6 | 6 | 6 |
| Q61937 | Npm1 | 32.54 | 237.10 | 19.18 | 5 | 5 | 5 |
| Q9CQN1 | Trap1 | 80.16 | 236.76 | 7.08 | 3 | 4 | 4 |
| P61982 | Ywhag | 28.28 | 235.65 | 19.43 | 2 | 5 | 6 |
| P57776 | Eef1d | 31.27 | 234.90 | 25.98 | 6 | 6 | 6 |
| Q60854 | Serpinb6 | 42.57 | 234.87 | 12.96 | 5 | 5 | 5 |
| P62196 | Psmc5 | 45.60 | 234.54 | 14.29 | 3 | 4 | 4 |
| P28352 | Apex1 | 35.47 | 233.70 | 13.56 | 3 | 3 | 4 |
| P68254 | Ywhaq | 27.76 | 231.14 | 19.59 | 1 | 5 | 6 |
| P26443 | Glud1 | 61.30 | 231.09 | 12.54 | 6 | 6 | 6 |
| P62259 | Ywhae | 29.16 | 230.27 | 20.78 | 3 | 6 | 7 |
| Q9D2G2 | Dlst | 48.96 | 230.18 | 12.56 | 6 | 6 | 6 |
| O35639 | Anxa3 | 36.36 | 229.84 | 18.58 | 5 | 5 | 5 |
| Q8BGZ7 | Krt75 | 59.70 | 229.45 | 8.53 | 1 | 5 | 6 |
| P26043 | Rdx | 68.50 | 228.76 | 7.03 | 1 | 5 | 6 |
| O35685 | Nudc | 38.33 | 228.39 | 16.87 | 5 | 5 | 5 |
| Q9EPL8 | Ipo7 | 119.41 | 227.92 | 5.11 | 4 | 4 | 4 |
| O88569 | Hnrnpa2b1 | 37.38 | 226.82 | 13.03 | 4 | 5 | 7 |
| Q6PB66 | Lrpprc | 156.52 | 226.80 | 5.03 | 6 | 6 | 6 |
| P32067 | Ssb | 47.73 | 226.71 | 14.94 | 6 | 6 | 6 |
| Q60973 | Rbbp7 | 47.76 | 225.97 | 15.53 | 2 | 5 | 5 |
| P27659 | Rpl3 | 46.08 | 225.36 | 14.64 | 7 | 7 | 7 |
| Q9D1A2 | Cndp2 | 52.73 | 225.13 | 16.42 | 5 | 5 | 5 |
| P16110 | Lgals3 | 27.50 | 225.06 | 18.56 | 6 | 6 | 7 |
| P61205 | Arf3 | 20.59 | 224.77 | 28.18 | 5 | 5 | 5 |
| Q60817 | Naca | 23.37 | 224.23 | 25.58 | 4 | 4 | 4 |
| P04104 | Krt1 | 65.57 | 222.51 | 5.97 | 2 | 4 | 5 |
| P35980 | Rpl18 | 21.63 | 221.90 | 37.23 | 6 | 6 | 6 |
| P97351 | Rps3a | 29.87 | 221.68 | 16.67 | 5 | 5 | 7 |
| Q8VDW0 | Ddx39a | 49.04 | 220.00 | 11.71 | 5 | 5 | 6 |
| P62242 | Rps8 | 24.19 | 217.99 | 25 | 4 | 4 | 5 |
| P62821 | Rab1A | 22.66 | 215.47 | 28.78 | 4 | 5 | 5 |
| P25444 | Rps2 | 31.21 | 213.60 | 21.16 | 6 | 6 | 6 |
| Q9JKR6 | Hyou1 | 111.11 | 212.90 | 5.41 | 5 | 5 | 6 |
| Q9Z2X1 | Hnrnpf | 45.70 | 211.63 | 17.35 | 3 | 5 | 7 |
| Q9CZM2 | Rpl15 | 24.13 | 211.57 | 27.45 | 6 | 6 | 6 |
| Q8BK67 | Rcc2 | 55.95 | 211.46 | 11.35 | 5 | 5 | 6 |
| Q60864 | Stip1 | 62.54 | 209.60 | 16.02 | 7 | 7 | 7 |
| P97807 | Fh | 54.32 | 207.99 | 14 | 5 | 5 | 5 |
| Q6ZWV3 | Rpl10 | 24.59 | 207.22 | 19.63 | 4 | 4 | 5 |
| P02535 | Krt10 | 57.74 | 206.18 | 8.6 | 2 | 5 | 5 |
| P37040 | Por | 77.00 | 203.21 | 11.5 | 7 | 7 | 7 |
| P12813 | Nr4a1 | 64.70 | 201.24 | 12.48 | 5 | 5 | 6 |
| Q6ZQ38 | Cand1 | 136.24 | 199.73 | 5.45 | 5 | 5 | 5 |
| Q8BWY3 | Etf1 | 49.00 | 197.76 | 11.67 | 4 | 4 | 4 |
| P62827 | Ran | 24.41 | 197.48 | 29.63 | 5 | 5 | 5 |
| Q8BP47 | Nars | 64.24 | 195.24 | 9.48 | 4 | 4 | 4 |
| P53026 | Rpl10a | 24.90 | 192.07 | 16.13 | 3 | 3 | 5 |
| Q99JB2 | Stoml2 | 38.36 | 191.69 | 18.98 | 5 | 5 | 5 |
| P45376 | Akr1b1 | 35.71 | 191.36 | 17.09 | 5 | 5 | 5 |
| Q8CGK3 | Lonp1 | 105.78 | 188.93 | 8.64 | 6 | 6 | 6 |
| Q9CR57 | Rpl14 | 23.55 | 188.60 | 19.82 | 4 | 4 | 4 |
| Q61699 | Hsph1 | 96.35 | 188.02 | 6.53 | 5 | 5 | 5 |
| P24547 | Impdh2 | 55.78 | 187.85 | 12.45 | 5 | 5 | 5 |
| Q99PT1 | Arhgdia | 23.39 | 186.68 | 30.39 | 5 | 6 | 6 |
| O70251 | Eef1b | 24.68 | 186.42 | 14.67 | 2 | 2 | 2 |
| Q99K48 | Nono | 54.51 | 184.00 | 10.57 | 4 | 5 | 5 |
| Q8BMS1 | Hadha | 82.62 | 183.39 | 7.86 | 4 | 4 | 4 |
| Q60972 | Rbbp4 | 47.63 | 183.30 | 9.65 | 1 | 4 | 4 |
| Q91VI7 | Rnh1 | 49.78 | 182.61 | 11.4 | 4 | 4 | 4 |
| P17751 | Tpi1 | 32.17 | 182.13 | 18.06 | 4 | 4 | 5 |
| P54987 | Acod1 | 53.72 | 181.87 | 9.22 | 4 | 4 | 4 |
| Q3U1J4 | Ddb1 | 126.77 | 181.75 | 5.44 | 6 | 6 | 6 |
| Q60597 | Ogdh | 116.37 | 181.56 | 5.38 | 5 | 5 | 5 |
| Q9EQH3 | Vps35 | 91.65 | 180.76 | 7.29 | 5 | 5 | 5 |
| P17563 | Selenbp1 | 52.48 | 180.66 | 12.71 | 5 | 5 | 5 |
| P84099 | Rpl19 | 23.45 | 180.29 | 16.84 | 3 | 3 | 3 |
| Q9JMA1 | Usp14 | 55.97 | 178.19 | 9.53 | 4 | 4 | 4 |
| O88685 | Psmc3 | 49.52 | 177.18 | 19.23 | 7 | 7 | 7 |
| P17225 | Ptbp1 | 56.44 | 175.07 | 13.28 | 4 | 4 | 4 |
| P53395 | Dbt | 53.21 | 173.79 | 9.54 | 3 | 3 | 4 |
| Q9Z110 | Aldh18a1 | 87.21 | 173.35 | 5.41 | 4 | 4 | 4 |
| Q3U0V1 | Khsrp | 76.73 | 171.60 | 5.88 | 3 | 4 | 4 |
| P97311 | Mcm6 | 92.81 | 170.29 | 5.24 | 4 | 4 | 4 |
| P08730 | Krt13 | 47.72 | 170.17 | 9.15 | 1 | 5 | 5 |
| P17710 | Hk1 | 108.23 | 170.04 | 4.21 | 3 | 4 | 4 |
| P40124 | Cap1 | 51.53 | 169.87 | 11.81 | 4 | 4 | 4 |
| Q91VR2 | Atp5f1c | 32.87 | 169.63 | 13.76 | 4 | 4 | 4 |
| P68040 | Rack1 | 35.05 | 169.18 | 15.77 | 5 | 5 | 5 |
| Q8K2B3 | Sdha | 72.54 | 168.94 | 4.07 | 2 | 2 | 2 |
| P35564 | Canx | 67.24 | 168.24 | 9.48 | 5 | 5 | 5 |
| P13020 | Gsn | 85.89 | 168.18 | 5.9 | 4 | 4 | 4 |
| P19001 | Krt19 | 44.51 | 168.17 | 10.17 | 1 | 5 | 5 |
| P67778 | Phb | 29.80 | 167.57 | 15.44 | 5 | 5 | 5 |
| Q9CZU6 | Cs | 51.70 | 167.23 | 12.07 | 5 | 5 | 6 |
| Q91V92 | Acly | 119.65 | 165.67 | 4.4 | 4 | 4 | 4 |
| Q91WK2 | Eif3h | 39.81 | 165.28 | 11.08 | 3 | 3 | 3 |
| P82343 | Renbp | 49.74 | 165.14 | 7.67 | 2 | 2 | 2 |
| Q9JHU9 | Isyna1 | 60.89 | 164.65 | 11.31 | 4 | 4 | 4 |
| Q9JHR7 | Ide | 117.70 | 164.33 | 4.91 | 5 | 5 | 5 |
| Q64674 | Srm | 33.97 | 164.19 | 16.89 | 4 | 4 | 4 |
| P35700 | Prdx1 | 22.16 | 163.46 | 23.62 | 3 | 5 | 5 |
| Q8BH04 | Pck2 | 70.48 | 161.69 | 11.88 | 6 | 6 | 6 |
| Q64737 | Gart | 107.44 | 161.38 | 4.36 | 2 | 2 | 3 |
| Q9QZD9 | Eif3i | 36.44 | 160.66 | 7.08 | 2 | 2 | 3 |
| Q9WTM5 | Ruvbl2 | 51.08 | 160.49 | 11.02 | 4 | 4 | 4 |
| P18760 | Cfl1 | 18.55 | 160.29 | 33.73 | 4 | 4 | 4 |
| P26151 | Fcgr1 | 44.86 | 159.98 | 9.41 | 3 | 3 | 4 |
| O55131 | Sept7 | 50.52 | 159.66 | 9.4 | 4 | 4 | 5 |
| Q9WVJ2 | Psmd13 | 42.78 | 158.93 | 11.7 | 4 | 4 | 4 |
| Q3U9G9 | Lbr | 71.39 | 158.45 | 6.07 | 3 | 3 | 3 |
| P51174 | Acadl | 47.88 | 158.19 | 11.16 | 4 | 4 | 4 |
| P14824 | Anxa6 | 75.84 | 157.93 | 9.21 | 5 | 5 | 5 |
| Q8BML9 | Qars | 87.62 | 157.31 | 8.77 | 5 | 5 | 5 |
| O70194 | Eif3d | 63.95 | 156.72 | 8.39 | 4 | 4 | 4 |
| Q9DCL9 | Paics | 46.98 | 156.65 | 10.59 | 5 | 5 | 5 |
| Q62351 | Tfrc | 85.68 | 156.11 | 4.46 | 3 | 3 | 3 |
| Q8VDJ3 | Hdlbp | 141.66 | 155.41 | 3.71 | 4 | 4 | 4 |
| Q61781 | Krt14 | 52.83 | 155.40 | 9.09 | 2 | 5 | 5 |
| Q8VDM4 | Psmd2 | 100.14 | 154.83 | 5.62 | 4 | 4 | 4 |
| Q9CY64 | Blvra | 33.50 | 153.86 | 12.54 | 3 | 3 | 3 |
| Q9QYJ0 | Dnaja2 | 45.72 | 153.00 | 7.52 | 3 | 3 | 3 |
| O09167 | Rpl21 | 18.55 | 152.73 | 16.25 | 2 | 2 | 4 |
| Q8BG05 | Hnrnpa3 | 39.63 | 152.49 | 14.25 | 3 | 4 | 5 |
| P62192 | Psmc1 | 49.15 | 151.96 | 8.86 | 2 | 3 | 3 |
| P19253 | Rpl13a | 23.45 | 151.11 | 22.17 | 6 | 6 | 6 |
| P62918 | Rpl8 | 28.01 | 150.81 | 15.95 | 4 | 4 | 4 |
| P62270 | Rps18 | 17.71 | 149.73 | 23.68 | 4 | 4 | 4 |
| Q9Z1Q5 | Clic1 | 27.00 | 149.65 | 21.16 | 4 | 4 | 4 |
| Q8C0C7 | Farsa | 57.56 | 148.53 | 7.09 | 3 | 3 | 3 |
| P62751 | Rpl23a | 17.68 | 148.38 | 21.79 | 3 | 3 | 3 |
| Q9WUA2 | Farsb | 65.66 | 148.10 | 7.64 | 4 | 5 | 5 |
| Q9JKB3 | Ybx3 | 38.79 | 147.77 | 8.31 | 1 | 2 | 2 |
| Q9CZX8 | Rps19 | 16.08 | 143.76 | 20 | 3 | 3 | 3 |
| Q9CPY7 | Lap3 | 56.11 | 142.32 | 7.51 | 4 | 4 | 4 |
| O89053 | Coro1a | 50.96 | 139.99 | 10.41 | 4 | 4 | 4 |
| P29758 | Oat | 48.32 | 136.21 | 10.02 | 4 | 4 | 4 |
| Q9D883 | U2af1 | 27.80 | 135.70 | 10.46 | 2 | 2 | 2 |
| Q9CWK8 | Snx2 | 58.43 | 135.68 | 8.86 | 3 | 4 | 4 |
| Q9WV55 | Vapa | 27.84 | 135.26 | 13.65 | 3 | 3 | 3 |
| Q14C51 | Ptcd3 | 77.75 | 135.18 | 5.99 | 4 | 4 | 4 |
| P62281 | Rps11 | 18.42 | 133.79 | 20.89 | 4 | 4 | 4 |
| Q1HFZ0 | Nsun2 | 85.40 | 133.66 | 4.76 | 3 | 3 | 3 |
| Q8K297 | Colgalt1 | 71.02 | 133.46 | 8.91 | 6 | 6 | 6 |
| Q99JY0 | Hadhb | 51.35 | 132.32 | 5.89 | 3 | 3 | 3 |
| O09131 | Gsto1 | 27.48 | 131.92 | 17.92 | 4 | 4 | 4 |
| P62082 | Rps7 | 22.11 | 131.70 | 18.56 | 4 | 4 | 4 |
| Q01768 | Nme2 | 17.35 | 130.11 | 28.29 | 3 | 3 | 4 |
| Q9DB20 | Atp5po | 23.35 | 130.02 | 15.02 | 3 | 3 | 3 |
| Q9Z0X1 | Aifm1 | 66.72 | 129.43 | 5.56 | 3 | 3 | 3 |
| P62245 | Rps15a | 14.83 | 128.79 | 30.77 | 4 | 4 | 4 |
| P62334 | Psmc6 | 44.15 | 127.69 | 6.68 | 2 | 2 | 2 |
| Q9QUJ7 | Acsl4 | 79.03 | 127.30 | 5.63 | 2 | 2 | 2 |
| Q9D8U8 | Snx5 | 46.77 | 126.49 | 9.65 | 3 | 4 | 4 |
| O35658 | C1qbp | 30.99 | 126.46 | 9.71 | 3 | 3 | 3 |
| O35286 | Dhx15 | 90.95 | 126.22 | 4.4 | 3 | 3 | 3 |
| P30416 | Fkbp4 | 51.54 | 125.98 | 8.3 | 3 | 3 | 3 |
| Q3THS6 | Mat2a | 43.66 | 125.88 | 7.59 | 2 | 2 | 2 |
| P61358 | Rpl27 | 15.79 | 124.71 | 19.85 | 3 | 3 | 3 |
| O54734 | Ddost | 49.00 | 122.99 | 8.39 | 4 | 4 | 4 |
| P62301 | Rps13 | 17.21 | 122.29 | 24.5 | 3 | 3 | 3 |
| Q99P91 | Gpnmb | 63.64 | 121.86 | 6.62 | 2 | 2 | 3 |
| Q9JII6 | Akr1a1 | 36.56 | 121.40 | 9.54 | 3 | 3 | 3 |
| P29391 | Ftl1 | 20.79 | 121.07 | 13.11 | 2 | 2 | 2 |
| Q9QUR6 | Prep | 80.70 | 120.69 | 5.49 | 3 | 3 | 3 |
| P57780 | Actn4 | 104.91 | 120.48 | 2.74 | 1 | 2 | 2 |
| P17918 | Pcna | 28.77 | 119.85 | 13.41 | 4 | 4 | 4 |
| P61164 | Actr1a | 42.59 | 119.71 | 10.11 | 2 | 2 | 2 |
| Q9JLJ2 | Aldh9a1 | 53.48 | 119.39 | 6.88 | 3 | 3 | 3 |
| Q8BG32 | Psmd11 | 47.41 | 119.36 | 6.4 | 2 | 2 | 2 |
| Q9CY58 | Serbp1 | 44.69 | 119.06 | 6.63 | 2 | 2 | 2 |
| P09528 | Fth1 | 21.05 | 119.01 | 19.23 | 3 | 3 | 3 |
| Q9JIK5 | Ddx21 | 93.49 | 116.82 | 4.94 | 4 | 4 | 4 |
| Q8BGD9 | Eif4b | 68.80 | 116.45 | 6.06 | 3 | 3 | 3 |
| P19157 | Gstp1 | 23.59 | 116.42 | 12.86 | 2 | 2 | 2 |
| Q8CIG8 | Prmt5 | 72.63 | 116.21 | 3.92 | 2 | 2 | 2 |
| Q3U7R1 | Esyt1 | 121.48 | 115.38 | 2.47 | 2 | 2 | 3 |
| Q61024 | Asns | 64.24 | 115.10 | 5.53 | 3 | 3 | 3 |
| Q3TXS7 | Psmd1 | 105.66 | 114.73 | 3.99 | 3 | 3 | 3 |
| P14685 | Psmd3 | 60.68 | 113.74 | 6.23 | 3 | 3 | 3 |
| Q7TPR4 | Actn1 | 103.00 | 113.59 | 4.6 | 2 | 3 | 3 |
| Q99020 | Hnrnpab | 30.81 | 113.34 | 13.68 | 3 | 3 | 3 |
| Q9DBT5 | Ampd2 | 91.97 | 113.27 | 3.76 | 3 | 3 | 3 |
| O08756 | Hsd17b10 | 27.40 | 112.56 | 7.66 | 1 | 1 | 2 |
| Q9R0P3 | Esd | 31.30 | 112.22 | 15.6 | 3 | 3 | 3 |
| P0CG49 | Ubb | 34.35 | 111.75 | 11.8 | 1 | 1 | 3 |
| Q9D6R2 | Idh3a | 39.61 | 110.76 | 8.74 | 3 | 3 | 3 |
| Q8K1B8 | Fermt3 | 75.59 | 109.16 | 5.26 | 3 | 3 | 3 |
| Q8BKC5 | Ipo5 | 123.51 | 109.14 | 3.92 | 3 | 3 | 3 |
| Q5XJY5 | Arcn1 | 57.19 | 108.98 | 5.87 | 3 | 3 | 3 |
| Q60668 | Hnrnpd | 38.33 | 108.38 | 6.2 | 2 | 2 | 2 |
| Q9Z1Z2 | Strap | 38.42 | 108.34 | 6.86 | 2 | 2 | 2 |
| P32921 | Wars | 54.32 | 107.04 | 7.28 | 3 | 3 | 3 |
| P14148 | Rpl7 | 31.40 | 106.69 | 9.63 | 3 | 3 | 3 |
| Q99L45 | Eif2s2 | 38.07 | 105.90 | 9.67 | 3 | 3 | 3 |
| O08807 | Prdx4 | 31.03 | 105.90 | 15.33 | 2 | 4 | 4 |
| P35486 | Pdha1 | 43.20 | 105.81 | 5.64 | 2 | 2 | 2 |
| Q61990 | Pcbp2 | 38.20 | 105.65 | 8.84 | 2 | 3 | 3 |
| Q9Z1D1 | Eif3g | 35.62 | 105.42 | 8.44 | 2 | 2 | 2 |
| Q9EQU5 | Set | 33.36 | 104.86 | 10.03 | 3 | 3 | 3 |
| Q61081 | Cdc37 | 44.56 | 104.63 | 11.61 | 4 | 4 | 4 |
| O88844 | Idh1 | 46.64 | 104.32 | 5.8 | 2 | 2 | 3 |
| P35979 | Rpl12 | 17.79 | 103.65 | 24.24 | 3 | 3 | 3 |
| Q920E5 | Fdps | 40.56 | 103.12 | 7.93 | 2 | 2 | 2 |
| P62264 | Rps14 | 16.26 | 101.65 | 15.89 | 2 | 2 | 2 |
| Q8BK72 | Mrps27 | 47.75 | 100.52 | 5.54 | 2 | 2 | 2 |
| Q3TRM8 | Hk3 | 100.04 | 99.75 | 4.01 | 2 | 3 | 3 |
| P54822 | Adsl | 54.83 | 99.64 | 6.2 | 2 | 2 | 2 |
| Q8BSY0 | Asph | 82.99 | 99.47 | 3.51 | 2 | 2 | 2 |
| Q8K411 | Pitrm1 | 117.30 | 99.31 | 4.83 | 3 | 3 | 3 |
| P25206 | Mcm3 | 91.49 | 99.20 | 5.42 | 4 | 4 | 4 |
| P47757 | Capzb | 31.33 | 99.17 | 10.11 | 2 | 2 | 2 |
| P14115 | Rpl27a | 16.60 | 98.58 | 14.19 | 2 | 2 | 2 |
| Q9WV32 | Arpc1b | 41.04 | 98.54 | 8.33 | 2 | 2 | 2 |
| Q6P1F6 | Ppp2r2a | 51.66 | 97.92 | 5.59 | 2 | 2 | 2 |
| Q68FL6 | Mars | 101.37 | 97.06 | 2 | 2 | 2 | 2 |
| Q91WJ8 | Fubp1 | 68.50 | 96.85 | 3.99 | 1 | 2 | 2 |
| Q80WJ7 | Mtdh | 63.81 | 96.81 | 3.63 | 2 | 2 | 2 |
| Q9WVK4 | Ehd1 | 60.56 | 96.35 | 5.99 | 3 | 3 | 3 |
| Q8BIJ6 | Iars2 | 112.73 | 96.33 | 1.68 | 1 | 1 | 1 |
| A0A0B4J1G0 | Fcgr4 | 28.38 | 96.28 | 9.24 | 2 | 2 | 2 |
| P62835 | Rap1a | 20.97 | 96.11 | 11.96 | 2 | 2 | 2 |
| Q9DCN2 | Cyb5r3 | 34.11 | 95.97 | 3.99 | 1 | 1 | 2 |
| Q9WV80 | Snx1 | 58.92 | 95.62 | 4.98 | 1 | 2 | 2 |
| Q9CQC6 | Bzw1 | 48.01 | 93.98 | 5.97 | 3 | 3 | 3 |
| P62267 | Rps23 | 15.80 | 93.85 | 15.38 | 2 | 2 | 2 |
| O70435 | Psma3 | 28.39 | 93.59 | 11.37 | 3 | 3 | 3 |
| Q99KK7 | Dpp3 | 82.85 | 93.20 | 3.52 | 3 | 3 | 3 |
| Q9QUM9 | Psma6 | 27.35 | 93.15 | 9.35 | 2 | 2 | 2 |
| Q9CPR4 | Rpl17 | 21.41 | 93.14 | 14.13 | 2 | 2 | 2 |
| P60335 | Pcbp1 | 37.47 | 92.96 | 8.43 | 2 | 3 | 3 |
| P28650 | Adssl1 | 50.22 | 92.79 | 4.81 | 2 | 2 | 2 |
| P42208 | Sept2 | 41.50 | 92.47 | 4.43 | 1 | 1 | 1 |
| Q9WUK2 | Eif4h | 27.32 | 92.15 | 5.24 | 1 | 1 | 1 |
| O08788 | Dctn1 | 141.59 | 91.81 | 0.94 | 1 | 1 | 1 |
| P70168 | Kpnb1 | 97.12 | 91.43 | 4.22 | 3 | 3 | 3 |
| Q8JZV7 | Amdhd2 | 43.47 | 91.07 | 6.11 | 2 | 2 | 2 |
| E9PVA8 | Gcn1 | 292.83 | 91.05 | 1.5 | 3 | 3 | 3 |
| Q60865 | Caprin1 | 78.12 | 91.00 | 3.25 | 2 | 2 | 2 |
| Q07813 | Bax | 21.38 | 89.77 | 6.77 | 1 | 1 | 1 |
| Q8BVY0 | Rsl1d1 | 50.39 | 89.32 | 6.19 | 2 | 2 | 2 |
| Q6IFX2 | Krt42 | 50.10 | 88.38 | 5.53 | 1 | 3 | 3 |
| Q9JMD0 | Znf207 | 52.76 | 88.18 | 2.42 | 1 | 1 | 1 |
| Q9CYG7 | Tomm34 | 34.26 | 87.50 | 7.77 | 2 | 2 | 2 |
| P62806 | Hist1h4a | 11.36 | 87.50 | 21.36 | 2 | 2 | 2 |
| Q8BP67 | Rpl24 | 17.77 | 87.27 | 10.83 | 2 | 2 | 2 |
| P26369 | U2af2 | 53.48 | 87.21 | 3.79 | 2 | 2 | 2 |
| P97461 | Rps5 | 22.88 | 86.95 | 12.75 | 2 | 2 | 2 |
| Q9CXW3 | Cacybp | 26.49 | 86.79 | 13.54 | 3 | 3 | 3 |
| Q9CXW4 | Rpl11 | 20.24 | 86.70 | 11.8 | 2 | 2 | 2 |
| Q62433 | Ndrg1 | 42.98 | 86.63 | 3.55 | 1 | 1 | 1 |
| P60867 | Rps20 | 13.36 | 86.36 | 9.24 | 1 | 1 | 1 |
| Q6A0A9 | FAM120A | 121.57 | 86.00 | 1.53 | 1 | 1 | 1 |
| Q64514 | Tpp2 | 139.79 | 85.90 | 2.54 | 3 | 3 | 3 |
| P11103 | Parp1 | 113.03 | 85.87 | 1.28 | 1 | 1 | 1 |
| P61161 | Actr2 | 44.73 | 85.57 | 8.12 | 3 | 3 | 3 |
| Q61171 | Prdx2 | 21.77 | 85.16 | 13.64 | 2 | 2 | 2 |
| Q9CQA3 | Sdhb | 31.79 | 84.51 | 7.09 | 2 | 2 | 2 |
| Q9CXW2 | Mrps22 | 41.17 | 84.27 | 6.41 | 2 | 2 | 2 |
| Q9R1P4 | Psma1 | 29.53 | 83.86 | 7.98 | 2 | 2 | 2 |
| Q9CQT1 | Mri1 | 39.39 | 83.37 | 6.78 | 2 | 2 | 2 |
| P54775 | Psmc4 | 47.38 | 82.96 | 6.46 | 2 | 2 | 3 |
| P20108 | Prdx3 | 28.11 | 82.00 | 7 | 2 | 2 | 2 |
| P61255 | Rpl26 | 17.25 | 81.73 | 12.41 | 2 | 2 | 2 |
| Q9CQM9 | Glrx3 | 37.75 | 81.65 | 7.42 | 2 | 2 | 2 |
| P35279 | Rab6a | 23.57 | 80.99 | 10.58 | 1 | 2 | 2 |
| P24668 | M6pr | 31.15 | 80.94 | 5.04 | 1 | 1 | 1 |
| P07724 | Alb | 68.65 | 80.75 | 3.13 | 2 | 2 | 2 |
| Q9CZ13 | Uqcrc1 | 52.82 | 80.47 | 6.04 | 2 | 2 | 2 |
| Q3V3R1 | Mthfd1l | 105.66 | 80.18 | 2.05 | 2 | 2 | 2 |
| Q07417 | Acads | 44.86 | 79.50 | 2.91 | 1 | 1 | 1 |
| Q9D7G0 | Prps1 | 34.81 | 79.48 | 11.64 | 3 | 3 | 3 |
| Q921H8 | Acaa1a | 43.93 | 79.30 | 8.96 | 2 | 2 | 2 |
| P62852 | Rps25 | 13.73 | 79.08 | 15.2 | 2 | 2 | 2 |
| Q62093 | Srsf2 | 25.46 | 78.92 | 6.79 | 2 | 2 | 2 |
| Q6P542 | Abcf1 | 94.89 | 78.89 | 2.99 | 2 | 2 | 2 |
| Q9Z2U0 | Psma7 | 27.84 | 78.77 | 9.27 | 2 | 2 | 2 |
| Q80X85 | Mrps7 | 28.04 | 78.56 | 4.96 | 1 | 1 | 1 |
| P59999 | Arpc4 | 19.65 | 78.52 | 11.31 | 2 | 2 | 2 |
| Q9CZ44 | Nsfl1c | 40.69 | 78.47 | 4.32 | 1 | 1 | 1 |
| Q9WUU7 | Ctsz | 33.97 | 78.35 | 3.27 | 1 | 1 | 1 |
| P35922 | Fmr1 | 68.95 | 78.20 | 2.28 | 1 | 2 | 2 |
| Q91V41 | Rab14 | 23.88 | 78.07 | 9.3 | 1 | 2 | 2 |
| P63028 | Tpt1 | 19.45 | 77.98 | 13.95 | 2 | 2 | 2 |
| Q8R0X7 | Sgpl1 | 63.64 | 77.27 | 2.64 | 1 | 1 | 1 |
| Q921M3 | Sf3b3 | 135.46 | 76.55 | 1.56 | 2 | 2 | 2 |
| O35593 | Psmd14 | 34.55 | 76.30 | 6.45 | 2 | 2 | 2 |
| Q8CHW4 | Eif2b5 | 80.04 | 75.43 | 3.07 | 2 | 2 | 2 |
| Q9CVB6 | Arpc2 | 34.34 | 75.21 | 6 | 2 | 2 | 2 |
| P63037 | Dnaja1 | 44.84 | 75.19 | 4.79 | 2 | 2 | 2 |
| O08553 | Dpysl2 | 62.24 | 75.00 | 2.97 | 2 | 2 | 2 |
| Q99J09 | Wdr77 | 36.92 | 74.27 | 13.45 | 3 | 3 | 3 |
| Q3THK7 | Gmps | 76.67 | 73.98 | 3.32 | 2 | 2 | 2 |
| Q9D051 | Pdhb | 38.91 | 73.67 | 6.69 | 2 | 2 | 2 |
| Q61543 | Glg1 | 133.65 | 73.49 | 1.87 | 2 | 2 | 2 |
| Q9Z127 | Slc7a5 | 55.84 | 73.39 | 2.73 | 1 | 1 | 1 |
| Q9D8W5 | Psmd12 | 52.86 | 73.10 | 2.41 | 1 | 1 | 1 |
| P04117 | Fabp4 | 14.64 | 73.04 | 9.09 | 1 | 1 | 1 |
| Q69ZN7 | Myof | 233.18 | 72.93 | 1.66 | 2 | 2 | 2 |
| Q7TNV0 | Dek | 43.13 | 72.45 | 3.42 | 1 | 1 | 1 |
| Q99JY9 | Actr3 | 47.33 | 72.22 | 5.98 | 2 | 2 | 2 |
| Q6P5F9 | Xpo1 | 123.01 | 72.18 | 2.24 | 2 | 2 | 2 |
| Q61768 | Kif5b | 109.48 | 71.50 | 1.45 | 1 | 1 | 1 |
| O54984 | Asna1 | 38.80 | 71.49 | 5.17 | 2 | 2 | 2 |
| E9Q5C9 | Nolc1 | 73.65 | 71.35 | 1.85 | 1 | 1 | 1 |
| P53986 | Slc16a1 | 53.23 | 71.28 | 2.64 | 1 | 1 | 1 |
| Q9D0R2 | Tars | 83.30 | 70.78 | 3.05 | 2 | 2 | 2 |
| P97429 | Anxa4 | 35.89 | 70.49 | 4.7 | 1 | 1 | 1 |
| P20152 | Vim | 53.66 | 70.15 | 2.15 | 1 | 1 | 1 |
| P41105 | Rpl28 | 15.72 | 70.12 | 13.14 | 2 | 2 | 2 |
| P67984 | Rpl22 | 14.75 | 70.12 | 10.16 | 1 | 1 | 1 |
| Q6PDI5 | Ecpas | 203.57 | 69.70 | 0.82 | 1 | 1 | 1 |
| O08528 | Hk2 | 102.47 | 69.65 | 2.51 | 1 | 2 | 2 |
| O89112 | Lancl1 | 45.31 | 69.17 | 3.01 | 1 | 1 | 1 |
| Q9JIK9 | Mrps34 | 25.81 | 69.14 | 10.55 | 2 | 2 | 2 |
| Q9QZE5 | Copg1 | 97.45 | 68.89 | 2.06 | 2 | 2 | 2 |
| Q3TWW8 | Srsf6 | 39.00 | 68.67 | 5.31 | 2 | 2 | 2 |
| Q99KQ4 | Nampt | 55.41 | 68.50 | 3.26 | 2 | 2 | 2 |
| Q9Z2I8 | Suclg2 | 46.81 | 68.25 | 7.16 | 2 | 2 | 2 |
| Q8VEM8 | Slc25a3 | 39.61 | 68.15 | 6.44 | 2 | 2 | 2 |
| Q9JIF7 | Copb1 | 107.00 | 67.68 | 1.15 | 1 | 1 | 1 |
| Q99LF4 | Rtcb | 55.21 | 67.39 | 5.94 | 2 | 2 | 2 |
| Q8CFE2 | Hpf1 | 39.27 | 67.31 | 3.18 | 1 | 1 | 1 |
| P62849 | Rps24 | 15.41 | 67.11 | 17.29 | 2 | 2 | 2 |
| P62717 | Rpl18a | 20.72 | 66.77 | 5.68 | 1 | 1 | 2 |
| Q91YI0 | Asl | 51.71 | 66.64 | 1.94 | 1 | 1 | 1 |
| P10853 | Hist1h2bf | 13.93 | 65.95 | 15.87 | 2 | 2 | 2 |
| Q7TNC4 | Luc7l2 | 46.55 | 65.75 | 3.83 | 1 | 1 | 1 |
| Q9EPU0 | Upf1 | 123.89 | 65.62 | 1.51 | 1 | 1 | 1 |
| Q9D1R9 | Rpl34 | 13.28 | 65.51 | 7.69 | 1 | 1 | 1 |
| Q921F2 | Tardbp | 44.52 | 65.47 | 5.07 | 2 | 2 | 2 |
| P10639 | Txn | 11.67 | 65.42 | 8.57 | 1 | 1 | 1 |
| P08003 | Pdia4 | 71.94 | 65.39 | 5.64 | 2 | 2 | 2 |
| Q62376 | Snrnp70 | 51.96 | 65.22 | 4.02 | 2 | 2 | 2 |
| P34884 | Mif | 12.50 | 64.58 | 9.57 | 1 | 1 | 1 |
| Q99KP6 | Prpf19 | 55.20 | 64.56 | 4.76 | 2 | 2 | 2 |
| Q922Q8 | Lrrc59 | 34.86 | 64.38 | 6.51 | 2 | 2 | 2 |
| Q9Z2U1 | Psma5 | 26.39 | 64.32 | 8.3 | 2 | 2 | 2 |
| P39749 | Fen1 | 42.29 | 64.08 | 5.82 | 2 | 2 | 2 |
| Q8R050 | Gspt1 | 68.58 | 63.97 | 5.82 | 2 | 2 | 2 |
| Q6P1B1 | Xpnpep1 | 69.55 | 63.88 | 2.89 | 2 | 2 | 2 |
| Q9ER72 | Cars | 94.80 | 63.85 | 2.65 | 2 | 2 | 2 |
| P18242 | Ctsd | 44.92 | 63.25 | 2.44 | 1 | 1 | 1 |
| Q9D0F3 | Lman1 | 57.75 | 63.16 | 2.13 | 1 | 1 | 1 |
| Q78ZA7 | Nap1l4 | 42.65 | 63.09 | 2.67 | 1 | 1 | 2 |
| Q6VN19 | Ranbp10 | 67.15 | 62.71 | 1.61 | 1 | 1 | 1 |
| Q8BMK4 | Ckap4 | 63.65 | 62.51 | 2.78 | 1 | 1 | 1 |
| Q8VCM8 | Ncln | 62.87 | 62.27 | 1.95 | 1 | 1 | 1 |
| Q8CG48 | Smc2 | 134.16 | 62.10 | 2.02 | 2 | 2 | 2 |
| Q9JM76 | Arpc3 | 20.51 | 62.00 | 6.18 | 1 | 1 | 1 |
| P10107 | Anxa1 | 38.71 | 61.87 | 3.18 | 1 | 1 | 1 |
| Q9D938 | Tmem160 | 19.57 | 61.87 | 9.04 | 2 | 2 | 2 |
| P23506 | Pcmt1 | 24.62 | 61.67 | 4.85 | 1 | 1 | 1 |
| Q9WTP6 | Ak2 | 26.45 | 61.31 | 6.69 | 1 | 1 | 1 |
| Q9ER88 | Dap3 | 44.67 | 60.86 | 3.84 | 1 | 1 | 1 |
| Q9Z1G3 | Atp6v1c1 | 43.86 | 60.66 | 4.97 | 2 | 2 | 2 |
| Q9D662 | Sec23b | 86.38 | 60.08 | 1.69 | 1 | 1 | 1 |
| P62715 | Ppp2cb | 35.55 | 60.04 | 3.56 | 1 | 1 | 1 |
| Q8CIN4 | Pak2 | 57.89 | 59.93 | 3.63 | 2 | 2 | 2 |
| Q9CRC9 | Gnpda2 | 31.06 | 59.78 | 4.35 | 1 | 1 | 1 |
| O88342 | Wdr1 | 66.36 | 58.84 | 2.48 | 2 | 2 | 2 |
| Q99JI4 | Psmd6 | 45.51 | 58.51 | 3.86 | 1 | 1 | 1 |
| P02301 | H3f3c | 15.31 | 58.43 | 5.15 | 1 | 1 | 1 |
| Q9CPP0 | Npm3 | 19.01 | 58.34 | 9.14 | 1 | 1 | 1 |
| Q9Z2N8 | Actl6a | 47.42 | 58.01 | 3.03 | 1 | 1 | 1 |
| Q9CX34 | Sugt1 | 38.14 | 57.70 | 3.57 | 1 | 1 | 1 |
| Q8VCT3 | Rnpep | 72.37 | 57.67 | 1.38 | 1 | 1 | 1 |
| Q5SF07 | Igf2bp2 | 65.54 | 57.56 | 4.39 | 2 | 2 | 2 |
| Q8QZR5 | Gpt | 55.11 | 57.42 | 2.62 | 1 | 1 | 1 |
| Q9R0N0 | Galk1 | 42.27 | 57.03 | 3.06 | 1 | 1 | 1 |
| Q9CXL3 | --- | 22.15 | 56.95 | 4.62 | 1 | 1 | 1 |
| Q8CCS6 | Pabpn1 | 32.28 | 56.85 | 5.3 | 1 | 1 | 1 |
| Q6PHZ2 | Camk2d | 56.33 | 56.71 | 2 | 1 | 1 | 1 |
| P08030 | Aprt | 19.71 | 56.32 | 5.56 | 1 | 1 | 1 |
| P20612 | Gnat1 | 39.94 | 56.16 | 3.14 | 1 | 1 | 1 |
| Q61655 | Ddx19a | 53.90 | 56.02 | 2.3 | 1 | 1 | 1 |
| Q8CGP6 | Hist1h2ah | 13.94 | 56.02 | 14.84 | 1 | 1 | 2 |
| Q9D7N3 | Mrps9 | 44.90 | 55.95 | 2.82 | 1 | 1 | 1 |
| Q9JHK5 | Plek | 39.88 | 55.61 | 4 | 1 | 1 | 1 |
| Q9CR16 | Ppid | 40.72 | 55.57 | 2.43 | 1 | 1 | 1 |
| P70699 | Gaa | 106.18 | 55.34 | 1.05 | 1 | 1 | 1 |
| Q8R3N1 | Nop14 | 98.71 | 55.15 | 1.28 | 1 | 1 | 1 |
| P46664 | Adss | 49.99 | 55.11 | 2.41 | 1 | 1 | 1 |
| P02340 | Tp53 | 43.43 | 54.91 | 3.33 | 1 | 1 | 1 |
| P09671 | Sod2 | 24.59 | 54.27 | 6.31 | 1 | 1 | 1 |
| Q9JIF0 | Prmt1 | 42.41 | 54.11 | 2.96 | 1 | 1 | 1 |
| P32233 | Drg1 | 40.49 | 54.05 | 3 | 1 | 1 | 1 |
| O09061 | Psmb1 | 26.36 | 53.57 | 5.83 | 1 | 1 | 1 |
| P52293 | Kpna2 | 57.89 | 53.45 | 2.84 | 1 | 1 | 1 |
| Q9WVA4 | Tagln2 | 22.38 | 53.43 | 5.53 | 1 | 1 | 1 |
| P22437 | Ptgs1 | 69.00 | 53.40 | 2.49 | 1 | 1 | 1 |
| Q9D1D4 | Tmed10 | 24.89 | 53.10 | 4.11 | 1 | 1 | 2 |
| Q8CG47 | Smc4 | 146.80 | 52.98 | 0.78 | 1 | 1 | 1 |
| P51410 | Rpl9 | 21.87 | 52.55 | 5.21 | 1 | 1 | 1 |
| Q8BVE3 | Atp6v1h | 55.82 | 52.39 | 2.69 | 1 | 1 | 1 |
| P11438 | Lamp1 | 43.84 | 52.08 | 2.71 | 1 | 1 | 1 |
| Q9R1P3 | Psmb2 | 22.89 | 51.86 | 5.47 | 1 | 1 | 1 |
| G5E829 | Atp2b1 | 134.66 | 51.68 | 1.31 | 1 | 1 | 1 |
| P63276 | Rps17 | 15.51 | 51.50 | 7.41 | 1 | 1 | 1 |
| P10852 | Slc3a2 | 58.30 | 51.49 | 1.9 | 1 | 1 | 1 |
| Q91VD9 | Ndufs1 | 79.73 | 51.45 | 1.24 | 1 | 1 | 1 |
| P63087 | Ppp1cc | 36.96 | 51.40 | 4.33 | 1 | 1 | 1 |
| Q61166 | Mapre1 | 30.00 | 51.23 | 2.99 | 1 | 1 | 1 |
| B9EJ86 | Osbpl8 | 101.21 | 51.18 | 1.24 | 1 | 1 | 1 |
| P29387 | Gnb4 | 37.36 | 51.09 | 2.94 | 1 | 1 | 1 |
| Q9CY27 | Tecr | 36.07 | 51.03 | 3.57 | 1 | 1 | 1 |
| Q99NB9 | Sf3b1 | 145.72 | 50.98 | 0.61 | 1 | 1 | 1 |
| P49722 | Psma2 | 25.91 | 50.91 | 5.98 | 1 | 1 | 1 |
| P60766 | Cdc42 | 21.25 | 50.88 | 8.9 | 1 | 1 | 1 |
| Q61599 | Arhgdib | 22.84 | 50.82 | 8 | 1 | 1 | 1 |
| Q5SUR0 | Pfas | 144.54 | 50.76 | 0.82 | 1 | 1 | 1 |
| Q06185 | Atp5me | 8.23 | 50.67 | 16.9 | 1 | 1 | 1 |
| Q8CIH5 | Plcg2 | 147.50 | 50.66 | 0.79 | 1 | 1 | 1 |
| Q99LD4 | Gps1 | 53.41 | 50.54 | 3.18 | 1 | 1 | 1 |
| P21107 | Tpm3 | 32.97 | 50.33 | 4.56 | 1 | 1 | 1 |
| Q924Z4 | Cers2 | 45.00 | 50.33 | 4.21 | 1 | 1 | 1 |
| P17439 | Gba | 57.59 | 50.00 | 3.11 | 1 | 1 | 1 |
| P17742 | Ppia | 17.96 | 49.42 | 5.49 | 1 | 1 | 1 |
| P70195 | Psmb7 | 29.87 | 49.39 | 3.61 | 1 | 1 | 1 |
| O70310 | Nmt1 | 56.85 | 49.38 | 2.82 | 1 | 1 | 1 |
| P49615 | Cdk5 | 33.27 | 49.29 | 2.74 | 1 | 1 | 1 |
| Q99KK9 | Hars2 | 56.95 | 49.19 | 1.98 | 1 | 1 | 1 |
| O08795 | Prkcsh | 58.76 | 48.94 | 1.92 | 1 | 1 | 1 |
| O55029 | Copb2 | 102.38 | 48.86 | 1.1 | 1 | 1 | 1 |
| Q9ES97 | Rtn3 | 103.81 | 48.76 | 1.14 | 1 | 1 | 1 |
| P59325 | Eif5 | 48.94 | 48.73 | 3.5 | 1 | 1 | 1 |
| Q9JLZ6 | Hic2 | 66.72 | 48.61 | 1.62 | 1 | 1 | 1 |
| P47791 | Gsr | 53.63 | 48.57 | 2.4 | 1 | 1 | 1 |
| Q61792 | Lasp1 | 29.98 | 47.75 | 3.42 | 1 | 1 | 1 |
| P51150 | Rab7a | 23.47 | 47.53 | 4.83 | 1 | 1 | 1 |
| Q9JKV1 | Adrm1 | 42.03 | 47.08 | 3.93 | 1 | 1 | 1 |
| O08709 | Prdx6 | 24.85 | 47.00 | 6.25 | 1 | 1 | 1 |
| Q9DBG3 | Ap2b1 | 104.52 | 46.77 | 2.24 | 1 | 1 | 1 |
| Q3UGC7 | Eif3j1 | 29.33 | 46.66 | 4.21 | 1 | 1 | 1 |
| P46460 | Nsf | 82.56 | 46.56 | 1.48 | 1 | 1 | 1 |
| Q8CDN6 | Txnl1 | 32.22 | 46.54 | 5.19 | 1 | 1 | 1 |
| P57759 | Erp29 | 28.81 | 46.36 | 4.58 | 1 | 1 | 1 |
| Q9CX56 | Psmd8 | 39.90 | 45.74 | 2.27 | 1 | 1 | 1 |
| Q61029 | Tmpo | 50.34 | 45.64 | 2.65 | 1 | 1 | 1 |
| O35841 | Api5 | 56.75 | 45.64 | 3.17 | 1 | 1 | 1 |
| Q8K183 | Pdxk | 34.99 | 45.60 | 6.73 | 1 | 1 | 1 |
| Q9CQQ7 | Atp5pb | 28.93 | 45.48 | 3.13 | 1 | 1 | 1 |
| Q99N69 | Lpxn | 43.45 | 45.42 | 5.18 | 1 | 1 | 1 |
| Q6ZQM8 | Ugt1a7c | 59.72 | 45.24 | 3.01 | 1 | 1 | 1 |
| Q8C1A5 | Thop1 | 77.98 | 45.16 | 1.46 | 1 | 1 | 1 |
| Q62446 | Fkbp3 | 25.13 | 45.11 | 4.91 | 1 | 1 | 1 |
| Q64G17 | Anp32c | 14.06 | 45.10 | 6.5 | 1 | 1 | 1 |
| Q60737 | Csnk2a1 | 45.10 | 45.02 | 3.84 | 1 | 1 | 1 |
| Q9Z1Z0 | Uso1 | 106.92 | 44.69 | 1.04 | 1 | 1 | 1 |
| Q9WV54 | Asah1 | 44.64 | 44.57 | 3.05 | 1 | 1 | 1 |
| Q8BK64 | Ahsa1 | 38.09 | 44.54 | 2.96 | 1 | 1 | 1 |
| Q07133 | Hist1h1t | 21.53 | 44.37 | 5.29 | 1 | 1 | 1 |
| Q64433 | Hspe1 | 10.96 | 44.27 | 13.73 | 1 | 1 | 1 |
| Q6P9R2 | Oxsr1 | 58.18 | 44.07 | 2.85 | 1 | 1 | 1 |
| Q8BQM4 | Heatr3 | 74.26 | 44.00 | 1.18 | 1 | 1 | 1 |
| P51863 | Atp6v0d1 | 40.28 | 43.91 | 3.13 | 1 | 1 | 1 |
| Q3UPL0 | Sec31a | 133.49 | 43.56 | 1.71 | 1 | 1 | 1 |
| Q9D7B1 | Dus2 | 55.29 | 43.55 | 1.42 | 1 | 1 | 1 |
| Q05920 | Pc | 129.60 | 43.47 | 1.1 | 1 | 1 | 1 |
| P56135 | Atp5mf | 10.34 | 43.39 | 13.64 | 1 | 1 | 1 |
| P70290 | Mpp1 | 52.19 | 43.31 | 1.72 | 1 | 1 | 1 |
| P14131 | Rps16 | 16.44 | 43.28 | 6.85 | 1 | 1 | 1 |
| P97855 | G3bp1 | 51.80 | 43.12 | 2.37 | 1 | 1 | 1 |
| Q8JZR0 | Acsl5 | 76.16 | 43.03 | 1.61 | 1 | 1 | 1 |
| Q8R010 | Aimp2 | 35.36 | 42.73 | 2.81 | 1 | 1 | 1 |
| P13439 | Umps | 52.26 | 42.45 | 2.08 | 1 | 1 | 1 |
| Q80WQ2 | Vac14 | 87.99 | 42.06 | 1.41 | 1 | 1 | 1 |
| Q8CI11 | Gnl3 | 60.75 | 41.94 | 2.04 | 1 | 1 | 1 |
| P97822 | Anp32e | 29.60 | 41.85 | 4.62 | 1 | 1 | 1 |
| Q8VH51 | Rbm39 | 59.37 | 41.66 | 2.83 | 1 | 1 | 1 |
| P63325 | Rps10 | 18.90 | 41.43 | 5.45 | 1 | 1 | 1 |
| Q9CW03 | Smc3 | 141.47 | 41.23 | 0.99 | 1 | 1 | 1 |
| P84104 | Srsf3 | 19.32 | 41.15 | 5.49 | 1 | 1 | 1 |
| Q9R0Q3 | Tmed2 | 22.69 | 41.12 | 4.48 | 1 | 1 | 1 |
| P51660 | Hsd17b4 | 79.43 | 41.02 | 1.77 | 1 | 1 | 1 |
| Q8VI75 | Ipo4 | 119.20 | 41.02 | 1.02 | 1 | 1 | 1 |
| P47753 | Capza1 | 32.92 | 41.00 | 6.29 | 1 | 1 | 1 |
| Q80UM3 | Naa15 | 100.90 | 40.91 | 1.04 | 1 | 1 | 1 |
| Q8R016 | Blmh | 52.48 | 40.88 | 1.54 | 1 | 1 | 1 |
| Q9Z2I9 | Sucla2 | 50.08 | 40.77 | 2.16 | 1 | 1 | 1 |
| P14576 | Srp54 | 55.68 | 40.55 | 2.58 | 1 | 1 | 1 |
| O54988 | Slk | 141.37 | 40.18 | 0.73 | 1 | 1 | 1 |
| Q91YP2 | Nln | 80.38 | 40.18 | 1.42 | 1 | 1 | 1 |
| P47964 | Rpl36 | 12.21 | 40.07 | 8.57 | 1 | 1 | 1 |
| P62962 | Pfn1 | 14.95 | 39.79 | 11.43 | 1 | 1 | 1 |
| P12265 | Gusb | 74.15 | 39.77 | 1.08 | 1 | 1 | 1 |
| Q8K1M6 | Dnm1l | 82.61 | 39.68 | 1.21 | 1 | 1 | 1 |
| Q99LE6 | Abcf2 | 71.74 | 39.27 | 1.75 | 1 | 1 | 1 |
| Q61206 | Pafah1b2 | 25.57 | 39.05 | 3.93 | 1 | 1 | 1 |
| P14901 | Hmox1 | 32.91 | 38.63 | 4.15 | 1 | 1 | 1 |
| O88487 | Dync1i2 | 68.35 | 38.62 | 1.63 | 1 | 1 | 1 |
| Q61335 | Bcap31 | 27.94 | 38.56 | 4.08 | 1 | 1 | 1 |
| P21956 | Mfge8 | 51.21 | 38.53 | 2.38 | 1 | 1 | 1 |
| Q9D0F9 | Pgm1 | 61.38 | 38.10 | 2.31 | 1 | 1 | 1 |
| Q8C2Q3 | Rbm14 | 69.41 | 38.03 | 2.24 | 1 | 1 | 1 |
| P07091 | S100a4 | 11.71 | 38.01 | 16.83 | 1 | 1 | 1 |
| Q62422 | Ostf1 | 23.77 | 37.88 | 5.58 | 1 | 1 | 1 |
| P01027 | C3 | 186.37 | 37.79 | 0.6 | 1 | 1 | 1 |
| O88653 | Lamtor3 | 13.54 | 37.78 | 5.65 | 1 | 1 | 1 |
| P31230 | Aimp1 | 33.98 | 37.67 | 3.87 | 1 | 1 | 1 |
| Q05816 | Fabp5 | 15.13 | 37.54 | 6.67 | 1 | 1 | 1 |
| P31996 | Cd68 | 34.80 | 37.50 | 2.76 | 1 | 1 | 1 |
| O35609 | Scamp3 | 38.43 | 37.41 | 4.58 | 1 | 1 | 1 |
| Q6ZWV7 | Rpl35 | 14.54 | 37.36 | 8.13 | 1 | 1 | 1 |
| Q99N87 | Mrps5 | 48.18 | 37.04 | 3.24 | 1 | 1 | 1 |
| A2A9C3 | Szt2 | 377.39 | 37.04 | 0.17 | 1 | 1 | 1 |
| Q9QXA5 | Lsm4 | 15.07 | 36.98 | 5.11 | 1 | 1 | 1 |
| Q64727 | Vcl | 116.64 | 36.95 | 1.13 | 1 | 1 | 1 |
| Q61749 | Eif2b4 | 57.59 | 36.89 | 2.1 | 1 | 1 | 1 |
| Q9EPK7 | Xpo7 | 123.73 | 36.88 | 1.01 | 1 | 1 | 1 |
| Q9ESJ0 | Xpo4 | 129.90 | 36.75 | 1.04 | 1 | 1 | 1 |
| Q91ZJ5 | Ugp2 | 56.94 | 36.67 | 1.97 | 1 | 1 | 1 |
| Q61510 | Trim25 | 71.68 | 36.66 | 1.89 | 1 | 1 | 1 |
| P22892 | Ap1g1 | 91.29 | 36.62 | 1.09 | 1 | 1 | 1 |
| P97371 | Psme1 | 28.65 | 36.55 | 5.62 | 1 | 1 | 1 |
| Q9R190 | Mta2 | 74.98 | 36.48 | 1.2 | 1 | 1 | 1 |
| O35643 | Ap1b1 | 103.87 | 36.45 | 2.23 | 1 | 1 | 1 |
| Q62448 | Eif4g2 | 102.04 | 36.40 | 1.1 | 1 | 1 | 1 |
| Q99ME9 | Gtpbp4 | 74.07 | 36.36 | 1.1 | 1 | 1 | 1 |
| Q64521 | Gpd2 | 80.90 | 36.26 | 2.34 | 1 | 1 | 1 |
| P97379 | G3bp2 | 54.05 | 35.75 | 1.66 | 1 | 1 | 1 |
| Q9D880 | Timm50 | 39.75 | 35.61 | 3.12 | 1 | 1 | 1 |
| Q61334 | Bcap29 | 27.95 | 35.49 | 3.75 | 1 | 1 | 1 |
| Q9CY62 | Rnf181 | 19.09 | 34.97 | 4.85 | 1 | 1 | 1 |
| Q9Z2W0 | Dnpep | 52.17 | 34.79 | 2.96 | 1 | 1 | 1 |
| P55264 | Adk | 40.12 | 34.39 | 1.66 | 1 | 1 | 1 |
| Q4FZF3 | Ddx49 | 54.06 | 34.06 | 1.25 | 1 | 1 | 1 |
| Q7TQH0 | Atxn2l | 110.58 | 33.88 | 0.86 | 1 | 1 | 1 |
| Q8K4Z5 | Sf3a1 | 88.49 | 33.84 | 1.64 | 1 | 1 | 1 |
| Q99MR6 | Srrt | 100.39 | 33.23 | 1.14 | 1 | 1 | 1 |
| P17809 | Slc2a1 | 53.95 | 33.14 | 1.63 | 1 | 1 | 1 |
| Q07076 | Anxa7 | 49.89 | 31.60 | 1.94 | 1 | 1 | 1 |
| Q8BNV1 | Trmt2a | 63.26 | 31.41 | 1.74 | 1 | 1 | 1 |
| B2RY56 | Rbm25 | 99.49 | 30.43 | 1.79 | 1 | 1 | 1 |
| Q99PL5 | Rrbp1 | 172.78 | 30.03 | 0.44 | 1 | 1 | 1 |
| P19096 | Fasn | 272.26 | 29.13 | 0.76 | 1 | 1 | 1 |
| P47915 | Rpl29 | 17.58 | 27.55 | 6.88 | 1 | 1 | 1 |
| Q80UG5 | Sept9 | 65.53 | 27.51 | 1.72 | 1 | 1 | 1 |
|  |  |  |  |  |  |  |  |
| IgG IP |  |  |  |  |  |  |  |
| **Accession** | **Gene names** | **MW [kDa]** | **Protein score** | **Sequence coverage (%)** | **# Unique Peptides** | **# Peptides** | **# PSMs** |
| P63038 | Hspd1 | 60.92 | 583.63 | 18.67 | 9 | 9 | 14 |
| Q61781 | Krt14 | 52.83 | 382.52 | 21.49 | 1 | 9 | 11 |
| P63017 | Hspa8 | 70.83 | 321.34 | 12.85 | 6 | 7 | 7 |
| P02535 | Krt10 | 57.74 | 291.99 | 10.7 | 4 | 6 | 7 |
| Q922U2 | Krt5 | 61.73 | 280.77 | 10.86 | 3 | 8 | 9 |
| Q9Z2K1 | Krt16 | 51.57 | 280.40 | 11.73 | 1 | 5 | 8 |
| Q9QWL7 | Krt17 | 48.13 | 258.07 | 15.01 | 1 | 7 | 8 |
| Q03265 | Atp5f1a | 59.72 | 253.30 | 10.85 | 4 | 4 | 4 |
| Q6IFX2 | Krt42 | 50.10 | 247.33 | 17.48 | 2 | 7 | 7 |
| P50446 | Krt6a | 59.30 | 243.51 | 11.57 | 2 | 7 | 7 |
| P38647 | Hspa9 | 73.42 | 239.25 | 9.87 | 6 | 6 | 6 |
| P16858 | Gapdh | 35.79 | 207.92 | 12.61 | 3 | 3 | 4 |
| Q8VED5 | Krt79 | 57.52 | 196.90 | 7.34 | 1 | 5 | 6 |
| P10126 | Eef1a1 | 50.08 | 196.89 | 12.55 | 5 | 5 | 6 |
| P19001 | Krt19 | 44.51 | 188.88 | 12.9 | 1 | 6 | 6 |
| Q8BGZ7 | Krt75 | 59.70 | 188.33 | 8.53 | 1 | 5 | 6 |
| P04104 | Krt1 | 65.57 | 183.24 | 6.12 | 3 | 5 | 5 |
| P56480 | Atp5f1b | 56.27 | 182.85 | 10.02 | 4 | 4 | 5 |
| Q3TTY5 | Krt2 | 70.88 | 169.67 | 5.37 | 2 | 4 | 4 |
| P99024 | Tubb5 | 49.64 | 166.38 | 7.88 | 3 | 3 | 3 |
| P11679 | Krt8 | 54.53 | 165.88 | 5.92 | 1 | 4 | 5 |
| P68373 | Tuba1c | 49.88 | 149.39 | 11.14 | 1 | 4 | 4 |
| P05784 | Krt18 | 47.51 | 142.37 | 7.57 | 1 | 4 | 4 |
| P68369 | Tuba1a | 50.10 | 129.72 | 11.09 | 1 | 4 | 4 |
| P08113 | Hsp90b1 | 92.42 | 126.37 | 5.24 | 3 | 3 | 3 |
| P07356 | Anxa2 | 38.65 | 123.39 | 11.8 | 3 | 3 | 3 |
| P09103 | P4hb | 57.02 | 114.71 | 8.25 | 3 | 3 | 3 |
| Q8K2B3 | Sdha | 72.54 | 111.13 | 2.11 | 1 | 1 | 1 |
| P20152 | Vim | 53.66 | 110.48 | 4.51 | 2 | 2 | 2 |
| P08249 | Mdh2 | 35.59 | 109.58 | 8.88 | 2 | 2 | 2 |
| P20029 | Hspa5 | 72.38 | 106.62 | 3.82 | 1 | 2 | 2 |
| Q8VEK3 | Hnrnpu | 87.86 | 103.82 | 1.88 | 1 | 1 | 1 |
| Q64310 | Surf4 | 30.36 | 96.33 | 5.95 | 1 | 1 | 1 |
| P11499 | Hsp90ab1 | 83.23 | 89.63 | 3.59 | 2 | 2 | 2 |
| P62270 | Rps18 | 17.71 | 85.54 | 12.5 | 2 | 2 | 2 |
| Q9EQK5 | Mvp | 95.87 | 81.34 | 2.09 | 2 | 2 | 2 |
| A0A0B4J1G0 | Fcgr4 | 28.38 | 80.06 | 9.24 | 2 | 2 | 2 |
| P52480 | Pkm | 57.81 | 79.70 | 5.46 | 2 | 2 | 2 |
| P60710 | Actb | 41.71 | 71.72 | 8.53 | 2 | 2 | 2 |
| Q8BMS1 | Hadha | 82.62 | 69.64 | 5.9 | 2 | 2 | 2 |
| P62806 | Hist1h4a | 11.36 | 62.59 | 9.71 | 1 | 1 | 1 |
| P07724 | Alb | 68.65 | 61.48 | 2.14 | 1 | 1 | 1 |
| P22437 | Ptgs1 | 69.00 | 61.17 | 2.49 | 1 | 1 | 1 |
| P07901 | Hsp90aa1 | 84.73 | 59.09 | 1.64 | 1 | 1 | 1 |
| Q61753 | Phgdh | 56.55 | 58.25 | 2.81 | 1 | 1 | 1 |
| Q922R8 | Pdia6 | 48.07 | 57.93 | 3.18 | 1 | 1 | 1 |
| P62918 | Rpl8 | 28.01 | 55.06 | 6.23 | 1 | 1 | 1 |
| Q68FD5 | Cltc | 191.43 | 54.75 | 0.54 | 1 | 1 | 1 |
| P47963 | Rpl13 | 24.29 | 53.65 | 5.21 | 1 | 1 | 1 |
| P17182 | Eno1 | 47.11 | 53.55 | 1.38 | 1 | 1 | 1 |
| P00342 | Ldhc | 35.89 | 51.65 | 2.71 | 1 | 1 | 1 |
| P14148 | Rpl7 | 31.40 | 51.23 | 4.07 | 1 | 1 | 1 |
| Q07133 | Hist1h1t | 21.53 | 50.55 | 5.29 | 1 | 1 | 1 |
| Q9CR57 | Rpl14 | 23.55 | 47.97 | 5.99 | 1 | 1 | 1 |
| Q8BHN3 | Ganab | 106.84 | 47.15 | 1.27 | 1 | 1 | 1 |
| P14824 | Anxa6 | 75.84 | 46.68 | 1.49 | 1 | 1 | 1 |
| P62908 | Rps3 | 26.66 | 45.76 | 3.7 | 1 | 1 | 1 |
| P14211 | Calr | 47.96 | 44.16 | 2.64 | 1 | 1 | 1 |
| Q9QXA5 | Lsm4 | 15.07 | 41.50 | 5.11 | 1 | 1 | 1 |
| P29341 | Pabpc1 | 70.63 | 37.76 | 1.57 | 1 | 1 | 1 |
|  |  |  |  |  |  |  |  |
| Identified protein | |  | Cytoplasmic granules-related | |  |  |  |
| Q8VDD5 | Myh9 |  | Eif4a1 |  |  |  |  |
| P09405 | Ncl |  | Eif3a |  |  |  |  |
| P09411 | Pgk1 |  | Ruvbl1 |  |  |  |  |
| P68372 | Tubb4b |  | Eif3c |  |  |  |  |
| P26041 | Msn |  | Eif3b |  |  |  |  |
| P40142 | Tkt |  | Eif3l |  |  |  |  |
| Q99MR8 | Mccc1 |  | Snd1 |  |  |  |  |
| P05064 | Aldoa |  | Eif3e |  |  |  |  |
| P58252 | Eef2 |  | Eif4g1 |  |  |  |  |
| Q9Z1Q9 | Vars |  | Eif3f |  |  |  |  |
| Q3ULD5 | Mccc2 |  | Rack1 |  |  |  |  |
| P80318 | Cct3 |  | Eif3h |  |  |  |  |
| Q02053 | Uba1 |  | Eif3i |  |  |  |  |
| P60843 | Eif4a1 |  | Eif3d |  |  |  |  |
| Q61316 | Hspa4 |  | Ddx21 |  |  |  |  |
| P42932 | Cct8 |  | Eif4b |  |  |  |  |
| P23116 | Eif3a |  | Eif3g |  |  |  |  |
| Q9D8E6 | Rpl4 |  | Eif4h |  |  |  |  |
| Q61233 | Lcp1 |  | Caprin1 |  |  |  |  |
| P27773 | Pdia3 |  | FAM120A |  |  |  |  |
| P80314 | Cct2 |  | Upf1 |  |  |  |  |
| P26039 | Tln1 |  | Igf2bp2 |  |  |  |  |
| Q8VDN2 | Atp1a1 |  | Eif3j1 |  |  |  |  |
| P06151 | Ldha |  | G3bp1 |  |  |  |  |
| P61979 | Hnrnpk |  | Eif4g2 |  |  |  |  |
| Q9JKF1 | Iqgap1 |  | G3bp2 |  |  |  |  |
| P11983 | Tcp1 |  |  |  |  |  |  |
| Q922F4 | Tubb6 |  |  |  |  |  |  |
| Q9CZN7 | Shmt2 |  |  |  |  |  |  |
| P68033 | Actc1 |  |  |  |  |  |  |
| P29351 | Ptpn6 |  |  |  |  |  |  |
| P10649 | Gstm1 |  |  |  |  |  |  |
| P50516 | Atp6v1a |  |  |  |  |  |  |
| Q99K85 | Psat1 |  |  |  |  |  |  |
| Q8CGC7 | Eprs |  |  |  |  |  |  |
| P06745 | Gpi |  |  |  |  |  |  |
| P47738 | Aldh2 |  |  |  |  |  |  |
| P60122 | Ruvbl1 |  |  |  |  |  |  |
| Q91YQ5 | Rpn1 |  |  |  |  |  |  |
| P80315 | Cct4 |  |  |  |  |  |  |
| Q7TPV4 | Mybbp1a |  |  |  |  |  |  |
| Q922B2 | Dars |  |  |  |  |  |  |
| Q9DBJ1 | Pgam1 |  |  |  |  |  |  |
| Q8BGQ7 | Aars |  |  |  |  |  |  |
| P47911 | Rpl6 |  |  |  |  |  |  |
| Q6ZWX6 | Eif2s1 |  |  |  |  |  |  |
| O55143 | Atp2a2 |  |  |  |  |  |  |
| Q9D8N0 | Eef1g |  |  |  |  |  |  |
| P80313 | Cct7 |  |  |  |  |  |  |
| P80316 | Cct5 |  |  |  |  |  |  |
| Q8BMF4 | Dlat |  |  |  |  |  |  |
| Q8R1B4 | Eif3c |  |  |  |  |  |  |
| P05202 | Got2 |  |  |  |  |  |  |
| P05201 | Got1 |  |  |  |  |  |  |
| O08749 | Dld |  |  |  |  |  |  |
| P48036 | Anxa5 |  |  |  |  |  |  |
| Q9Z0N1 | Eif2s3x |  |  |  |  |  |  |
| Q9ERK4 | Cse1l |  |  |  |  |  |  |
| Q8JZQ9 | Eif3b |  |  |  |  |  |  |
| Q8QZY1 | Eif3l |  |  |  |  |  |  |
| P80317 | Cct6a |  |  |  |  |  |  |
| O35129 | Phb2 |  |  |  |  |  |  |
| Q9D0E1 | Hnrnpm |  |  |  |  |  |  |
| Q9WUA3 | Pfkp |  |  |  |  |  |  |
| P63101 | Ywhaz |  |  |  |  |  |  |
| Q78PY7 | Snd1 |  |  |  |  |  |  |
| P24452 | Capg |  |  |  |  |  |  |
| E9Q634 | Myo1e |  |  |  |  |  |  |
| Q8VIJ6 | Sfpq |  |  |  |  |  |  |
| P12970 | Rpl7a |  |  |  |  |  |  |
| Q6ZWN5 | Rps9 |  |  |  |  |  |  |
| Q9CWJ9 | Atic |  |  |  |  |  |  |
| P50247 | Ahcy |  |  |  |  |  |  |
| P62702 | Rps4x |  |  |  |  |  |  |
| P50580 | Pa2g4 |  |  |  |  |  |  |
| Q3TW96 | Uap1l1 |  |  |  |  |  |  |
| Q8BFR5 | Tufm |  |  |  |  |  |  |
| Q9CZD3 | Gars |  |  |  |  |  |  |
| P60229 | Eif3e |  |  |  |  |  |  |
| Q99KI0 | Aco2 |  |  |  |  |  |  |
| Q9D0I9 | Rars |  |  |  |  |  |  |
| Q9CQV8 | Ywhab |  |  |  |  |  |  |
| P47962 | Rpl5 |  |  |  |  |  |  |
| Q7TMK9 | Syncrip |  |  |  |  |  |  |
| Q8BU30 | Iars |  |  |  |  |  |  |
| Q9CZ30 | Ola1 |  |  |  |  |  |  |
| P51881 | Slc25a5 |  |  |  |  |  |  |
| Q93092 | Taldo1 |  |  |  |  |  |  |
| Q91WQ3 | Yars |  |  |  |  |  |  |
| P62754 | Rps6 |  |  |  |  |  |  |
| P62814 | Atp6v1b2 |  |  |  |  |  |  |
| Q9DBG6 | Rpn2 |  |  |  |  |  |  |
| P28658 | Atxn10 |  |  |  |  |  |  |
| Q8R180 | Ero1a |  |  |  |  |  |  |
| Q01853 | Vcp |  |  |  |  |  |  |
| Q922D8 | Mthfd1 |  |  |  |  |  |  |
| P26040 | Ezr |  |  |  |  |  |  |
| Q9D0K2 | Oxct1 |  |  |  |  |  |  |
| Q61598 | Gdi2 |  |  |  |  |  |  |
| Q76MZ3 | Ppp2r1a |  |  |  |  |  |  |
| Q6NZJ6 | Eif4g1 |  |  |  |  |  |  |
| P12382 | Pfkl |  |  |  |  |  |  |
| Q62318 | Trim28 |  |  |  |  |  |  |
| P14869 | Rplp0 |  |  |  |  |  |  |
| O35737 | Hnrnph1 |  |  |  |  |  |  |
| P62960 | Ybx1 |  |  |  |  |  |  |
| Q62167 | Ddx3x |  |  |  |  |  |  |
| P54071 | Idh2 |  |  |  |  |  |  |
| P48962 | Slc25a4 |  |  |  |  |  |  |
| Q9DCH4 | Eif3f |  |  |  |  |  |  |
| Q99L47 | St13 |  |  |  |  |  |  |
| Q501J6 | Ddx17 |  |  |  |  |  |  |
| P26638 | Sars |  |  |  |  |  |  |
| Q8BMJ2 | Lars |  |  |  |  |  |  |
| Q62465 | Vat1 |  |  |  |  |  |  |
| P46471 | Psmc2 |  |  |  |  |  |  |
| Q64337 | Sqstm1 |  |  |  |  |  |  |
| Q61656 | Ddx5 |  |  |  |  |  |  |
| P16460 | Ass1 |  |  |  |  |  |  |
| Q80X90 | Flnb |  |  |  |  |  |  |
| Q99MN1 | Kars |  |  |  |  |  |  |
| Q00612 | G6pdx |  |  |  |  |  |  |
| P14206 | Rpsa |  |  |  |  |  |  |
| Q8BTM8 | Flna |  |  |  |  |  |  |
| Q9DCD0 | Pgd |  |  |  |  |  |  |
| Q61937 | Npm1 |  |  |  |  |  |  |
| Q9CQN1 | Trap1 |  |  |  |  |  |  |
| P61982 | Ywhag |  |  |  |  |  |  |
| P57776 | Eef1d |  |  |  |  |  |  |
| Q60854 | Serpinb6 |  |  |  |  |  |  |
| P62196 | Psmc5 |  |  |  |  |  |  |
| P28352 | Apex1 |  |  |  |  |  |  |
| P68254 | Ywhaq |  |  |  |  |  |  |
| P26443 | Glud1 |  |  |  |  |  |  |
| P62259 | Ywhae |  |  |  |  |  |  |
| Q9D2G2 | Dlst |  |  |  |  |  |  |
| O35639 | Anxa3 |  |  |  |  |  |  |
| P26043 | Rdx |  |  |  |  |  |  |
| O35685 | Nudc |  |  |  |  |  |  |
| Q9EPL8 | Ipo7 |  |  |  |  |  |  |
| O88569 | Hnrnpa2b1 |  |  |  |  |  |  |
| Q6PB66 | Lrpprc |  |  |  |  |  |  |
| P32067 | Ssb |  |  |  |  |  |  |
| Q60973 | Rbbp7 |  |  |  |  |  |  |
| P27659 | Rpl3 |  |  |  |  |  |  |
| Q9D1A2 | Cndp2 |  |  |  |  |  |  |
| P16110 | Lgals3 |  |  |  |  |  |  |
| P61205 | Arf3 |  |  |  |  |  |  |
| Q60817 | Naca |  |  |  |  |  |  |
| P35980 | Rpl18 |  |  |  |  |  |  |
| P97351 | Rps3a |  |  |  |  |  |  |
| Q8VDW0 | Ddx39a |  |  |  |  |  |  |
| P62242 | Rps8 |  |  |  |  |  |  |
| P62821 | Rab1A |  |  |  |  |  |  |
| P25444 | Rps2 |  |  |  |  |  |  |
| Q9JKR6 | Hyou1 |  |  |  |  |  |  |
| Q9Z2X1 | Hnrnpf |  |  |  |  |  |  |
| Q9CZM2 | Rpl15 |  |  |  |  |  |  |
| Q8BK67 | Rcc2 |  |  |  |  |  |  |
| Q60864 | Stip1 |  |  |  |  |  |  |
| P97807 | Fh |  |  |  |  |  |  |
| Q6ZWV3 | Rpl10 |  |  |  |  |  |  |
| P37040 | Por |  |  |  |  |  |  |
| P12813 | Nr4a1 |  |  |  |  |  |  |
| Q6ZQ38 | Cand1 |  |  |  |  |  |  |
| Q8BWY3 | Etf1 |  |  |  |  |  |  |
| P62827 | Ran |  |  |  |  |  |  |
| Q8BP47 | Nars |  |  |  |  |  |  |
| P53026 | Rpl10a |  |  |  |  |  |  |
| Q99JB2 | Stoml2 |  |  |  |  |  |  |
| P45376 | Akr1b1 |  |  |  |  |  |  |
| Q8CGK3 | Lonp1 |  |  |  |  |  |  |
| Q61699 | Hsph1 |  |  |  |  |  |  |
| P24547 | Impdh2 |  |  |  |  |  |  |
| Q99PT1 | Arhgdia |  |  |  |  |  |  |
| O70251 | Eef1b |  |  |  |  |  |  |
| Q99K48 | Nono |  |  |  |  |  |  |
| Q60972 | Rbbp4 |  |  |  |  |  |  |
| Q91VI7 | Rnh1 |  |  |  |  |  |  |
| P17751 | Tpi1 |  |  |  |  |  |  |
| P54987 | Acod1 |  |  |  |  |  |  |
| Q3U1J4 | Ddb1 |  |  |  |  |  |  |
| Q60597 | Ogdh |  |  |  |  |  |  |
| Q9EQH3 | Vps35 |  |  |  |  |  |  |
| P17563 | Selenbp1 |  |  |  |  |  |  |
| P84099 | Rpl19 |  |  |  |  |  |  |
| Q9JMA1 | Usp14 |  |  |  |  |  |  |
| O88685 | Psmc3 |  |  |  |  |  |  |
| P17225 | Ptbp1 |  |  |  |  |  |  |
| P53395 | Dbt |  |  |  |  |  |  |
| Q9Z110 | Aldh18a1 |  |  |  |  |  |  |
| Q3U0V1 | Khsrp |  |  |  |  |  |  |
| P97311 | Mcm6 |  |  |  |  |  |  |
| P08730 | Krt13 |  |  |  |  |  |  |
| P17710 | Hk1 |  |  |  |  |  |  |
| P40124 | Cap1 |  |  |  |  |  |  |
| Q91VR2 | Atp5f1c |  |  |  |  |  |  |
| P68040 | Rack1 |  |  |  |  |  |  |
| P35564 | Canx |  |  |  |  |  |  |
| P13020 | Gsn |  |  |  |  |  |  |
| P67778 | Phb |  |  |  |  |  |  |
| Q9CZU6 | Cs |  |  |  |  |  |  |
| Q91V92 | Acly |  |  |  |  |  |  |
| Q91WK2 | Eif3h |  |  |  |  |  |  |
| P82343 | Renbp |  |  |  |  |  |  |
| Q9JHU9 | Isyna1 |  |  |  |  |  |  |
| Q9JHR7 | Ide |  |  |  |  |  |  |
| Q64674 | Srm |  |  |  |  |  |  |
| P35700 | Prdx1 |  |  |  |  |  |  |
| Q8BH04 | Pck2 |  |  |  |  |  |  |
| Q64737 | Gart |  |  |  |  |  |  |
| Q9QZD9 | Eif3i |  |  |  |  |  |  |
| Q9WTM5 | Ruvbl2 |  |  |  |  |  |  |
| P18760 | Cfl1 |  |  |  |  |  |  |
| P26151 | Fcgr1 |  |  |  |  |  |  |
| O55131 | Sept7 |  |  |  |  |  |  |
| Q9WVJ2 | Psmd13 |  |  |  |  |  |  |
| Q3U9G9 | Lbr |  |  |  |  |  |  |
| P51174 | Acadl |  |  |  |  |  |  |
| Q8BML9 | Qars |  |  |  |  |  |  |
| O70194 | Eif3d |  |  |  |  |  |  |
| Q9DCL9 | Paics |  |  |  |  |  |  |
| Q62351 | Tfrc |  |  |  |  |  |  |
| Q8VDJ3 | Hdlbp |  |  |  |  |  |  |
| Q8VDM4 | Psmd2 |  |  |  |  |  |  |
| Q9CY64 | Blvra |  |  |  |  |  |  |
| Q9QYJ0 | Dnaja2 |  |  |  |  |  |  |
| O09167 | Rpl21 |  |  |  |  |  |  |
| Q8BG05 | Hnrnpa3 |  |  |  |  |  |  |
| P62192 | Psmc1 |  |  |  |  |  |  |
| P19253 | Rpl13a |  |  |  |  |  |  |
| Q9Z1Q5 | Clic1 |  |  |  |  |  |  |
| Q8C0C7 | Farsa |  |  |  |  |  |  |
| P62751 | Rpl23a |  |  |  |  |  |  |
| Q9WUA2 | Farsb |  |  |  |  |  |  |
| Q9JKB3 | Ybx3 |  |  |  |  |  |  |
| Q9CZX8 | Rps19 |  |  |  |  |  |  |
| Q9CPY7 | Lap3 |  |  |  |  |  |  |
| O89053 | Coro1a |  |  |  |  |  |  |
| P29758 | Oat |  |  |  |  |  |  |
| Q9D883 | U2af1 |  |  |  |  |  |  |
| Q9CWK8 | Snx2 |  |  |  |  |  |  |
| Q9WV55 | Vapa |  |  |  |  |  |  |
| Q14C51 | Ptcd3 |  |  |  |  |  |  |
| P62281 | Rps11 |  |  |  |  |  |  |
| Q1HFZ0 | Nsun2 |  |  |  |  |  |  |
| Q8K297 | Colgalt1 |  |  |  |  |  |  |
| Q99JY0 | Hadhb |  |  |  |  |  |  |
| O09131 | Gsto1 |  |  |  |  |  |  |
| P62082 | Rps7 |  |  |  |  |  |  |
| Q01768 | Nme2 |  |  |  |  |  |  |
| Q9DB20 | Atp5po |  |  |  |  |  |  |
| Q9Z0X1 | Aifm1 |  |  |  |  |  |  |
| P62245 | Rps15a |  |  |  |  |  |  |
| P62334 | Psmc6 |  |  |  |  |  |  |
| Q9QUJ7 | Acsl4 |  |  |  |  |  |  |
| Q9D8U8 | Snx5 |  |  |  |  |  |  |
| O35658 | C1qbp |  |  |  |  |  |  |
| O35286 | Dhx15 |  |  |  |  |  |  |
| P30416 | Fkbp4 |  |  |  |  |  |  |
| Q3THS6 | Mat2a |  |  |  |  |  |  |
| P61358 | Rpl27 |  |  |  |  |  |  |
| O54734 | Ddost |  |  |  |  |  |  |
| P62301 | Rps13 |  |  |  |  |  |  |
| Q99P91 | Gpnmb |  |  |  |  |  |  |
| Q9JII6 | Akr1a1 |  |  |  |  |  |  |
| P29391 | Ftl1 |  |  |  |  |  |  |
| Q9QUR6 | Prep |  |  |  |  |  |  |
| P57780 | Actn4 |  |  |  |  |  |  |
| P17918 | Pcna |  |  |  |  |  |  |
| P61164 | Actr1a |  |  |  |  |  |  |
| Q9JLJ2 | Aldh9a1 |  |  |  |  |  |  |
| Q8BG32 | Psmd11 |  |  |  |  |  |  |
| Q9CY58 | Serbp1 |  |  |  |  |  |  |
| P09528 | Fth1 |  |  |  |  |  |  |
| Q9JIK5 | Ddx21 |  |  |  |  |  |  |
| Q8BGD9 | Eif4b |  |  |  |  |  |  |
| P19157 | Gstp1 |  |  |  |  |  |  |
| Q8CIG8 | Prmt5 |  |  |  |  |  |  |
| Q3U7R1 | Esyt1 |  |  |  |  |  |  |
| Q61024 | Asns |  |  |  |  |  |  |
| Q3TXS7 | Psmd1 |  |  |  |  |  |  |
| P14685 | Psmd3 |  |  |  |  |  |  |
| Q7TPR4 | Actn1 |  |  |  |  |  |  |
| Q99020 | Hnrnpab |  |  |  |  |  |  |
| Q9DBT5 | Ampd2 |  |  |  |  |  |  |
| O08756 | Hsd17b10 |  |  |  |  |  |  |
| Q9R0P3 | Esd |  |  |  |  |  |  |
| P0CG49 | Ubb |  |  |  |  |  |  |
| Q9D6R2 | Idh3a |  |  |  |  |  |  |
| Q8K1B8 | Fermt3 |  |  |  |  |  |  |
| Q8BKC5 | Ipo5 |  |  |  |  |  |  |
| Q5XJY5 | Arcn1 |  |  |  |  |  |  |
| Q60668 | Hnrnpd |  |  |  |  |  |  |
| Q9Z1Z2 | Strap |  |  |  |  |  |  |
| P32921 | Wars |  |  |  |  |  |  |
| Q99L45 | Eif2s2 |  |  |  |  |  |  |
| O08807 | Prdx4 |  |  |  |  |  |  |
| P35486 | Pdha1 |  |  |  |  |  |  |
| Q61990 | Pcbp2 |  |  |  |  |  |  |
| Q9Z1D1 | Eif3g |  |  |  |  |  |  |
| Q9EQU5 | Set |  |  |  |  |  |  |
| Q61081 | Cdc37 |  |  |  |  |  |  |
| O88844 | Idh1 |  |  |  |  |  |  |
| P35979 | Rpl12 |  |  |  |  |  |  |
| Q920E5 | Fdps |  |  |  |  |  |  |
| P62264 | Rps14 |  |  |  |  |  |  |
| Q8BK72 | Mrps27 |  |  |  |  |  |  |
| Q3TRM8 | Hk3 |  |  |  |  |  |  |
| P54822 | Adsl |  |  |  |  |  |  |
| Q8BSY0 | Asph |  |  |  |  |  |  |
| Q8K411 | Pitrm1 |  |  |  |  |  |  |
| P25206 | Mcm3 |  |  |  |  |  |  |
| P47757 | Capzb |  |  |  |  |  |  |
| P14115 | Rpl27a |  |  |  |  |  |  |
| Q9WV32 | Arpc1b |  |  |  |  |  |  |
| Q6P1F6 | Ppp2r2a |  |  |  |  |  |  |
| Q68FL6 | Mars |  |  |  |  |  |  |
| Q91WJ8 | Fubp1 |  |  |  |  |  |  |
| Q80WJ7 | Mtdh |  |  |  |  |  |  |
| Q9WVK4 | Ehd1 |  |  |  |  |  |  |
| Q8BIJ6 | Iars2 |  |  |  |  |  |  |
| P62835 | Rap1a |  |  |  |  |  |  |
| Q9DCN2 | Cyb5r3 |  |  |  |  |  |  |
| Q9WV80 | Snx1 |  |  |  |  |  |  |
| Q9CQC6 | Bzw1 |  |  |  |  |  |  |
| P62267 | Rps23 |  |  |  |  |  |  |
| O70435 | Psma3 |  |  |  |  |  |  |
| Q99KK7 | Dpp3 |  |  |  |  |  |  |
| Q9QUM9 | Psma6 |  |  |  |  |  |  |
| Q9CPR4 | Rpl17 |  |  |  |  |  |  |
| P60335 | Pcbp1 |  |  |  |  |  |  |
| P28650 | Adssl1 |  |  |  |  |  |  |
| P42208 | Sept2 |  |  |  |  |  |  |
| Q9WUK2 | Eif4h |  |  |  |  |  |  |
| O08788 | Dctn1 |  |  |  |  |  |  |
| P70168 | Kpnb1 |  |  |  |  |  |  |
| Q8JZV7 | Amdhd2 |  |  |  |  |  |  |
| E9PVA8 | Gcn1 |  |  |  |  |  |  |
| Q60865 | Caprin1 |  |  |  |  |  |  |
| Q07813 | Bax |  |  |  |  |  |  |
| Q8BVY0 | Rsl1d1 |  |  |  |  |  |  |
| Q9JMD0 | Znf207 |  |  |  |  |  |  |
| Q9CYG7 | Tomm34 |  |  |  |  |  |  |
| Q8BP67 | Rpl24 |  |  |  |  |  |  |
| P26369 | U2af2 |  |  |  |  |  |  |
| P97461 | Rps5 |  |  |  |  |  |  |
| Q9CXW3 | Cacybp |  |  |  |  |  |  |
| Q9CXW4 | Rpl11 |  |  |  |  |  |  |
| Q62433 | Ndrg1 |  |  |  |  |  |  |
| P60867 | Rps20 |  |  |  |  |  |  |
| Q6A0A9 | FAM120A |  |  |  |  |  |  |
| Q64514 | Tpp2 |  |  |  |  |  |  |
| P11103 | Parp1 |  |  |  |  |  |  |
| P61161 | Actr2 |  |  |  |  |  |  |
| Q61171 | Prdx2 |  |  |  |  |  |  |
| Q9CQA3 | Sdhb |  |  |  |  |  |  |
| Q9CXW2 | Mrps22 |  |  |  |  |  |  |
| Q9R1P4 | Psma1 |  |  |  |  |  |  |
| Q9CQT1 | Mri1 |  |  |  |  |  |  |
| P54775 | Psmc4 |  |  |  |  |  |  |
| P20108 | Prdx3 |  |  |  |  |  |  |
| P61255 | Rpl26 |  |  |  |  |  |  |
| Q9CQM9 | Glrx3 |  |  |  |  |  |  |
| P35279 | Rab6a |  |  |  |  |  |  |
| P24668 | M6pr |  |  |  |  |  |  |
| Q9CZ13 | Uqcrc1 |  |  |  |  |  |  |
| Q3V3R1 | Mthfd1l |  |  |  |  |  |  |
| Q07417 | Acads |  |  |  |  |  |  |
| Q9D7G0 | Prps1 |  |  |  |  |  |  |
| Q921H8 | Acaa1a |  |  |  |  |  |  |
| P62852 | Rps25 |  |  |  |  |  |  |
| Q62093 | Srsf2 |  |  |  |  |  |  |
| Q6P542 | Abcf1 |  |  |  |  |  |  |
| Q9Z2U0 | Psma7 |  |  |  |  |  |  |
| Q80X85 | Mrps7 |  |  |  |  |  |  |
| P59999 | Arpc4 |  |  |  |  |  |  |
| Q9CZ44 | Nsfl1c |  |  |  |  |  |  |
| Q9WUU7 | Ctsz |  |  |  |  |  |  |
| P35922 | Fmr1 |  |  |  |  |  |  |
| Q91V41 | Rab14 |  |  |  |  |  |  |
| P63028 | Tpt1 |  |  |  |  |  |  |
| Q8R0X7 | Sgpl1 |  |  |  |  |  |  |
| Q921M3 | Sf3b3 |  |  |  |  |  |  |
| O35593 | Psmd14 |  |  |  |  |  |  |
| Q8CHW4 | Eif2b5 |  |  |  |  |  |  |
| Q9CVB6 | Arpc2 |  |  |  |  |  |  |
| P63037 | Dnaja1 |  |  |  |  |  |  |
| O08553 | Dpysl2 |  |  |  |  |  |  |
| Q99J09 | Wdr77 |  |  |  |  |  |  |
| Q3THK7 | Gmps |  |  |  |  |  |  |
| Q9D051 | Pdhb |  |  |  |  |  |  |
| Q61543 | Glg1 |  |  |  |  |  |  |
| Q9Z127 | Slc7a5 |  |  |  |  |  |  |
| Q9D8W5 | Psmd12 |  |  |  |  |  |  |
| P04117 | Fabp4 |  |  |  |  |  |  |
| Q69ZN7 | Myof |  |  |  |  |  |  |
| Q7TNV0 | Dek |  |  |  |  |  |  |
| Q99JY9 | Actr3 |  |  |  |  |  |  |
| Q6P5F9 | Xpo1 |  |  |  |  |  |  |
| Q61768 | Kif5b |  |  |  |  |  |  |
| O54984 | Asna1 |  |  |  |  |  |  |
| E9Q5C9 | Nolc1 |  |  |  |  |  |  |
| P53986 | Slc16a1 |  |  |  |  |  |  |
| Q9D0R2 | Tars |  |  |  |  |  |  |
| P97429 | Anxa4 |  |  |  |  |  |  |
| P41105 | Rpl28 |  |  |  |  |  |  |
| P67984 | Rpl22 |  |  |  |  |  |  |
| Q6PDI5 | Ecpas |  |  |  |  |  |  |
| O08528 | Hk2 |  |  |  |  |  |  |
| O89112 | Lancl1 |  |  |  |  |  |  |
| Q9JIK9 | Mrps34 |  |  |  |  |  |  |
| Q9QZE5 | Copg1 |  |  |  |  |  |  |
| Q3TWW8 | Srsf6 |  |  |  |  |  |  |
| Q99KQ4 | Nampt |  |  |  |  |  |  |
| Q9Z2I8 | Suclg2 |  |  |  |  |  |  |
| Q8VEM8 | Slc25a3 |  |  |  |  |  |  |
| Q9JIF7 | Copb1 |  |  |  |  |  |  |
| Q99LF4 | Rtcb |  |  |  |  |  |  |
| Q8CFE2 | Hpf1 |  |  |  |  |  |  |
| P62849 | Rps24 |  |  |  |  |  |  |
| P62717 | Rpl18a |  |  |  |  |  |  |
| Q91YI0 | Asl |  |  |  |  |  |  |
| P10853 | Hist1h2bf |  |  |  |  |  |  |
| Q7TNC4 | Luc7l2 |  |  |  |  |  |  |
| Q9EPU0 | Upf1 |  |  |  |  |  |  |
| Q9D1R9 | Rpl34 |  |  |  |  |  |  |
| Q921F2 | Tardbp |  |  |  |  |  |  |
| P10639 | Txn |  |  |  |  |  |  |
| P08003 | Pdia4 |  |  |  |  |  |  |
| Q62376 | Snrnp70 |  |  |  |  |  |  |
| P34884 | Mif |  |  |  |  |  |  |
| Q99KP6 | Prpf19 |  |  |  |  |  |  |
| Q922Q8 | Lrrc59 |  |  |  |  |  |  |
| Q9Z2U1 | Psma5 |  |  |  |  |  |  |
| P39749 | Fen1 |  |  |  |  |  |  |
| Q8R050 | Gspt1 |  |  |  |  |  |  |
| Q6P1B1 | Xpnpep1 |  |  |  |  |  |  |
| Q9ER72 | Cars |  |  |  |  |  |  |
| P18242 | Ctsd |  |  |  |  |  |  |
| Q9D0F3 | Lman1 |  |  |  |  |  |  |
| Q78ZA7 | Nap1l4 |  |  |  |  |  |  |
| Q6VN19 | Ranbp10 |  |  |  |  |  |  |
| Q8BMK4 | Ckap4 |  |  |  |  |  |  |
| Q8VCM8 | Ncln |  |  |  |  |  |  |
| Q8CG48 | Smc2 |  |  |  |  |  |  |
| Q9JM76 | Arpc3 |  |  |  |  |  |  |
| P10107 | Anxa1 |  |  |  |  |  |  |
| Q9D938 | Tmem160 |  |  |  |  |  |  |
| P23506 | Pcmt1 |  |  |  |  |  |  |
| Q9WTP6 | Ak2 |  |  |  |  |  |  |
| Q9ER88 | Dap3 |  |  |  |  |  |  |
| Q9Z1G3 | Atp6v1c1 |  |  |  |  |  |  |
| Q9D662 | Sec23b |  |  |  |  |  |  |
| P62715 | Ppp2cb |  |  |  |  |  |  |
| Q8CIN4 | Pak2 |  |  |  |  |  |  |
| Q9CRC9 | Gnpda2 |  |  |  |  |  |  |
| O88342 | Wdr1 |  |  |  |  |  |  |
| Q99JI4 | Psmd6 |  |  |  |  |  |  |
| P02301 | H3f3c |  |  |  |  |  |  |
| Q9CPP0 | Npm3 |  |  |  |  |  |  |
| Q9Z2N8 | Actl6a |  |  |  |  |  |  |
| Q9CX34 | Sugt1 |  |  |  |  |  |  |
| Q8VCT3 | Rnpep |  |  |  |  |  |  |
| Q5SF07 | Igf2bp2 |  |  |  |  |  |  |
| Q8QZR5 | Gpt |  |  |  |  |  |  |
| Q9R0N0 | Galk1 |  |  |  |  |  |  |
| Q9CXL3 | --- |  |  |  |  |  |  |
| Q8CCS6 | Pabpn1 |  |  |  |  |  |  |
| Q6PHZ2 | Camk2d |  |  |  |  |  |  |
| P08030 | Aprt |  |  |  |  |  |  |
| P20612 | Gnat1 |  |  |  |  |  |  |
| Q61655 | Ddx19a |  |  |  |  |  |  |
| Q8CGP6 | Hist1h2ah |  |  |  |  |  |  |
| Q9D7N3 | Mrps9 |  |  |  |  |  |  |
| Q9JHK5 | Plek |  |  |  |  |  |  |
| Q9CR16 | Ppid |  |  |  |  |  |  |
| P70699 | Gaa |  |  |  |  |  |  |
| Q8R3N1 | Nop14 |  |  |  |  |  |  |
| P46664 | Adss |  |  |  |  |  |  |
| P02340 | Tp53 |  |  |  |  |  |  |
| P09671 | Sod2 |  |  |  |  |  |  |
| Q9JIF0 | Prmt1 |  |  |  |  |  |  |
| P32233 | Drg1 |  |  |  |  |  |  |
| O09061 | Psmb1 |  |  |  |  |  |  |
| P52293 | Kpna2 |  |  |  |  |  |  |
| Q9WVA4 | Tagln2 |  |  |  |  |  |  |
| Q9D1D4 | Tmed10 |  |  |  |  |  |  |
| Q8CG47 | Smc4 |  |  |  |  |  |  |
| P51410 | Rpl9 |  |  |  |  |  |  |
| Q8BVE3 | Atp6v1h |  |  |  |  |  |  |
| P11438 | Lamp1 |  |  |  |  |  |  |
| Q9R1P3 | Psmb2 |  |  |  |  |  |  |
| G5E829 | Atp2b1 |  |  |  |  |  |  |
| P63276 | Rps17 |  |  |  |  |  |  |
| P10852 | Slc3a2 |  |  |  |  |  |  |
| Q91VD9 | Ndufs1 |  |  |  |  |  |  |
| P63087 | Ppp1cc |  |  |  |  |  |  |
| Q61166 | Mapre1 |  |  |  |  |  |  |
| B9EJ86 | Osbpl8 |  |  |  |  |  |  |
| P29387 | Gnb4 |  |  |  |  |  |  |
| Q9CY27 | Tecr |  |  |  |  |  |  |
| Q99NB9 | Sf3b1 |  |  |  |  |  |  |
| P49722 | Psma2 |  |  |  |  |  |  |
| P60766 | Cdc42 |  |  |  |  |  |  |
| Q61599 | Arhgdib |  |  |  |  |  |  |
| Q5SUR0 | Pfas |  |  |  |  |  |  |
| Q06185 | Atp5me |  |  |  |  |  |  |
| Q8CIH5 | Plcg2 |  |  |  |  |  |  |
| Q99LD4 | Gps1 |  |  |  |  |  |  |
| P21107 | Tpm3 |  |  |  |  |  |  |
| Q924Z4 | Cers2 |  |  |  |  |  |  |
| P17439 | Gba |  |  |  |  |  |  |
| P17742 | Ppia |  |  |  |  |  |  |
| P70195 | Psmb7 |  |  |  |  |  |  |
| O70310 | Nmt1 |  |  |  |  |  |  |
| P49615 | Cdk5 |  |  |  |  |  |  |
| Q99KK9 | Hars2 |  |  |  |  |  |  |
| O08795 | Prkcsh |  |  |  |  |  |  |
| O55029 | Copb2 |  |  |  |  |  |  |
| Q9ES97 | Rtn3 |  |  |  |  |  |  |
| P59325 | Eif5 |  |  |  |  |  |  |
| Q9JLZ6 | Hic2 |  |  |  |  |  |  |
| P47791 | Gsr |  |  |  |  |  |  |
| Q61792 | Lasp1 |  |  |  |  |  |  |
| P51150 | Rab7a |  |  |  |  |  |  |
| Q9JKV1 | Adrm1 |  |  |  |  |  |  |
| O08709 | Prdx6 |  |  |  |  |  |  |
| Q9DBG3 | Ap2b1 |  |  |  |  |  |  |
| Q3UGC7 | Eif3j1 |  |  |  |  |  |  |
| P46460 | Nsf |  |  |  |  |  |  |
| Q8CDN6 | Txnl1 |  |  |  |  |  |  |
| P57759 | Erp29 |  |  |  |  |  |  |
| Q9CX56 | Psmd8 |  |  |  |  |  |  |
| Q61029 | Tmpo |  |  |  |  |  |  |
| O35841 | Api5 |  |  |  |  |  |  |
| Q8K183 | Pdxk |  |  |  |  |  |  |
| Q9CQQ7 | Atp5pb |  |  |  |  |  |  |
| Q99N69 | Lpxn |  |  |  |  |  |  |
| Q6ZQM8 | Ugt1a7c |  |  |  |  |  |  |
| Q8C1A5 | Thop1 |  |  |  |  |  |  |
| Q62446 | Fkbp3 |  |  |  |  |  |  |
| Q64G17 | Anp32c |  |  |  |  |  |  |
| Q60737 | Csnk2a1 |  |  |  |  |  |  |
| Q9Z1Z0 | Uso1 |  |  |  |  |  |  |
| Q9WV54 | Asah1 |  |  |  |  |  |  |
| Q8BK64 | Ahsa1 |  |  |  |  |  |  |
| Q64433 | Hspe1 |  |  |  |  |  |  |
| Q6P9R2 | Oxsr1 |  |  |  |  |  |  |
| Q8BQM4 | Heatr3 |  |  |  |  |  |  |
| P51863 | Atp6v0d1 |  |  |  |  |  |  |
| Q3UPL0 | Sec31a |  |  |  |  |  |  |
| Q9D7B1 | Dus2 |  |  |  |  |  |  |
| Q05920 | Pc |  |  |  |  |  |  |
| P56135 | Atp5mf |  |  |  |  |  |  |
| P70290 | Mpp1 |  |  |  |  |  |  |
| P14131 | Rps16 |  |  |  |  |  |  |
| P97855 | G3bp1 |  |  |  |  |  |  |
| Q8JZR0 | Acsl5 |  |  |  |  |  |  |
| Q8R010 | Aimp2 |  |  |  |  |  |  |
| P13439 | Umps |  |  |  |  |  |  |
| Q80WQ2 | Vac14 |  |  |  |  |  |  |
| Q8CI11 | Gnl3 |  |  |  |  |  |  |
| P97822 | Anp32e |  |  |  |  |  |  |
| Q8VH51 | Rbm39 |  |  |  |  |  |  |
| P63325 | Rps10 |  |  |  |  |  |  |
| Q9CW03 | Smc3 |  |  |  |  |  |  |
| P84104 | Srsf3 |  |  |  |  |  |  |
| Q9R0Q3 | Tmed2 |  |  |  |  |  |  |
| P51660 | Hsd17b4 |  |  |  |  |  |  |
| Q8VI75 | Ipo4 |  |  |  |  |  |  |
| P47753 | Capza1 |  |  |  |  |  |  |
| Q80UM3 | Naa15 |  |  |  |  |  |  |
| Q8R016 | Blmh |  |  |  |  |  |  |
| Q9Z2I9 | Sucla2 |  |  |  |  |  |  |
| P14576 | Srp54 |  |  |  |  |  |  |
| O54988 | Slk |  |  |  |  |  |  |
| Q91YP2 | Nln |  |  |  |  |  |  |
| P47964 | Rpl36 |  |  |  |  |  |  |
| P62962 | Pfn1 |  |  |  |  |  |  |
| P12265 | Gusb |  |  |  |  |  |  |
| Q8K1M6 | Dnm1l |  |  |  |  |  |  |
| Q99LE6 | Abcf2 |  |  |  |  |  |  |
| Q61206 | Pafah1b2 |  |  |  |  |  |  |
| P14901 | Hmox1 |  |  |  |  |  |  |
| O88487 | Dync1i2 |  |  |  |  |  |  |
| Q61335 | Bcap31 |  |  |  |  |  |  |
| P21956 | Mfge8 |  |  |  |  |  |  |
| Q9D0F9 | Pgm1 |  |  |  |  |  |  |
| Q8C2Q3 | Rbm14 |  |  |  |  |  |  |
| P07091 | S100a4 |  |  |  |  |  |  |
| Q62422 | Ostf1 |  |  |  |  |  |  |
| P01027 | C3 |  |  |  |  |  |  |
| O88653 | Lamtor3 |  |  |  |  |  |  |
| P31230 | Aimp1 |  |  |  |  |  |  |
| Q05816 | Fabp5 |  |  |  |  |  |  |
| P31996 | Cd68 |  |  |  |  |  |  |
| O35609 | Scamp3 |  |  |  |  |  |  |
| Q6ZWV7 | Rpl35 |  |  |  |  |  |  |
| Q99N87 | Mrps5 |  |  |  |  |  |  |
| A2A9C3 | Szt2 |  |  |  |  |  |  |
| Q64727 | Vcl |  |  |  |  |  |  |
| Q61749 | Eif2b4 |  |  |  |  |  |  |
| Q9EPK7 | Xpo7 |  |  |  |  |  |  |
| Q9ESJ0 | Xpo4 |  |  |  |  |  |  |
| Q91ZJ5 | Ugp2 |  |  |  |  |  |  |
| Q61510 | Trim25 |  |  |  |  |  |  |
| P22892 | Ap1g1 |  |  |  |  |  |  |
| P97371 | Psme1 |  |  |  |  |  |  |
| Q9R190 | Mta2 |  |  |  |  |  |  |
| O35643 | Ap1b1 |  |  |  |  |  |  |
| Q62448 | Eif4g2 |  |  |  |  |  |  |
| Q99ME9 | Gtpbp4 |  |  |  |  |  |  |
| Q64521 | Gpd2 |  |  |  |  |  |  |
| P97379 | G3bp2 |  |  |  |  |  |  |
| Q9D880 | Timm50 |  |  |  |  |  |  |
| Q61334 | Bcap29 |  |  |  |  |  |  |
| Q9CY62 | Rnf181 |  |  |  |  |  |  |
| Q9Z2W0 | Dnpep |  |  |  |  |  |  |
| P55264 | Adk |  |  |  |  |  |  |
| Q4FZF3 | Ddx49 |  |  |  |  |  |  |
| Q7TQH0 | Atxn2l |  |  |  |  |  |  |
| Q8K4Z5 | Sf3a1 |  |  |  |  |  |  |
| Q99MR6 | Srrt |  |  |  |  |  |  |
| P17809 | Slc2a1 |  |  |  |  |  |  |
| Q07076 | Anxa7 |  |  |  |  |  |  |
| Q8BNV1 | Trmt2a |  |  |  |  |  |  |
| B2RY56 | Rbm25 |  |  |  |  |  |  |
| Q99PL5 | Rrbp1 |  |  |  |  |  |  |
| P19096 | Fasn |  |  |  |  |  |  |
| P47915 | Rpl29 |  |  |  |  |  |  |
| Q80UG5 | Sept9 |  |  |  |  |  |  |
