# Supplementary material for: Noncanonical contribution of microglial transcription factor NR4A1 to post-stroke recovery through TNF mRNA destabilization
Source: PLoS Biol. 2023 Jul 24;21(7):e3002199. doi: 10.1371/journal.pbio.3002199 (PMC10365314; doi:10.1371/journal.pbio.3002199)
Supplement: S3 Table — (DOCX) [file pbio.3002199.s010.docx]

| *Tnf* | Forward | CATCTTCTCAAAATTCGAGTGAC |
| --- | --- | --- |
|  | Reverse | TGGGAGTAGACAAGGTACAACCC |
|  |  |  |
| *Il1b* | Forward | TGCCACCTTTTGACAGTGATG |
|  | Reverse | ATGTGCTGCTGCGAGATTTG |
|  |  |  |
| *Il6* | Forward | GAGGATACCACTCCCAACAGACC |
|  | Reverse | AAGTGCATCATCGTTGTTCATACA |
|  |  |  |
| *Il10* | Forward | GGTTGCCAAGCCTTATCGGA |
|  | Reverse | ACCTGCTCCACTGCCTTGCT |
|  |  |  |
| *Il4* | Forward | GGTCTCAACCCCCAGCTAGT |
|  | Reverse | GCCGATGATCTCTCTCAAGTGAT |
|  |  |  |
| *Tgfb1* | Forward | AACTATTGCTTCAGCTCCACAGAG |
|  | Reverse | AGTTGGATGGTAGCCCTTG |
|  |  |  |
| *Gapdh* | Forward | GCCAAGGCTGTGGGCAAGGT |
|  | Reverse | TCTCCAGGCGGCACGTCAGA |
|  |  |  |
| *Arg1* | Forward | GTGAAGAACCCACGGTCTGT |
|  | Reverse | GCCAGAGATGCTTCCAACTG |
|  |  |  |
| *Mrc1* | Forward | CAAGGAAGGTTGGCATTTGT |
|  | Reverse | CCTTTCAGTCCTTTGCAAGC |
|  |  |  |
| *Nr4a1* | Forward | ATGCCTCCCCTACCAATCTT |
|  | Reverse | TCTGCCCACTTTCGGATAAC |
|  |  |  |
| *Tnf* CDS (m^6^A-RIP-qPCR) | Forward | TGAGGTCAATCTGCCCAAGT |
|  | Reverse | CCCATTCCCTTCACAGAGCAA |
|  |  |  |
| *Tnf* 3'UTR (m^6^A-RIP-qPCR) | Forward | ACTCAGAAACACAAGATGCTGGG |
|  | Reverse | CCAGTGAATTCGGAAAGCCC |
