# Supplementary material for: Noncanonical contribution of microglial transcription factor NR4A1 to post-stroke recovery through TNF mRNA destabilization
Source: PLoS Biol. 2023 Jul 24;21(7):e3002199. doi: 10.1371/journal.pbio.3002199 (PMC10365314; doi:10.1371/journal.pbio.3002199)
Supplement: S4 Table — (DOCX) [file pbio.3002199.s011.docx]

|  | **Age** | **Gender** | **Date of Death** | **Date of Biopsy** | **Cause of Death** |
| --- | --- | --- | --- | --- | --- |
| **Control 1** | 61 | Male | 20 April 2019 | 20 April 2019 | Diabetic nephropathy, Type 2 Diabetes |
| **Control 2** | 64 | Male | 26 August 2019 | 26 August 2019 | Hypertension |
| **Stroke 1** | 60 | Female | 10 May 2019 | 10 May 2019 | Ischemic Stroke |
| **Stroke 2** | 55 | Male | 17 April 2019 | 18 April 2019 | Ischemic Stroke |
